# PKA and AKIP1 interact to mediate cAMP-driven COX-2 expression: A potentially pivotal interaction in preterm and term labour

**DOI:** 10.1371/journal.pone.0252720

**Published:** 2021-06-24

**Authors:** Angela Yulia, Natasha Singh, Alice J. Varley, Kaiyu Lei, Danijela Markovic, Suren R. Sooranna, Mark R. Johnson

**Affiliations:** 1 Chelsea and Westminster Hospital, London, United Kingdom; 2 Institute of Reproductive and Developmental Biology, London, United Kingdom; Shanghai Jiao Tong University, CHINA

## Abstract

Previously, we showed that cAMP increased COX-2 expression in myometrial cells via MAPK. Here, we have extended these observations, using primary myometrial cell cultures to show that the cAMP agonist, forskolin, enhances IL-1β-driven COX-2 expression. We then explored the role of A-kinase interacting protein (AKIP1), which modulates the effect of PKA on p65 activation. AKIP1 knockdown reversed the effect of forskolin, such that its addition inhibited IL-1β-induced COX-2 mRNA expression and reduced the IL-1β-induced increase in nuclear levels of p65 and c-jun. Forskolin alone and with IL-1β increased IκBα mRNA expression suggesting that in the context of inflammation and in the presence of AKIP1, cAMP enhances p65 activation. AKIP1 knockdown reversed these changes. Interestingly, AKIP1 knockdown had minimal effect on the ability of forskolin to repress either basal OTR expression or IL-1β-stimulated OTR mRNA expression. AKIP1 was up-regulated by IL-1β, but not stretch and was repressed by cAMP. The mRNA expression of AKIP1 increased in early labour in tandem with an increase in COX-2 mRNA and protein. AKIP1 protein levels were also increased with inflammation and stretch-induced preterm labour. Our results identify a second important cAMP effector-switch occurring at term in human myometrium and suggest that a hitherto unrecognized interaction may exist between AKIP1, NFκB and AP-1. These data add to the proposition that cAMP acts as a key regulator of human myometrial contractility.

## Introduction

The factors controlling the onset of human labour remain unclear and until we understand the mechanisms involved it is unlikely that we will reduce the high rates of spontaneous preterm labour or improve our ability to induce labour when it is necessary. Key roles have been ascribed to the prostaglandin and oxytocin systems and modulating their activity through the use of their agonists to induce labour and antagonists to inhibit preterm labour have formed the cornerstone of existing management and the focus of ongoing research. However, the factors that control the expression of key components of the prostaglandin and oxytocin systems are not well understood.

Cyclic AMP (cAMP) is an important second messenger in many cell types regulating many essential processes in a range of tissues. It acts via a number of intermediaries including protein kinase A (PKA) and exchange protein directly activated by *cAMP* (Epac) to modulate target molecule activation and alter cell function. In the reproductive system, previous studies demonstrated that elevation of intracellular cAMP/PKA pathway promote myometrial quiescence during pregnancy [1, 2]. In other tissues, cAMP has been shown to be a potent immunomodulator, inhibiting nuclear factor kappa-light-chain-enhancer of activated B cells (NFκB) activity through a variety of mechanisms [3–6] in different cell types including endothelial cells, where cAMP inhibited Interleukin 1 beta (IL-1β) or tumor necrosis factor-alpha (TNFα)-induced E-selectin and vascular cell adhesion molecule *1* (*VCAM*-*1)* expression [7, 8] and in splenic B lymphocytes, where cAMP inhibited the activation both of NFκB downstream of the B cell antigen receptor and Toll-like receptor 4 (4). The effect of cAMP on NFκB activity seems to be mediated primarily via the inhibition of the degradation of IκBα, an endogenous inhibitor of NFκB. NFκB activation has been suggested to play a key role in labour onset, repressing progesterone activity and driving the expression of prostaglandin synthetic enzymes, including cyclooxygenase-2 COX-2, oxytocin receptor (OTR) and pro-inflammatory chemokines involved in both preterm and term labour [9–13]. However, despite the fact that cAMP/PKA appears to repress NFκB activation, we recently showed that cAMP enhances COX-2 expression in myometrial cells via a mitogen-activated protein kinase (MAPK)-dependent pathway [14].

Gao et al. demonstrated that the nuclear PKA scaffolding protein A kinase interacting protein 1 (AKIP1) promotes PKA-dependent p65 ser-276 phosphorylation and nuclear retention of p65, by preferentially targeting PKA to p65-associated promoters [15]. Later they showed that PKA positively affects p65 transactivation in cells expressing high levels of AKIP1, whereas in cells containing low levels of AKIP1, PKA inhibited NFκB activity, suggesting AKIP1 functions as a molecular switch determining whether PKA positively or negatively affects NFκB activity [15, 16]. Here we have extended our previous observations, investigating the effect of cAMP on inflammation induced COX-2 expression and examining the role of AKIP1 in the regulation of cAMP action in human primary myometrial cell cultures. We then demonstrate the relevance of our in vitro findings by relating them to the change seen in human myometrial samples obtained at different stages of pregnancy and during preterm and term labour.

## Results

### Cyclic AMP enhances IL-1β-driven COX-2

**Fig 1:**
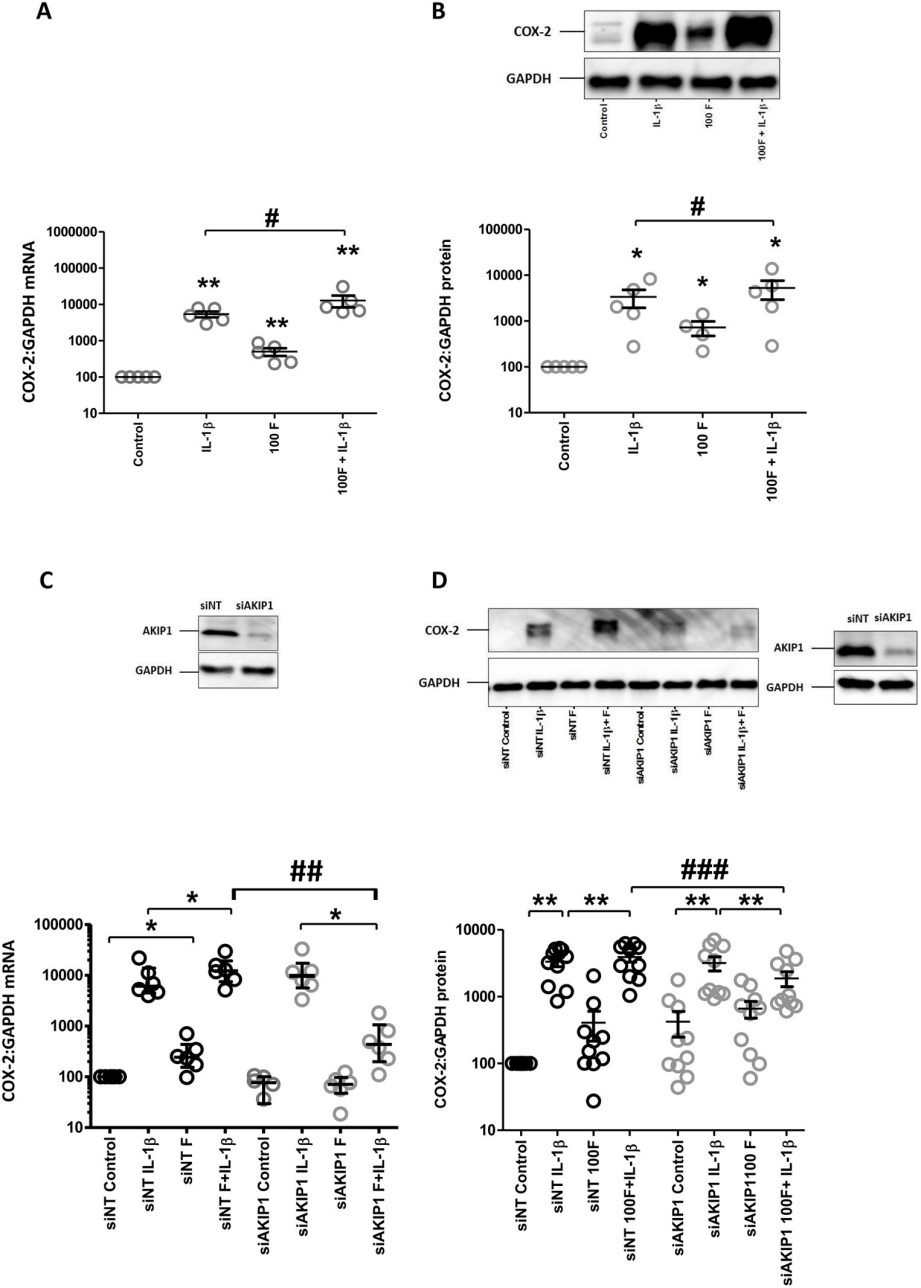
The effect of cAMP on IL-1β-induced Cox-2 gene and protein expression, and the role of AKIP1. Myometrial cells were treated with IL-1β (1ng/mL) and/or forskolin (100μM) either alone or in combination for 24h (A and B). AKIP1 protein expression was knocked down using siRNA (siAKIP1, controlled with non-targeted siRNA [siNT]). 96 hours after transfection the cells were treated with IL-1β (1ng/mL) and/or forskolin (100μM) either alone or in combination for 6h (C and D). mRNA were extracted, and the levels of COX-2 mRNA expression were measured using quantitative rt-PCR (A and C). Proteins were extracted, and the levels of COX-2 protein expression were measured using western (B and D). A representative western blot to demonstrate transfection efficiency is shown on D. Data are shown as the mean and SEM, and analyzed as described in Materials and Methods (*P<0.05, **P<0.01, ***P<0.001 when compared to control; #P<0.05, ##P<0.01, ###P<0.001 when IL-1β is compared to 100F + IL-1β), n = 7–12.

COX-2 is an important regulator of parturition and it is one of labour associated genes, thus we set out to investigate in more detail the role of cAMP in COX-2 regulation. COX-2 mRNA and protein expression were increased by forskolin basally and in combination with IL-1β (Fig 1A and 1B). None of the recognised cAMP-effectors, including PKA, EPAC1 nor AMPK, appeared to mediate the additive effect of forskolin on basal, as we observed previously [14], or IL-1β-induced COX-2 expression (S1A–S1D Fig). In contrast, knock-down of AKIP1 prevented the forskolin-induced increase in basal COX-2 mRNA and protein and converted its incremental effect on IL-1β-induced increase in both COX-2 mRNA and protein levels to an inhibitory one (Fig 1C and 1D).

### Transcription factors involvement

**Fig 2:**
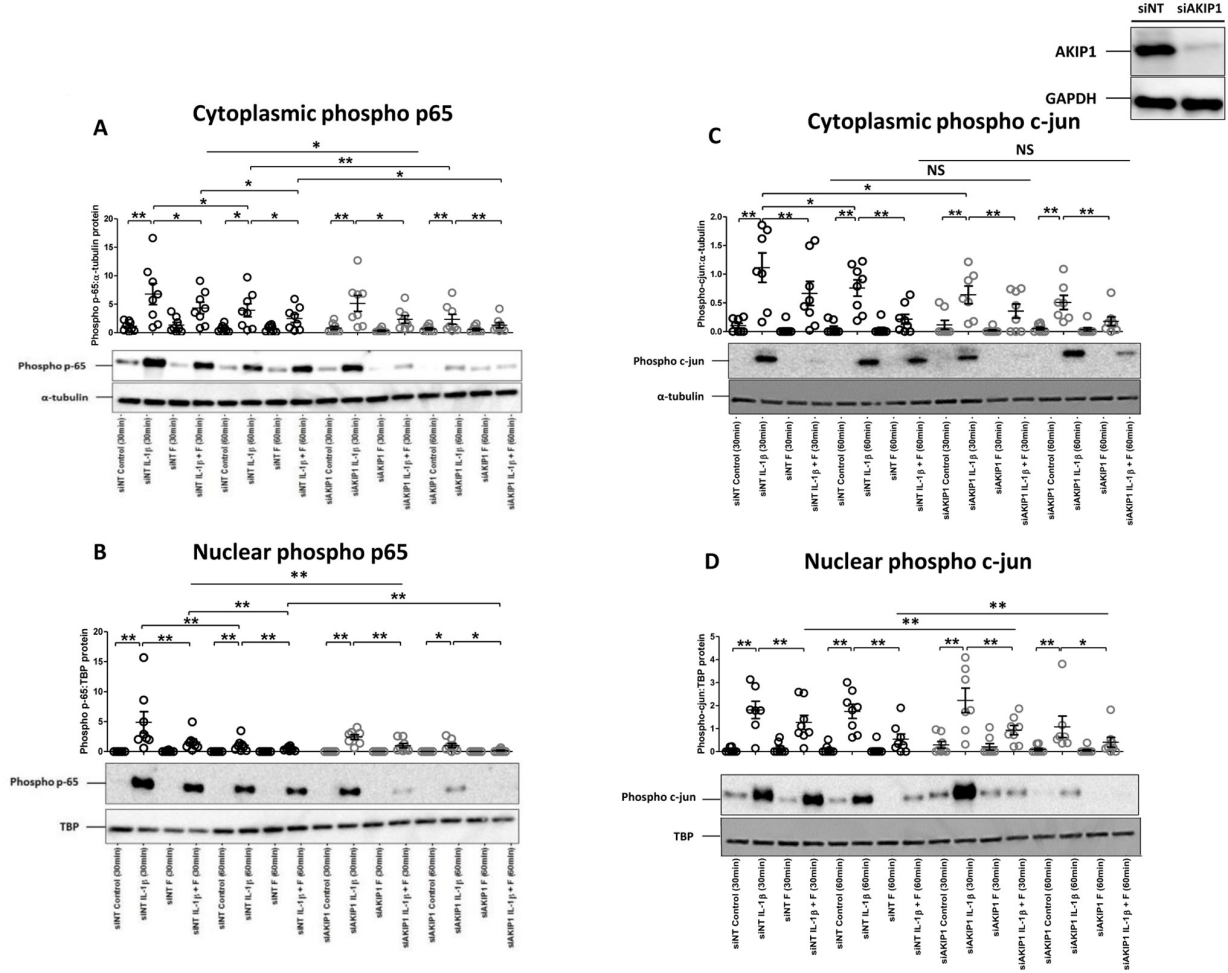
The effects of AKIP1 knock down on the ability of cAMP in repressing IL-1β-induced nuclear transfer of phospho p65 and c-jun. Myometrial cells were transfected with AKIP1 siRNA (siAKIP1), and control siRNA (siNT) as described in Materials and Methods. 96 hours post transfection the cells were treated with IL-1β (1ng/mL) and/or forskolin (100μM) either alone or in combination for 30 minutes and 60 minutes. Cells were lysed and samples purified for cytoplasmic or nuclear protein. Western blotting was performed using antibodies directed against phospho-p65 (Ser536) (A and B) or phospho-cJun (C and D). α-tubulin and TATA-bind protein (TBP) were used as the internal controls for cytosolic (A and C) and nuclear (B and D) fraction, respectively. Data are shown as the mean and SEM *(P<0.05, **P<0.01, ***P<0.001), n = 6–9.

Next, we aimed to investigate the role of AKIP1 in the regulation of transcriptional factors, AP1, NFκB and CREB, known to be crucial in COX-2 regulation. Forskolin reduced IL-1β-induced phosphorylation of p65 in both the cytoplasm and nucleus (Fig 2A and 2B), and this reduction is more significant with the AKIP1 knockdown in both cytoplasm and nucleus at 30 minutes and at 60 minutes. Both cytoplasmic and nuclear phospho-p65 levels were maximal at 30 minutes post IL-1β treatment (Fig 2A and 2B). AKIP1 knockdown reduced IL-1β-induced phopsho-p65, suggesting that AKIP1 enhances not only the nuclear transfer of phospho-p65, but also the initial phosphorylation of p65.

Similarly, forskolin reduced IL-1β-induced phosphoryation of cjun in both the cytoplasm and nucleus (Fig 2C and 2D). We observed that this reduction is more significant with the AKIP1 knockdown compare to siNT in the nuclear level at both 30 minutes and 60 minutes, but not at cytoplasmic level. Cytoplasmic phospho-cjun levels were maximal at 30 minutes post IL-1β treatment. AKIP1 knockdown reduced cytoplasmic IL-1β-induced phospho-cjun levels, but not nuclear levels (Fig 2C and 2D), suggesting novel interactions between the cAMP effector and AP1.

Forskolin alone and in combination with IL-1β increased cytoplasmic and nuclear CREB phosphoryation (S2A and S2B Fig). Both cytoplasmic and nuclear phospho-CREB levels tended to be higher at 30 minutes post IL-1β treatment (S2A and S2B Fig). AKIP1 knockdown had no significant effect on the forskolin-induced increase in cytoplasmic phospho-CREB, but tended to increase the nuclear phospho-CREB (S2A and S2B Fig), suggesting a complex regulation of CREB activation, which will need to be explored further.

### The effect of AKIP1 knockdown on IKBα, MKP-1, IL-8, IL-1 β and OTR mRNA expression

**Fig 3:**
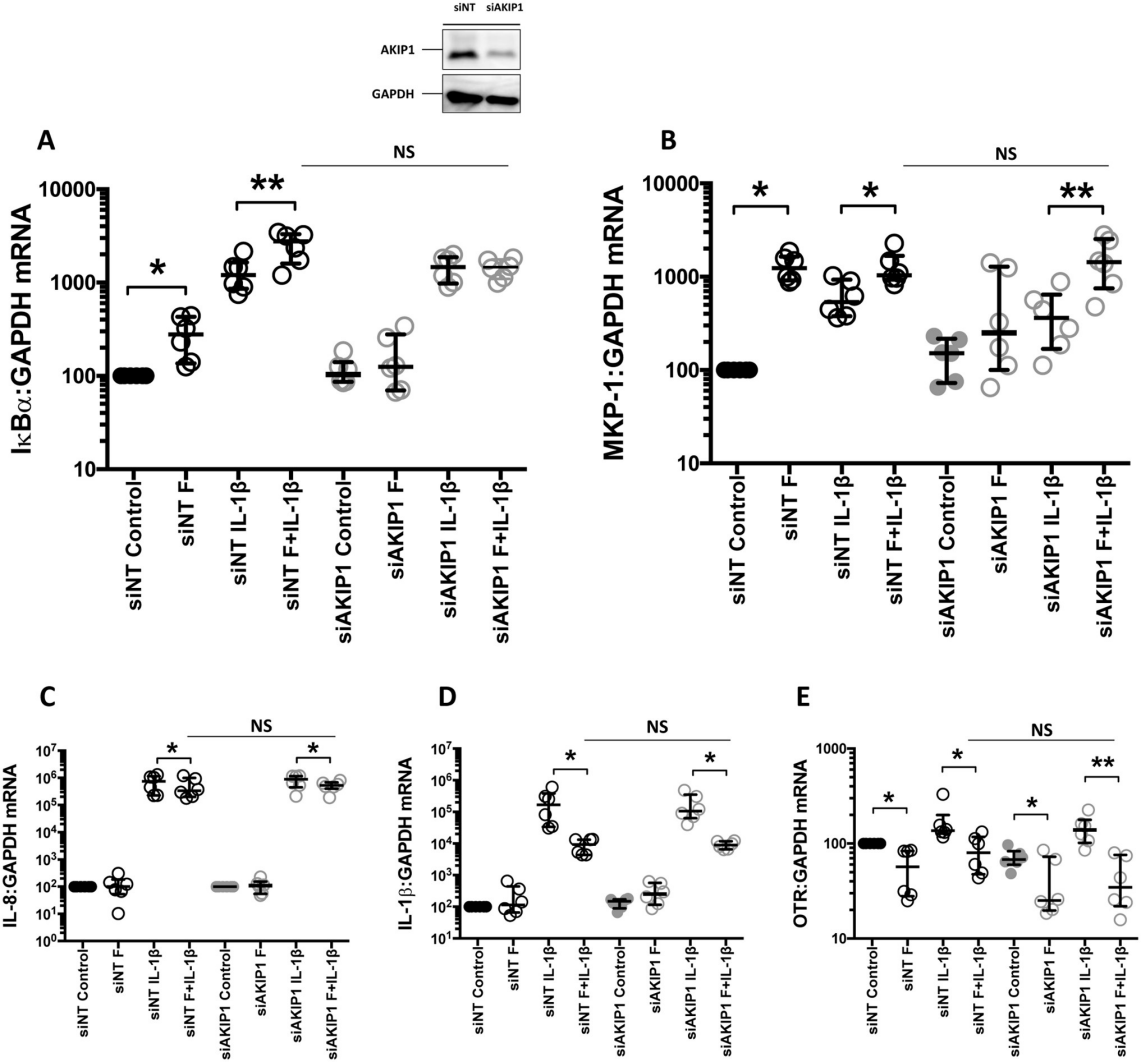
The effects of AKIP1 knock down on IκB, MKP-1, IL-8, IL-1β and OTR mRNA expression. Myometrial cells transfected with AKIP1 siRNA (siAKIP1), and control siRNA (siNT) as described in Materials and Methods. 96 hours post transfection the cells were treated with IL-1β (1ng/mL) and/or forskolin (100μM) either alone or in combination for 6h. mRNA were extracted, and the levels of IκBα (A), MKP-1 (B), IL-8 (C), IL1β (D) and OTR (E) mRNA expression were measured using quantitative rt-PCR. Data are shown as the mean and SEM (*P<0.05, **P<0.01, ***P<0.001 when compared to control; #P<0.05, ##P<0.01, ###P<0.001 when IL-1β is compared to 100F + IL-1β), n = 6.

Further investigations demonstrated that AKIP1 has distinct effects on different genes. Using IKBα mRNA expression as a read-out of NFκB activation, we found that forskolin alone increased IKBα mRNA expression to a lesser extent than IL-1β, but that the combination of forskolin and IL-1β had a greater effect than IL-1β alone (Fig 3A). After knockdown of AKIP1, forskolin no longer increased basal or IL-1β stimulated IKBα mRNA expression, suggesting that AKIP1 is essential for the forskolin-enhancement of NFκB transcriptional activity (Fig 3A).

Forskolin increased basal MKP-1 mRNA expression to a greater extent than IL-1β alone and the combination had a similar effect to that of forskolin alone (Fig 3B). Knockdown of AKIP1 reduced the forskolin and IL-1β-stimulated increase in MKP-1 (Fig 3B). However, with the AKIP1 knockdown, we also observed that the combination of forskolin and IL-1β still increased MKP-1 mRNA expression to a similar extent to the siNT group (Fig 3B), although this increase is not statistically significant compared to the siNT group. The above observation suggests involvement of additional regulatory mechanisms when both stimuli are present.

Next, we explored the transcriptional effects of basal cAMP and its interaction with AKIP1 on the mRNA expression of IL-8, a predominantly NFκB-driven gene, of IL-1β, a mixed NFκB/AP-1-driven gene and of OTR, an NFκB/CEBP-driven gene (Fig 3C–3E). We found that basally, forskolin had no effect on either IL-8 or IL-1β, but as we reported previously [2], it repressed OTR expression (Fig 3C–3E). In all cases, forskolin reduced the IL-1β-induced increase in mRNA expression; in the case of IL-8 or IL-1β, AKIP1 knockdown had minimal effect, but in the case of OTR it appeared that AKIP1 knockdown accentuated the repression of OTR by forskolin, although this repression is not statically significant (Fig 3C–3E).

### Human myometrial tissue AKIP1 and COX-2 levels

**Fig 4:**
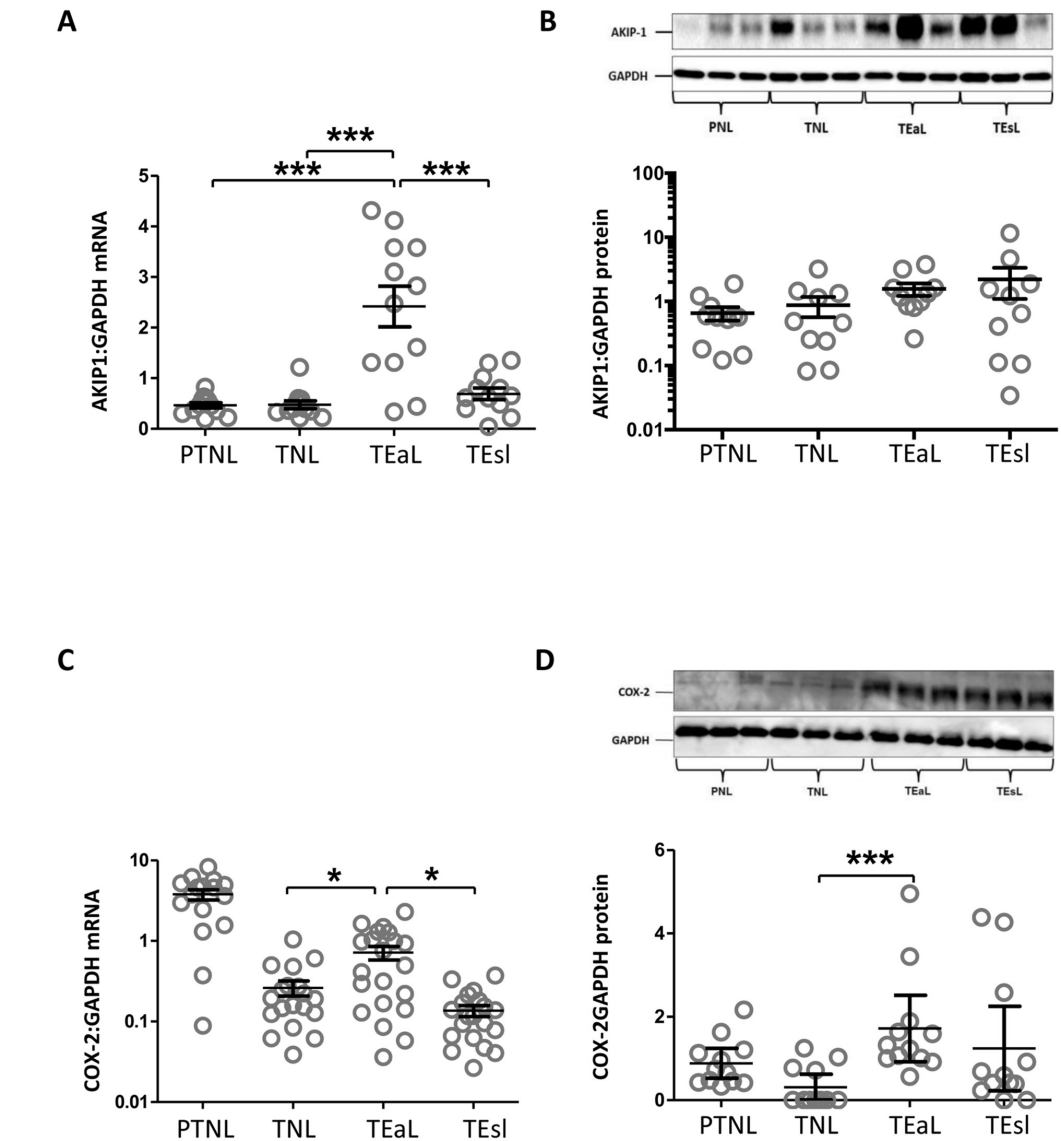
AKIP1 and COX-2 levels are up-regulated with term labour. Human myometrial tissue samples were collected from different groups of non-labouring and labouring women at the time of Caesarean section. Women were recruited in four defined groups: preterm not in labour (PTNL), term not in labour (TNL), term early labour (TEaL) and term established labour (TEsL). Samples were snap frozen at -80°C for mRNA and protein extraction. The levels of AKIP1 (A), and COX-2 (C) mRNA and protein (B and D) were measured using quantitative rt-PCR and western blotting respectively. A representative Western blot is shown above each protein graph displaying the densitometry of the protein levels. Blots were probed with AKIP1 and COX-2 antibody, and GAPDH was used as a loading control. Data are shown as the mean and SEM (*P<0.05, **P<0.01, ***P<0.001), n = 12–20 in each group.

In term myometrial samples, AKIP1 mRNA expression varied with the stage of labour and was highest in the term early labour samples, protein levels rose progressively, but not significantly, as pregnancy advanced and labour started (Fig 4A and 4B). We have regrouped the data where term early labour and term established labour were merged as term labour (S3 Fig). The results showed that AKIP1 expression, was down-regulated during pregnancy and in non-labouring term myometrium. AKIP1 protein levels were significantly increased with the process of labour in term myometrium.

COX-2 mRNA levels were highest in the preterm no labour samples, declined in the term no labour samples before rising again in the term early labour samples; COX-2 protein levels peaked in the term early labour samples too (Fig 4C and 4D). Similar regrouped was done for COX-2; COX-2 protein levels increased with the process of labour in term myometrium (S3 Fig).

**Fig 5:**
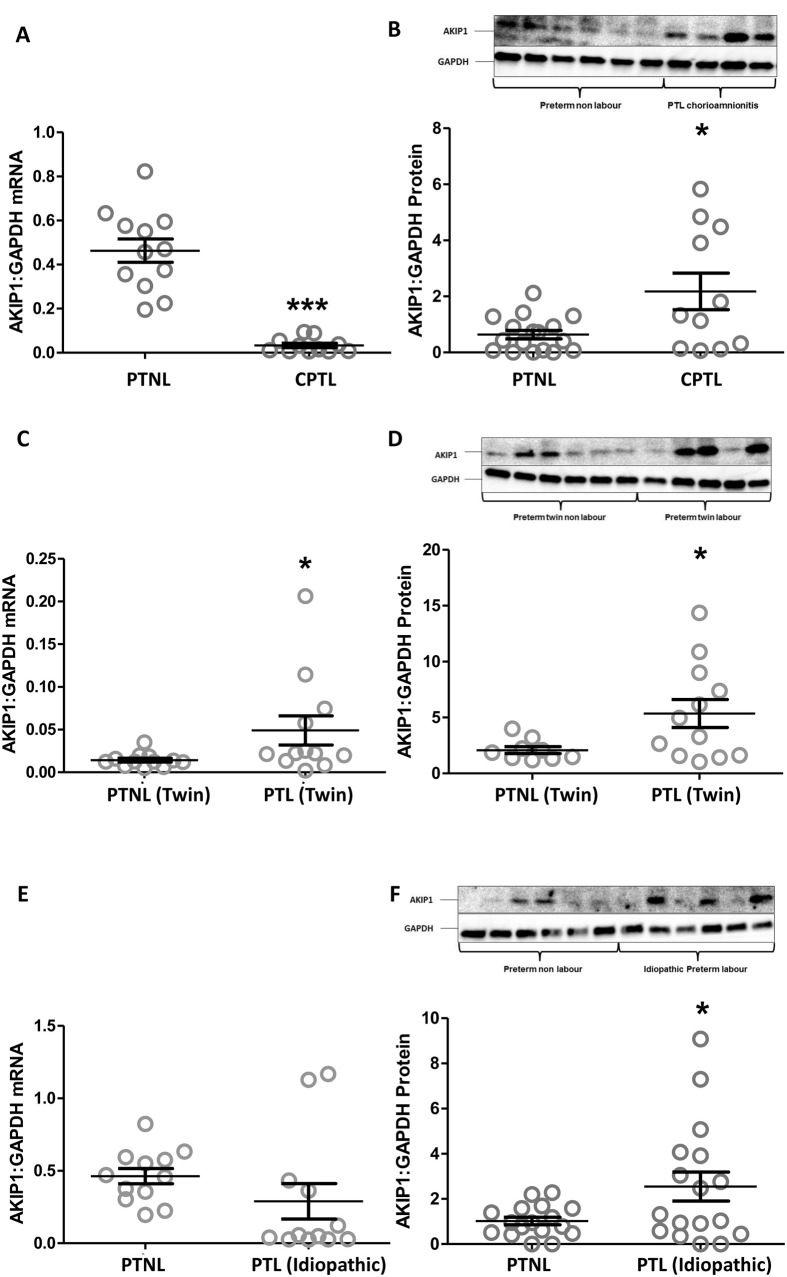
The effect of inflammation and stretch on AKIP1 expression. Human myometrial tissue samples were collected from different groups of non-labouring and labouring women at the time of Caesarean section. Women were recruited in four defined groups: preterm not in labour (PTNL), preterm in labour due to chorioamnionitis (A and B), tweens (C and D) and idiopathic preterm labour (E and F) as stated in Materials and Methods. Samples were snap frozen at -80°C for mRNA and protein extraction. The levels of AKIP1 mRNA (A, C and E), and AKIP1 (B, D and F) proteins were measured using rt-PCR and western blotting respectively. A representative western blot is shown above each protein graph displaying the densitometry of the protein levels. Blots were probed with AKIP1, and GAPDH was used as a loading control. Data are shown as the mean, and SEM (*P<0.05, **P<0.01), n = 11–17 in each group.

In the myometrial samples from women with in early preterm labour due to chorioamnionitis, twins and idiopathic preterm labour, AKIP1 mRNA levels varied, but protein levels were consistently increased when compared to samples from women delivered preterm but not in labour (Fig 5A–5F). Combined these data suggest that an increase in AKIP1 protein levels is mirrored by the onset of the labour, no matter of pathophysiology behind it.

### AKIP1 mRNA is increased by IL-1β and repressed by forskolin

**Fig 6:**
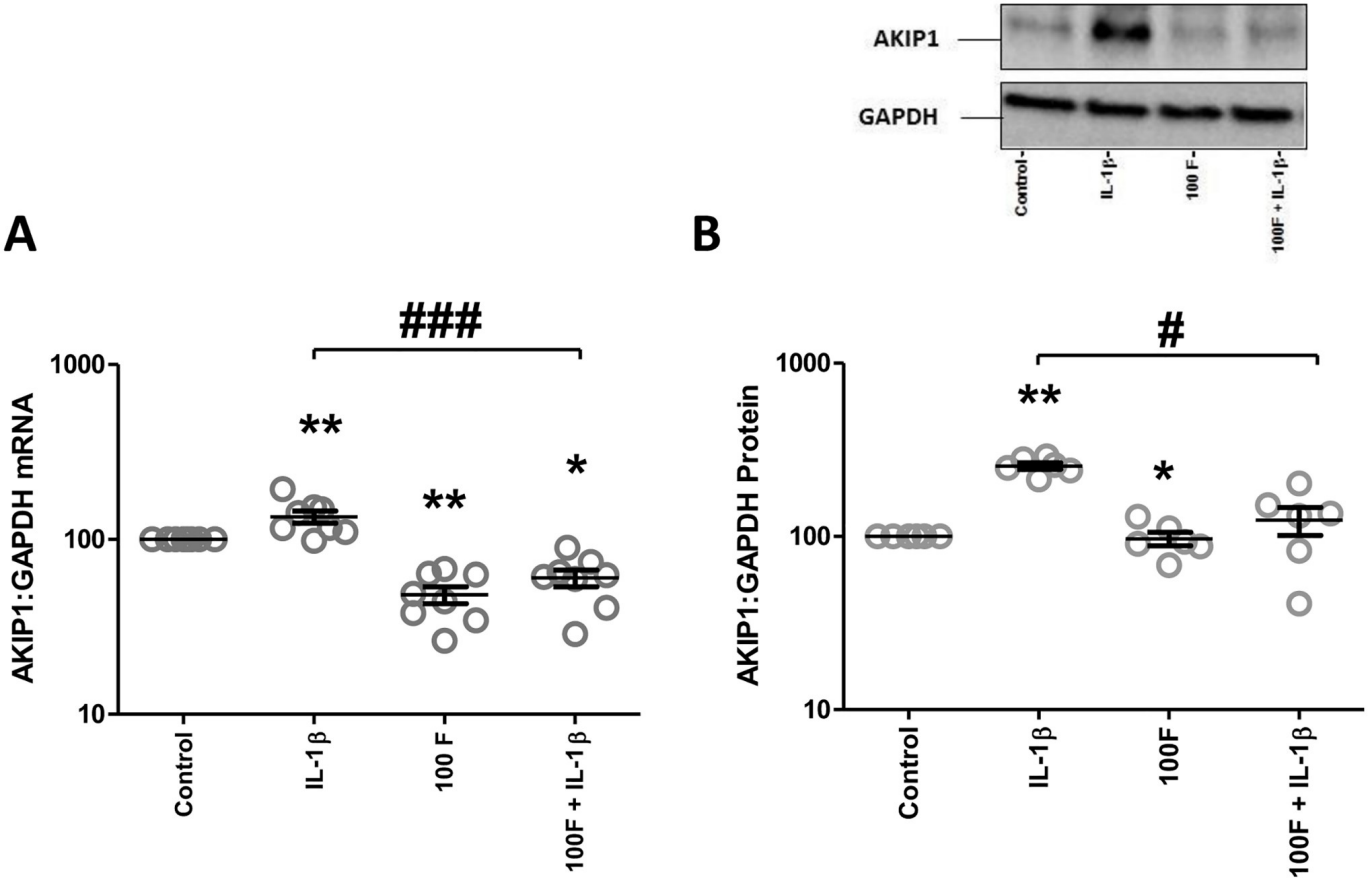
The effect of cAMP in IL-1β driven AKIP1 gene expression. Myometrial cells were isolated as described in Materials and Methods. The cells were treated with IL-1β (1ng/mL) and/or forskolin (100μM) either alone or in combination for 24h. mRNA and proteins were extracted, and the levels of AKIP1 mRNA expression were measured using quantitative rt-PCR (A) and protein levels are assessed by Western blot analysis (B). Data are shown as the mean and SEM (*P<0.05, **P<0.01, ***P<0.001 when compared to control; #P<0.05, ##P<0.01, ###P<0.001 when IL-1β is compared to 100F + IL-1β), n = 8–9.

**Fig 7:**
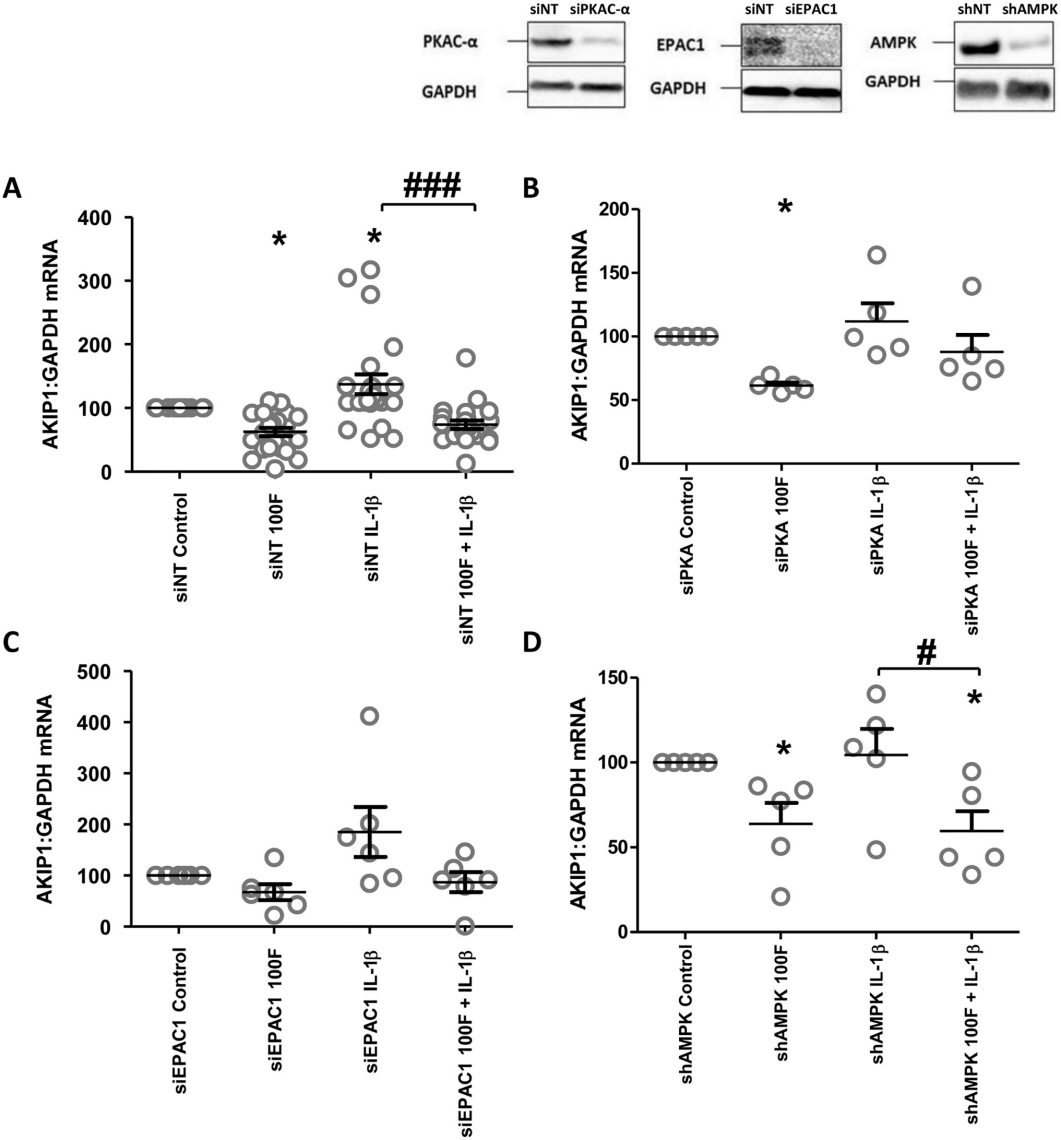
The effect of cAMP effector knockdown on the ability of cAMP in mediating IL-1β-induced AKIP1 mRNA expression. 96 hours post transfection with control siRNA (siNT) (A), PKAc-α siRNA (siPKAc-α) (B), EPAC1 siRNA (siEPAC1) (C), and AMPK siRNA (siAMPK) (D) myometrial cells were treated with IL-1β (1ng/mL) and/or forskolin (100μM) either alone or in combination for 6h as described in Materials and Methods and the levels of AKIP1 mRNA were measured using rt-PCR. Data are shown as the mean and SEM (*P<0.05, **P<0.01, ***P<0.001 when compared to control, #P<0.05, ##P<0.01, ###P<0.001 when IL-1β is compared to 100F + IL-1β), n = 6. Representative western blots to demonstrate transfection efficiency are shown.

**Fig 8:**
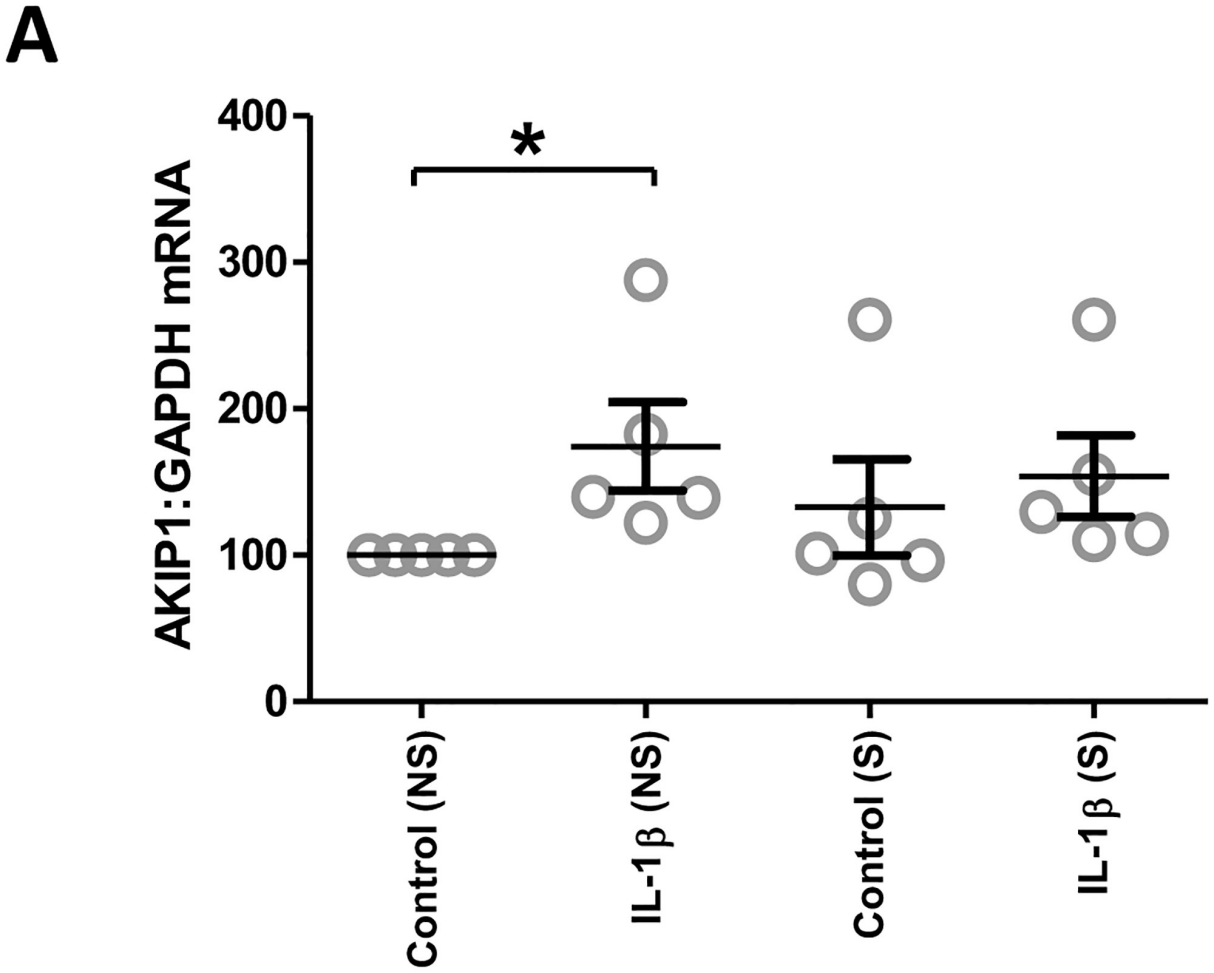
The effect of stretch on AKIP1 expression in vitro. Myometrial cells were isolated from myometrial biopsies obtained from women at the time of pre-labour term Caesarean section. Myometrial cells were grown up to passage 3 and plated on to the stretched plates. Cells were stretched for 30 hours in total. At 24 hours, cells were incubated for an additional 6 hours in the absence and presence of IL-1β (1ng/mL). mRNA were extracted and the level of AKIP1 mRNA (A) were measured using rt-PCR. *P<0.05, n = 6–9.

AKIP1 mRNA expression was reduced by forskolin, but increased by IL-1β (Fig 6A and 6B). The addition of forskolin reduced the IL-1β-induced increase in AKIP1 mRNA and protein (Fig 6A and 6B). In order to explore the potential mechanisms behind forskolin reduced the IL-1β-induced increase in AKIP1 expression, we performed knockdown on cAMP effectors (PKAC-α, EPAC1, AMPK). Forskolin repression of IL-1β-induced increase in AKIP1 mRNA expression did not seem to be mediated by any of the recognized cAMP effectors, since although significance was lost in some cases the pattern of AKIP1 mRNA expression remained the same (Fig 7A–7D). Finally, we investigated the impact of stretch in the presence and absence of IL-1β and found that stretch did not increase basal or IL-1β-induced AKIP1 mRNA expression (Fig 8A).

## Discussion

These data provide more compelling evidence to support the key role played by cAMP and its effector pathways in determining the process of labour onset and progression. Here we show in primary myometrial cell cultures that when levels of the PKA-interacting protein, AKIP1, are low, that cAMP inhibits inflammation driven COX-2 expression. Conversely, when AKIP1 levels are high, then cAMP enhances inflammation driven COX-2 expression. In human myometrial tissues obtained throughout pregnancy, AKIP1 and COX-2 levels are low in term no labour samples and high in laboring samples. Similarly, AKIP1 levels are raised in myometrial samples from 3 phenotypically distinct causes of preterm labour, chorioamnionitis, idiopathic and stretch-induced. In combination, these observations suggest that AKIP1 has a pivotal role in the regulation of human labour.

### Cyclic AMP enhances IL-1β-driven COX-2

In our previous work, we found that various cAMP agonists enhanced COX-2 expression in a MAPK dependent mechanism [14]. Here, we have extended these studies to show that the cAMP-agonist, forskolin, enhanced IL-1β-induced COX-2 expression. Similar to the effects of cAMP alone, the effect of forskolin was independent of any known cAMP effector. Earlier, we found that IL-1β-induced COX-2 expression was driven through the activation of both NFκB and AP-1 [13, 17] and consequently considered whether the nuclear PKA scaffolding protein AKIP1 could have a role in the process. Indeed, AKIP1 knockdown reversed the incremental effect of forskolin on IL-1β-induced COX-2 mRNA and protein levels, reduced the IL-1β-driven phosphorylation and nuclear transport of p65, consistent with previous reports in other tissues [15]. Consistent with the idea that AKIP1 mediates the cAMP-induced increase in NFκB activity, AKIP1 knockdown inhibited the forskolin’s ability to increase basal and IL-1β-induced increase in IκBα mRNA levels, again as described in other tissues [18].

These data suggest that AKIP1 functions as a molecular switch in human myometrial cells, determining whether cAMP/PKA activates or represses NFκB activity. However, the response of IL-8, IL-1β and OTR gene expression to AKIP knockdown was not what we had expected. Given that AKIP1 appears to increase NFΚB transcriptional activity, we would have expected the addition of forskolin to have enhanced IL-1β-induced IL-8, IL-1β and OTR gene expression [13], but forskolin consistently repressed all three genes to the same degree. This is particularly curious for IL-1β gene expression, which, like COX-2, we have previously identified to be regulated by the combination of NFκB and AP-1 [17], suggesting that the ability of AKIP to modulate gene expression may be unique to COX-2 in myometrial cells.

The effect of AKIP1 knockdown on MKP-1 mRNA expression was explored. We showed that knockdown of AKIP1 reduced the forskolin and IL-1β-stimulated increase in MKP-1, but the combination increased MKP-1 mRNA expression in both siNT and siAKIP1 group. As previously described, mitogen-activated protein kinase (MAPK) activation has been implicated in the onset of parturition [19]. MKP-1 catalyses the dephosphorylation of active MAPK, limits activation of down-stream transcription factors, and reduces c-jun binding to the COX-2 promoter [20]. Published findings from our group by Lei *et al*. have recently reported that progesterone acts via GR-induced increase in MKP-1 activity to repress IL-1β-driven COX-2 mRNA expression [17]. Intriguingly, AKIP1 knockdown also seemed to affect c-jun phosphorylation and seemed to reduce c-jun nuclear retention to a degree. The above findings suggest a possible novel interaction between the AKIP1, MKP-1 and AP1.

CREB is also activated by cAMP/PKA and binds to the COX-2 promoter. We found that forskolin induced CREB phosphorylation, which was enhanced by IL-1β. Previous studies by Gao et al. demonstrated that the differential PKA-mediated phosphorylation of CREB and p65, and therefore their recruitment of coactivators such is CBE/p300, is modulated by AKIP1. Thus, we expected that since AKIP knockdown leads to p65 reduced activation, it would lead to the additional enhancement in CREB phosphorylation. However, the data proved to be more complex. We showed that nuclear phospho-CREB levels tend to increase in the absence of AKIP, which is consistent with the observations of Gao et al. However, the knockdown of AKIP1 did not significantly alter phosphorylation levels of cytoplasmic CREB compared to control experiments, although the presence of lower as well as higher molecular weight bands was evident in the samples treated with forskolin. The nature of those proteins was not further investigated in this study, but it is tempting to speculate that the lower band represent increased ATF-1 phosphorylation (since many of the phospho-Ser133-CREB antibody cross-react with ATF1 phosphorylation), which would implicate the role of AKIP1 in mediating activation of another transcriptional factor. The nature of the larger band could be attributed to the phospho-CREB [21]. The significance of these observations is unclear, but they represent another level of transcriptional complexity, which will need to be explored further.

### AKIP1 and the onset of labour

We found that AKIP1 levels are low in human myometrial tissues during pregnancy and increase with the onset of labour. COX-2 levels behave in a similar fashion, although COX-2 protein levels are difficult to interpret since its degradation is substrate-dependent [22]. Also, the impact of increased COX-2 levels on myometrial contractility will be determined by the expression of the down-stream specific prostaglandin synthases, such as PGES, PGIS, which will determine whether pro-contractile or pro-relaxant prostaglandins will be synthesized. The complexity of the situation was illustrated by the TOCOX study, where a specific COX-2 inhibitor actually increased the risk of preterm labour in a high-risk population [23]. We previously reported that changes in PKA and EPAC expression may be responsible for the increase in OTR expression that occur in early labour [2], these, and the data from the current paper, suggest that cAMP and its effector pathways may play a critical role in the onset of term labour.

We also assessed AKIP1 levels in preterm laboring samples compared to preterm non-labour samples. Clearly, the weakness of this comparison lies in the control samples which are taken at the time of Caesarean section performed most commonly for growth restriction and/or pre-eclampsia. As such these samples may not be the best controls but given that the expression of AKIP1 is similar in the myometrial samples from women at term but not in labour, it seems likely that AKIP1 levels are low in preterm non-labour myometrial samples and rise in preterm labour samples of all phenotypes.

### Myometrial AKIP1 regulation and function

Forskolin reduced AKIP expression and inhibited IL-1β-induced expression. From the therapeutic use of a cAMP agonist standpoint, it is reassuring that forskolin repressed AKIP1 expression since if AKIP1 levels are high and a cAMP agonist is given then this would drive COX-2 expression and depending on down-stream PG synthase levels, potentially promote the onset of labour.

### Myometrial COX-2 gene expression

Although it was not statistically significant, there was a trend towards a decline in myometrial COX-2 mRNA and protein from preterm non labour to term non labour group (Fig 4C and 4D. The likely explanation for the trend in COX2 mRNA and protein from preterm non labour to term non labour group; unpublished findings from the group (Singh et al.) showed Our unpublished data found a significant reduction in the expression of downstream enzymes PGF synthase (PGFS) protein from preterm non labour to term non labour group. PGFS is the enzyme that converts PGH2 into PGF2α, which plays a key pro-contractile role in the labour process [24]. It is well recognised that the PG pathways play vital roles in the maintenance of pregnancy and the initiation of both preterm and term labour [25]. Indeed, PGI2 has been reported to have a dual role, maintaining myometrial quiescence on the one hand and the other priming the myometrium for the initiation of labour at term [25].

The mechanism of how AKIP1 interacts with cAMP remains to be investigated further. Previous work has investigated the effects of AKIP1 in breast cancer cells [16], but to date, this has not been investigated in myometrium. As labour is believed to be an inflammatory process, AKIP1 may function as a molecular switch in myometrium, promoting NFκB activitation; It has been reported that in breast cancer tissue there are at least three different splice variants of AKIP1 [26], further more these have distinct cellular localization [27] and different affinity binding for PKAc [28] and such could have an impact of NFκB activation. It would be beneficial, to investigate in more detail if and how the splicing of AKIP1 changes during in parturition.

Previously, we have explored the effect of different stages of pregnancy and labour on cAMP level; the data showed that there was an increase in cAMP level from preterm non-labour group to term non labour group before it declined from term non labour group to term early labour group [2]. In this manuscript, we studied AKIP1 gene expression in myometrial tissue. AKIP1 expression increased with the process of labour, reaching its peak in the term early labour group at mRNA level and term established labour group at protein level. The trend in AKIP1 gene expression at the end of pregnancy and labour was mirrored in COX-2 gene expression. Earlier in pregnancy, the higher COX-2 coincided with lower AKIP implying that other factors may be driving its expression at this point. At the end of pregnancy, the changes in AKIP levels could help to explain the changes in COX-2 mRNA expression.

In chorioamnionitis-associated PTL and to a lesser extent in idiopathic-PTL, AKIP1 mRNA levels were reduced and protein levels increased. In contrast, in twin-PTL, labour both AKIP1 mRNA and protein levels are increased. The inconsistencies in mRNA and protein levels have been discussed extensively before, but here they may reflect the different molecular pathways involved and differences in the evolution of labour in the different PTL forms. Consistent with the concept of labour stage being important, we observed a decline in AKIP1 mRNA in established term labour. Clearly, the increases in AKIP1 protein levels may contribute to the onset of labour in these situations.

Most investigators agree that cAMP contributes to myometrial relaxation, but the study of Fetalvero et al., suggested that cAMP may also prime the myometrium, enabling it to respond to a contractile stimulus by increasing the expression of contraction associated proteins connexin 43, α-SMA, h-caldesmon, calponin and SM2-MHC [29]. Similarly, we found that cAMP increased COX-2 expression in myometrium via MAPK, and that PGE2 acts through EP2 to activate MAPK and increase COX-2 expression [14]. In addition, we previously reported that cAMP enhances progesterone action with the implication that the converse may be true, that a decline in cAMP/PKA action may initiate a functional progesterone withdrawal [30]. More recently, we reported that cAMP repressed OTR expression via PKA, but that with the onset of labour the levels of PKA fell and the repressive effect of cAMP reversed such that cAMP, acting via EPAC, now increased OTR expression [2].

These data suggest that AKIP modulates the impact of cAMP/PKA on COX-2 gene expression and so myometrial function. Whether AKIP1’s effect is mediated solely through altered NFκB activity or the activity of other transcription factors such as AP-1 is unclear and awaits further research.

## Materials and methods

### Patient selection

Approval for the collection of myometrial biopsies was obtained from The Riverside Ethics Committee. All specimens were obtained from all patients participating in the study after fully informed, written patient consent. The recruitment date ranged from 1st September 2012 until 31^st^ August 2015. For the preterm and term not in labour sample groups, the suitable patients were approached during pre-operative assessment at least 3 days prior to the planned caesarean section dates. Patient information leaflets were given to the patients, sufficient time was given to allow them to read and ask questions. On the day of the planned Caesarean section, the patients were approached again, and written consent forms were signed and confirmed. For the rest of the myometrial samples obtained from the term and preterm labouring groups, the patients were approached and consented when they arrived in labour. In all cases, sufficient time were given for the patients to understand, ask questions and to give written informed consent for the study. Biopsies were taken from the upper margin of the uterine lower segment incision at the time of uncomplicated caesarean section from different groups of non-labouring and labouring women at Chelsea and Westminster Hospital (London, United Kingdom). Women were recruited from the following defined groups (Table 1): preterm not in labour (PTNL) (24–36 weeks), term not in labour (TNL) (>37 weeks), term early labour (TEaL) (3–4 contractions in 10 minutes and <3cm dilated) and term established labour (TEsL) (3–4 contractions in 10 minutes and >3cm dilated; n = 12 in each group). The indications for lower segment caesarean section in labouring group were slow labour, cephalo-pelvic disproportion, fetal distress, and breech presentation (all without oxytocin infusion), and in the non-labouring group, fetal indication, previous lower segment caesarean section, breech presentation and maternal request.

**Table 1:** Demographic data for term myometrial tissue samples collected.

	Preterm non labour	Term non labour	Term early labour	Term established labour
**No of samples**	**16**	**20**	**21**	**24**
**Mean gestational age in weeks (GA±SD)**	**31.7±2.2**	**39.2±1.3**	**38.5 ±1.1**	**39.3 ±0.9**
**Maternal characteristics**				
**Age/years**	**35.1±7.3**	**34.1±5.6**	**35.1 ±3.4**	**34.5 ±3.4**
**BMI**	**25.3±5.0**	**26.0±6.4**	**22.4 ±2.5**	**22.7 ±3.2**

In the preterm group (Table 2), the samples from the following groups of women were also collected:

1. Preterm labour due to chorioamnionitis (infection-induced preterm labour (24–36 weeks, defined by offensive vaginal discharge, pyrexia, tachycardia, uterine tenderness, raised inflammatory markers in the blood, and/or positive culture in the amniotic fluid and placental)).
2. Preterm twins gestation in labour and in non-labour (24–36 weeks, uncomplicated twin pregnancies delivered by planned Caesarean section or in spontaneous preterm labour with no evidence of infection, abruption or uterine anomaly).
3. Preterm delivery due to placental abruption (24–36 weeks, delivered due to one or more of the following: PV bleeding, shock, abdominal tenderness, compromised fetus, tachysystole, disseminate intravascular coagulation, retro-placental clot).
4. Idiopathic preterm labour (24–36 weeks, women who were delivered preterm with no evidence of infection, abruption or polyhydramnios and excluding fibroids, previous cervical surgery, mid-trimester loss, previous pelvic inflammatory disease or sexually transmitted disease).

**Table 2:** Demographic data for preterm myometrial tissue samples collected.

	PTNL	Chorioamnionitis PTL	Idiopathic PTL	Abruption PTL	Twins NL	Twins PTL
**No of samples**	**13**	**11**	**9**	**6**	**12**	**8**
**Mean gestational age (GA±SD)**	**30.2±3.5**	**29.5±2.9**	**35.4±1.8**	**32.4±3.5**	**35.2±1.1**	**34.7±1.36**
**Maternal characteristics**						
**Age**	**36.2±6.8**	**33.4±5.3**	**34.2±5.3**	**33.8±6.6**	**38±5.1**	**32.8±6.3**
**BMI**	**26.9±4.2**	**24.2±4.8**	**26.0±5.9**	**23.0±2.9**	**22.2±2.9**	**20.8±2.4**

### Tissue specimens and cell culture

Myometrial biopsies (0.5 x 0.5 cm3) were obtained from the groups of women described above and were snap-frozen immediately after collection at -80°C for protein, and mRNA extraction. For myometrial cell culture, the myometrial biopsies from TNL group were then placed into Dulbecco’s modified Eagle’s Medium (DMEM, Invitrogen, Paisley, PA4 9RF) containing L-glutamine and 100 mU/mL penicillin and 100 mg/mL streptomycin and were stored at 4°C for no more than 3h prior to cell preparation for culture. The biopsies were cut into small pieces and digested for about 45 minutes at 37°C in a collagenase solution which included 0.5 mg/mL collagenase 1A and XI (Sigma) and 1 mg/mL bovine serum albumin in DMEM medium (Sigma). Digestion process was stopped by the addition of DMEM supplemented with 7.5% fetal calf serum (FCS). The myometrial tissue suspension was agitated to further disperse the cells. The resulting suspension was then passed through a cell strainer (70μm nylon cell strainer). The individual cells were collected by centrifugation at 3000rpm for 5min. After the remaining media was removed, the cells pellet were grown in DMEM with supplementation of 7.5% FCS, 1% L-glutamine and 1% penicillin-streptomycin at 37°C in an atmosphere consisting of 5% CO2. The cells were passaged when they were 90%-100% confluent. We used the myometrial cells at the third or fourth passages. Prior to initiation of experiments, the cells were serum starved in 1% dextran coated charcoal (DCC), supplemented with L-glutamine, 100 mU/mL penicillin and 100 g/mL streptomycin) for approximately 16–24 hours. In all cases, at the end of the specified experiment time, the medium was removed, and cells were frozen at -80°C for either mRNA or protein extraction.

### Materials

In this study, we use different treatment conditions to perform the experiments, and the final concentrations are:

- IL-1β 1 ng/mL (Sigma-Aldrich Co. Ltd.)
- Forskolin 100 μM (Sigma-Aldrich Co. Ltd.)

### Transient gene transfection

Cells were cultured in 6-well plates to about 80% confluence and then transfected by using primary smooth muscle cells nucleofector kits transfection reagent (Lonza, UK) according to the manufacturer’s protocol. Based on preliminary transfection experiments, after transfection of relevant siRNAs (Table 3) for 24 hours, the medium was changed to one containing 7.5% serum. After another 48 hrs, the cells were serum starved in 1% DCC, supplemented with L-glutamine, 100 mU/mL penicillin and 100 g/mL streptomycin). The next day, the cells were treated under different conditions for 6 hours. At the end of the specified time medium was removed and cells were frozen at -80°C for extraction of either mRNA or protein.

**Table 3:** ON-TARGET plus SMART pool siRNA sequences.

Name	Source
Non-Targeting	ON-TARGET plus Non-Targeting siRNA: Dharmacon, D-001810-01-05
PKAC-α	ON-TARGET plus Human PRKACA (5566) siRNA: Dharmacon, L-004649-00-0005
EPAC1	ON-TARGET plus Human RAPGEF3 (10411) siRNA: Dharmacon, L-007676-00-0005
shAMPK	OriGene Technologies, Rockville, MD, USA
AKIP1	ON-TARGETplus SMARTpool for AKIP1: Dharmacon, L-015631-01-0005

### RNA extraction, cDNA synthesis and rt-PCR

Total RNA was extracted and purified from human myometrial tissue and cells using RNA easy minikits (Catalog No. 74106; Qiagen Ltd., Crawley, West Sussex, UK). After RNA quantification, 1.0 μg was reverse transcribed with oligo dT random primers using MuLV reverse transcriptase (Applied Biosystems Ltd., Warrington, Cheshire, UK). Primer sets for genes are listed in Table 4. Glyceraldehyde-3-phosphate dehydrogenase (GAPDH) was used as the housekeeping gene. Assays were validated for all primer sets by confirming that single amplicons of appropriate size and sequence were generated according to predictions. Quantitative PCR was performed in the presence of SYBR Green (Applied Biosystems Ltd.), and amplicon yield was monitored during cycling in a RotorGene Sequence Detector (Qiagen Ltd., Crawley, UK) that continually measures fluorescence caused by the binding of the dye to double-stranded DNA. Pre-PCR cycle was 10 min. at 95°C followed by up to 45 cycles of 95°C for 20 sec., 58–60°C for 20 sec. and 72°C for 20 sec. followed by an extension at 72°C for 15 sec. The final procedure involves the determination of a melting temperature over the range of 72–99°C rising by 1° steps with a wait for 15 sec. at the first step followed by a wait of 5 sec. for each subsequent step. The cycle in which fluorescence reached a pre-set threshold (cycle threshold) was used for quantitative analyses. The cycle threshold in each assay was set at a level where the exponential increase in amplicon abundance was approximately parallel between all samples. All mRNA abundance data were expressed relative to the amount of constitutively expressed GAPDH.

**Table 4:** Primer pair sequences with gene accession numbers.

Name	Primer sequence (5’–3’)	GenBank/EMBL accession no.
GAPDH	F: tgatgacatcagaaggtggtgaag	BC014085
	R: tccttggaggccatgtaggccat	
COX-2	F:tgtgcaacacttgagtggct	AY151286
	R: actttctgtactgcgggtgg	
MKP-1	F: cagctgctgcagtttgagtc	NM_004417
	R: aggtagctcagcgcactgtt	
IKBα	F: ccagggctattctccctacc	NM_020529
	R: gctcgtcctctgtgaactcc	
OTR	F: agaagcactcgcgcctctt	NM_000916
	R: aggtgatgtcccacagcaact	
AKIP1	F: cctggtcttccctgtgtgat	NM_001206647.1
	R: tgggcaacagagtgagactg	
IL-8	F: gccttcctgattttgcagc	NM_000584
	R: cgcagtgtggtccactctca	
IL-1β	F: gctgaggaagatgctggttc	NM_000576
	R: tccatatcctgtccctggag	

### Whole-cell protein extraction

Monolayers of human myometrial cells were lysed in cell lysis buffer obtained from New England BioLabs (UK) Ltd., scraped and collected in Eppendorf tubes. Samples were centrifuged at 13,000g for 15 min. at 4°C. Supernatant were then transferred and stored at -80°C.

### Cytosolic/nuclear protein extraction

Cells were trypsinised in 0.25% trypsin containing 0.02% EDTA in PBS, and the cell pellet was resuspended with buffer A and incubated on ice for 20min. Lysates were vortexed for 10sec at a high speed followed by centrifugation at 16 000×g for 30sec at 4°C. The supernatants were reserved as the cytosolic protein extracts. The pellets were resuspended in buffer B and incubated on ice for 15min with shaking (~200rpm). Subsequently the suspension was centrifuged for 5min at 16 000×g for at 4C. After the last centrifugation, the supernatant (nuclear extract) was transferred to a separate tube. These can be used directly or stored at -80°C.

### Western blotting

The protein samples were denatured by heating to approximately 80°C for 10 min and ran on a 10% SDS-PAGE for 30 min. at 100 V, and then 40–60 min. at 150 V, before transferring to a Hybond ECL nitrocellulose membrane (GE Healthcare UK Ltd., Little Chalfont, Buckinghamshire, UK). The membrane was blocked with 5% milk protein solution for 1 hr, washed and incubated with the primary antibody overnight at 4°C. Antibodies used are listed on Table 5. Subsequently, the membrane was incubated with the secondary antibody at room temperature for 2 hr. For ECL detection of horseradish peroxidise, ECL plus (GE Healthcare UK Ltd.) was used. Exposure for detection was at 25°C for 1–5 min.

**Table 5:** Antibodies.

Name	Source	Catalogue Number	Antibody ID RRID
GAPDH	Millipore, Watford UK	MAB374	AB 2107445
α-tubulin	Santa-Cruz Biochem, Texas, US	SC-8035	AB 628408
TATA binding protein	Abcam Ltd, Cambridge, UK	Ab818	AB 306337
COX-2	Santa-Cruz, Biochem, Texas, US	SC-1745	AB 631309
Phospho p65	Cell Signalling, New Eng Biolabs, Herts, UK	3031	AB 330559
Phospho c-jun	Cell Signalling, New Eng Biolabs, Herts, UK	54B3	AB 10691676
AKIP1	Abcam Ltd, Cambridge, UK	Ab135996	No RRID listed
PKAC-α	Cell Signalling, New Eng Biolabs, Herts, UK	4782	AB 10698746
Epac1	Cell Signalling, New Eng Biolabs, Herts, UK	4155	AB 1903962
AMPK	Cell Signalling, New Eng Biolabs, Herts, UK	2532	AB 330331
Phospho-CREB	Cell Signalling, New Eng Biolabs, Herts, UK	9191	AB 331606

### Stretch

When cells were approximately 80% confluent, the cells were plated onto six-well, flexible-bottom culture plates precoated with collagen type I in 2 mL of DMEM medium for stretch. After 24–48 hours, the cells were subjected to a static stretch of 11% using a Flexercell strain unit (for more details, please see http://www.flexcellint.com/; Flexcell International Corp). The cells were stretched for a total of 30 hours. After being stretched for 24 hours, the cells were incubated with and without forskolin (100 μΜ) in the absence and presence of IL-1β (1 ng/mL). The cells were then stretched again for 6 hours. Unstretched cells grown and treated similarly were used as controls.

### Statistical analysis

All data were tested for normality using a Kolmogorov–Smirnoff test. Normally distributed data were analysed using a Student’s t-test for two groups and an ANOVA followed by a Dunnett’s or Bonferroni’s post-hoc test for three groups or more. Data that were not normally distributed were analysed using a Wilcoxon matched pair test for paired data and when comparing three groups or more a Friedman’s test, with a Dunn’s multiple comparisons post-hoc test. P <0.05 was considered statistically significant.

## Supporting information

S1 Fig The effect of cAMP effector knockdown on the ability of cAMP in mediating IL-1β-induced COX-2 mRNA expression. 96 hours post transfection with control siRNA (siNT) (A), PKAc-α siRNA (siPKAc-α) (B), EPAC1 siRNA (siEPAC1) (C), and AMPK siRNA (siAMPK) (D) myometrial cells were treated with IL-1β (1ng/mL) and/or forskolin (100μM) either alone or in combination for 6h as described in Materials and Methods and the levels of COX-2 mRNA were measured using rt-PCR. Data are shown as the mean and SEM (*P<0.05, **P<0.01, ***P<0.001 when compared to control, #P<0.05, ##P<0.01, ###P<0.001 when IL-1β is compared to 100F + IL-1β), n = 6–36 (n = 36 for the siNT). Representative western blot to demonstrate transfection efficiency are shown.
(PPT)

S2 Fig The effects of AKIP1 knock down on cAMP and IL-1β-induced CREB phosphorylation. Myometrial cells were transfected with AKIP1 siRNA (siAKIP1), and control siRNA (siNT) as described in Materials and Methods. 96 hours post transfection the cells were treated with IL-1β (1ng/mL) and/or forskolin (100μM) either alone or in combination for 30 minutes and 60 minutes. Cells were lysed and samples purified for cytoplasmic or nuclear protein. Western blotting was performed using antibodies directed against phosphor-CREB. α-tubulin and TATA-bind protein (TBP) were used as the internal controls for cytosolic (A) and *(P<0.05), n = 6.
(PPT)

S3 Fig AKIP1 and COX-2 levels are up-regulated with term labour. Human myometrial tissue samples were collected from different groups of non-labouring and labouring women at the time of Caesarean section. Women were recruited in three defined groups: preterm not in labour (PTNL), term not in labour (TNL), and term labour (TL). The term labour (TL) group consist of the combination of term early labour (TEaL) and term established labour (TEsL). Samples were snap frozen at -80°C for mRNA and protein extraction. The levels of AKIP1 (A), and COX-2 (C) mRNA and protein (B and D) were measured using quantitative rt-PCR and western blotting respectively. A representative Western blot is shown above each protein graph displaying the densitometry of the protein levels. Blots were probed with AKIP1 and COX-2 antibody, and GAPDH was used as a loading control. Data are shown as the mean and SEM (*P<0.05, **P<0.01, ***P<0.001), n = 12–20 in each group.
(PPT)

S1 Raw images (PDF)
