# Supplementary figures and images for: PKA and AKIP1 interact to mediate cAMP-driven COX-2 expression: A potentially pivotal interaction in preterm and term labour
Source: PLoS One. 2021 Jun 24;16(6):e0252720. doi: 10.1371/journal.pone.0252720 (PMC8224895; doi:10.1371/journal.pone.0252720)

## Slide 1
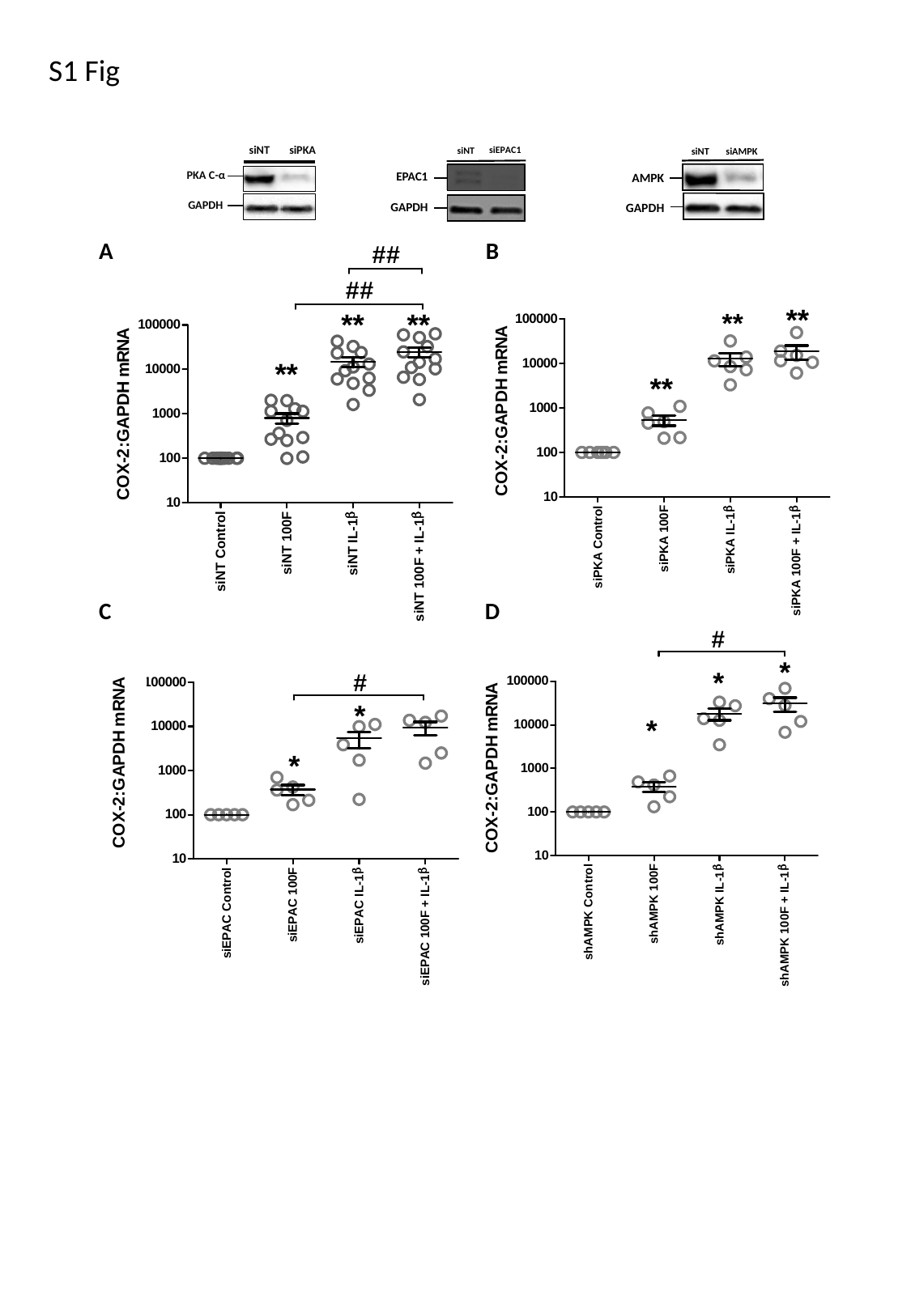

S1 Fig
A
B
C
D

Supplement: S1 Fig — 96 hours post transfection with control siRNA (siNT) (A), PKAc-α siRNA (siPKAc-α) (B), EPAC1 siRNA (siEPAC1) (C), and AMPK siRNA (siAMPK) (D) myometrial cells were treated with IL-1β (1ng/mL) and/or forskolin (100μM) either alone or in combination for 6h as described in Materials and Methods and the levels of COX-2 mRNA were measured using rt-PCR. Data are shown as the mean and SEM (*P<0.05, **P<0.01, ***P<0.001 when compared to control, #P<0.05, ##P<0.01, ###P<0.001 when IL-1β is compared to 100F + IL-1β), n = 6–36 (n = 36 for the siNT). Representative western blot to demonstrate transfection efficiency are shown. (PPT) [file pone.0252720.s001.ppt]

## Slide 1
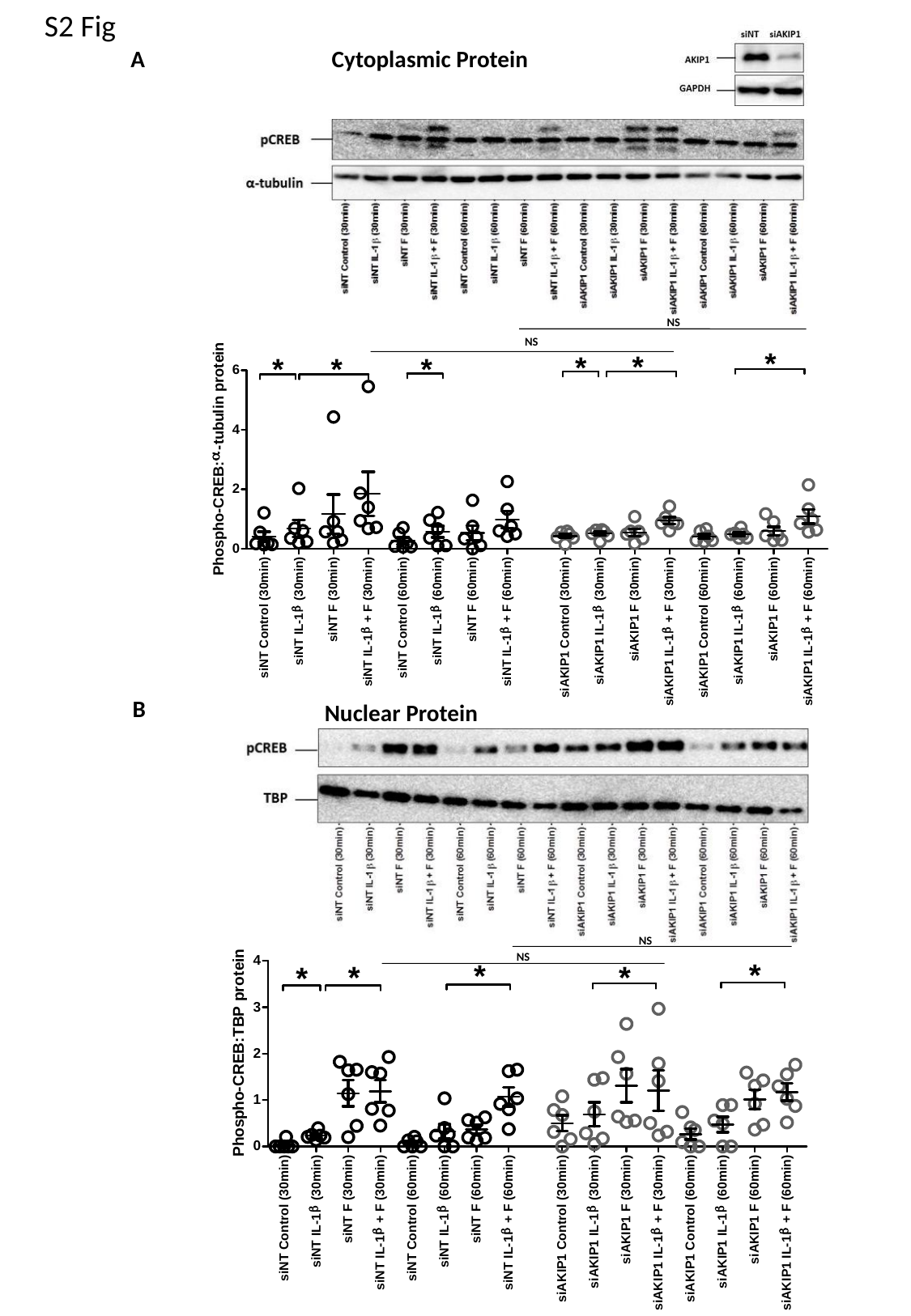

S2 Fig
A
Cytoplasmic Protein
NS
NS
B
Nuclear Protein
NS
NS

Supplement: S2 Fig — Myometrial cells were transfected with AKIP1 siRNA (siAKIP1), and control siRNA (siNT) as described in Materials and Methods. 96 hours post transfection the cells were treated with IL-1β (1ng/mL) and/or forskolin (100μM) either alone or in combination for 30 minutes and 60 minutes. Cells were lysed and samples purified for cytoplasmic or nuclear protein. Western blotting was performed using antibodies directed against phosphor-CREB. α-tubulin and TATA-bind protein (TBP) were used as the internal controls for cytosolic (A) and *(P<0.05), n = 6. (PPT) [file pone.0252720.s002.ppt]
