# Supplementary material for: PKA and AKIP1 interact to mediate cAMP-driven COX-2 expression: A potentially pivotal interaction in preterm and term labour
Source: PLoS One. 2021 Jun 24;16(6):e0252720. doi: 10.1371/journal.pone.0252720 (PMC8224895; doi:10.1371/journal.pone.0252720)
Supplement: S3 Fig — Human myometrial tissue samples were collected from different groups of non-labouring and labouring women at the time of Caesarean section. Women were recruited in three defined groups: preterm not in labour (PTNL), term not in labour (TNL), and term labour (TL). The term labour (TL) group consist of the combination of term early labour (TEaL) and term established labour (TEsL). Samples were snap frozen at -80°C for mRNA and protein extraction. The levels of AKIP1 (A), and COX-2 (C) mRNA and protein (B and D) were measured using quantitative rt-PCR and western blotting respectively. A representative Western blot is shown above each protein graph displaying the densitometry of the protein levels. Blots were probed with AKIP1 and COX-2 antibody, and GAPDH was used as a loading control. Data are shown as the mean and SEM (*P<0.05, **P<0.01, ***P<0.001), n = 12–20 in each group. (PPT) [file pone.0252720.s003.ppt]

## Slide 1
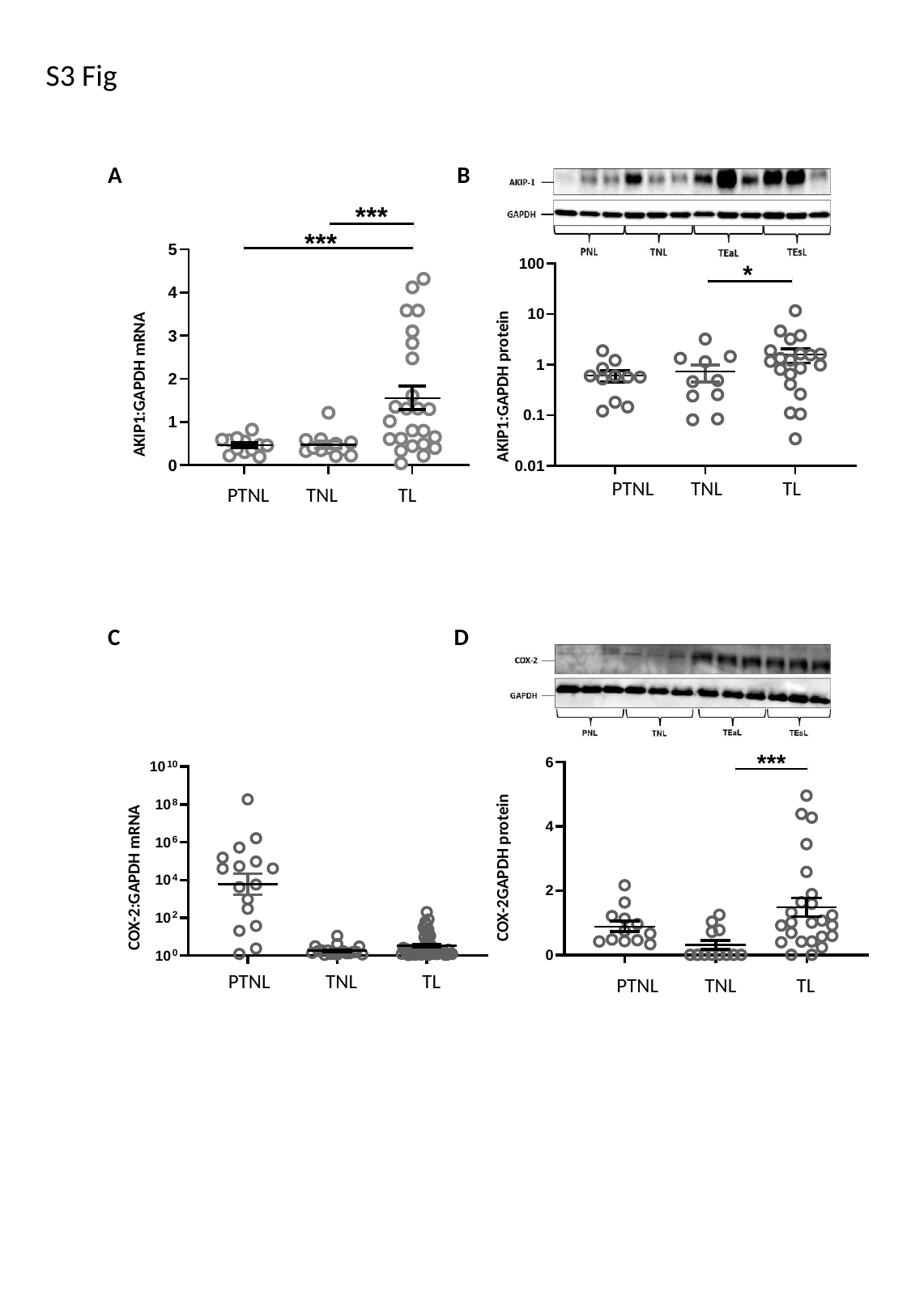

S3 Fig
A
B
AKIP1:GAPDH mRNA
AKIP1:GAPDH protein
PTNL TNL TL
C
D
COX-2GAPDH protein
COX-2:GAPDH mRNA
PTNL TNL TL
PTNL TNL TL
PTNL TNL TL
