# Supplementary material for: PKA and AKIP1 interact to mediate cAMP-driven COX-2 expression: A potentially pivotal interaction in preterm and term labour
Source: PLoS One. 2021 Jun 24;16(6):e0252720. doi: 10.1371/journal.pone.0252720 (PMC8224895; doi:10.1371/journal.pone.0252720)
Supplement: S1 Raw images — (PDF) [file pone.0252720.s004.pdf]

Western Blots

Figure 1b

Sample 1 and 2

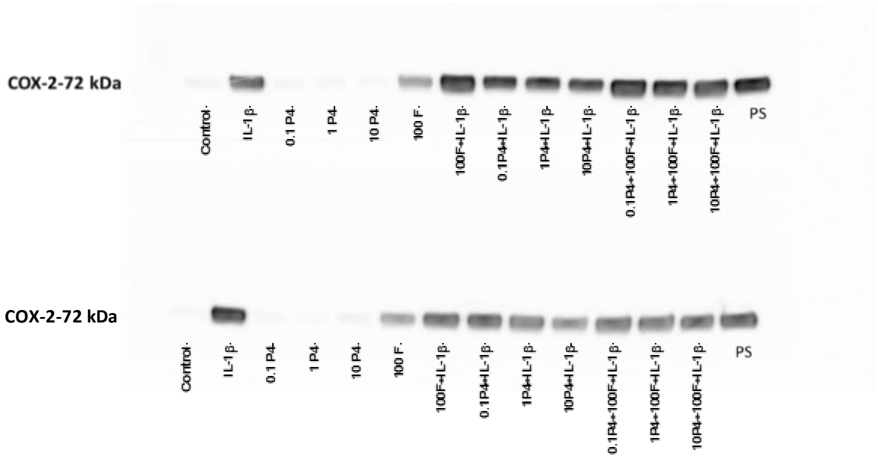

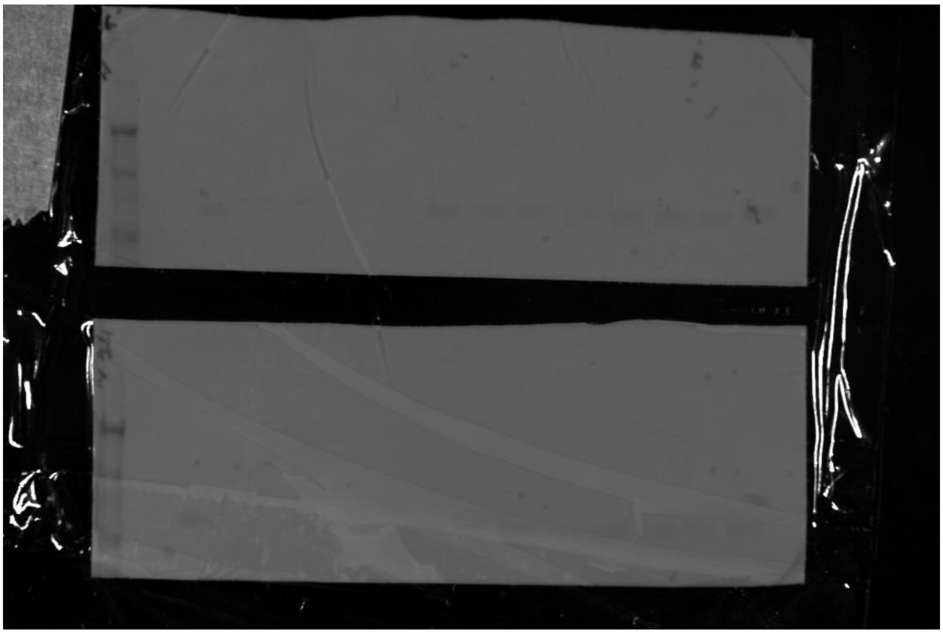

GAPDH-38

GAPDH-38

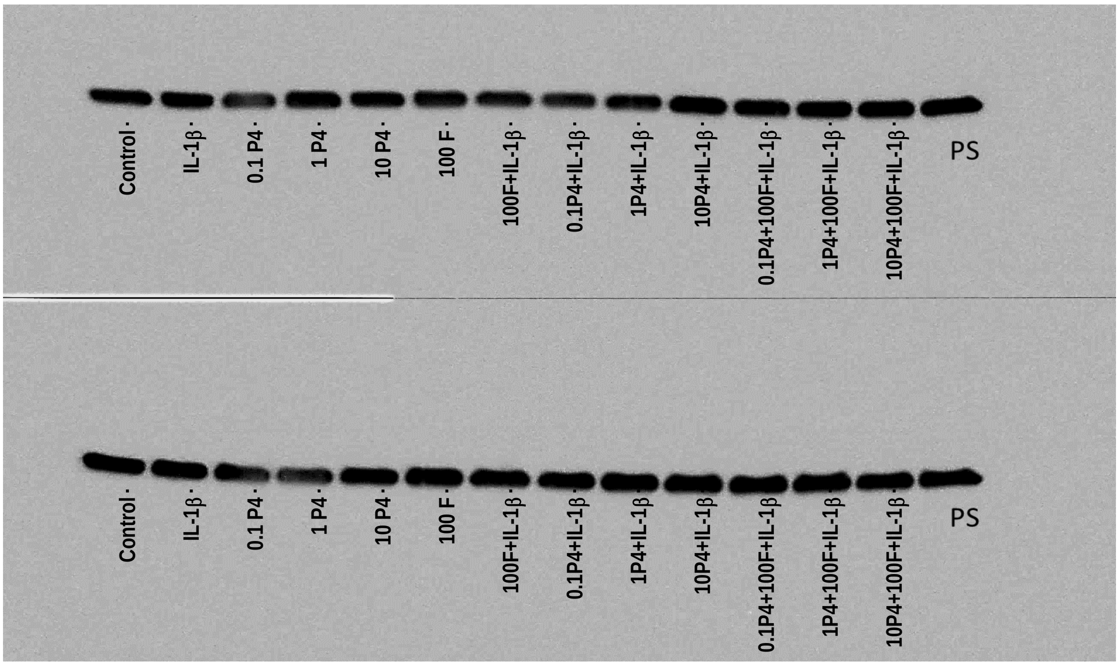

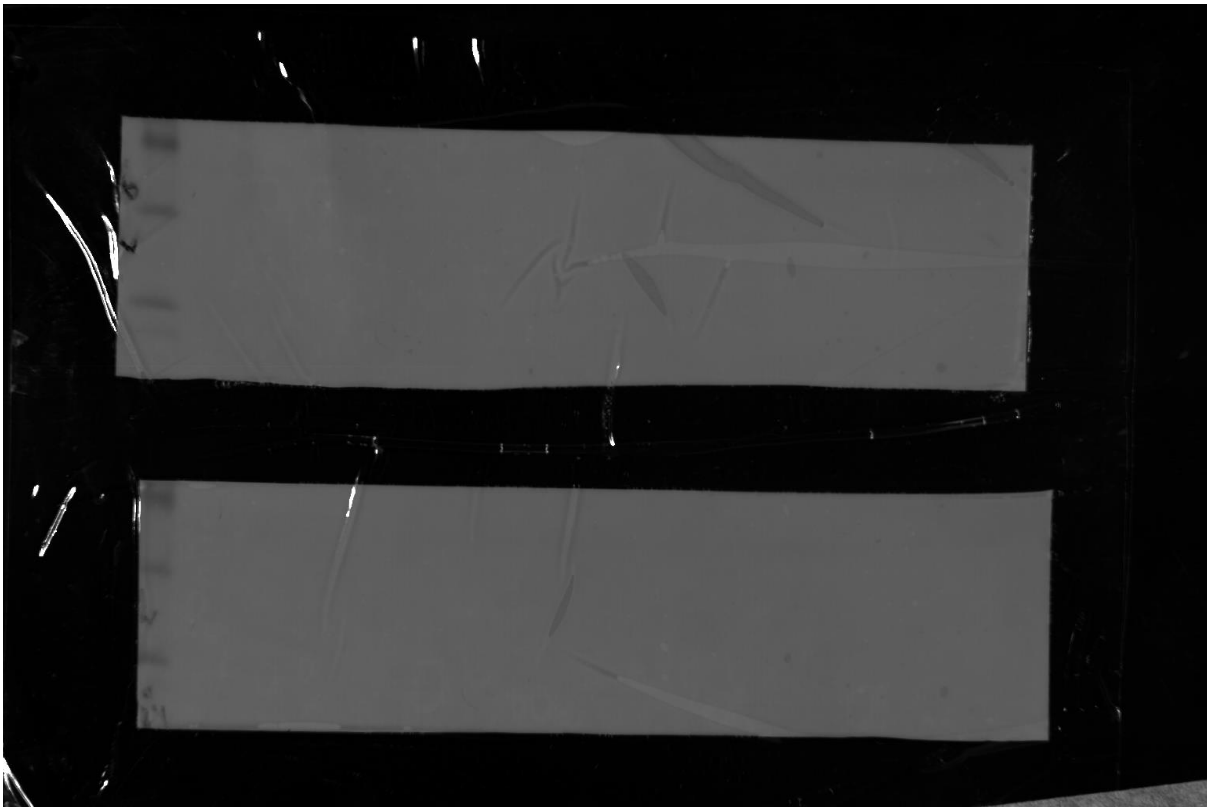

Sample 3-6

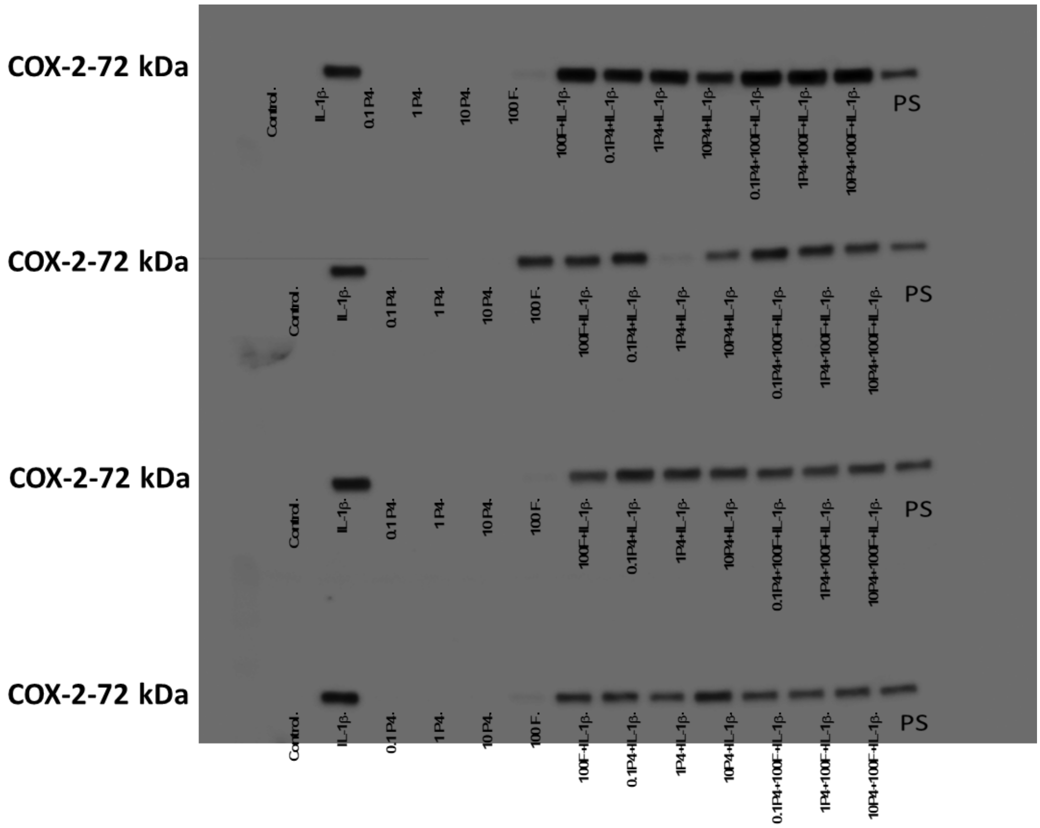
COX-2-72 kDa
Control
IL-1β
0.1 P4
1 P4
10 P4
100 F
100F+IL-1β
0.1P4+IL-1β
1P4+IL-1β
10P4+IL-1β
0.1P4+100F+IL-1β
1P4+100F+IL-1β
10P4+100F+IL-1β
PS
COX-2-72 kDa
COX-2-72 kDa
COX-2-72 kDa

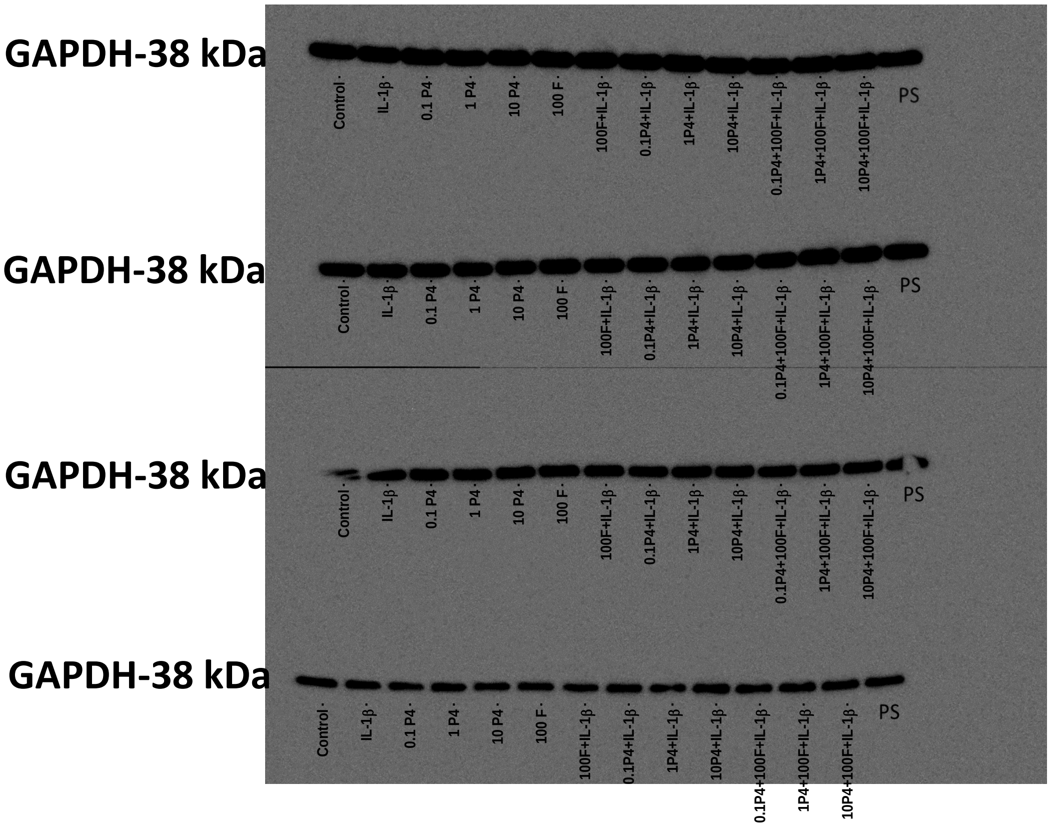
GAPDH-38 kDa
Control
IL-1β
0.1 P4
1 P4
10 P4
100 F
100F+IL-1β
0.1P4+IL-1β
1P4+IL-1β
10P4+IL-1β
0.1P4+100F+IL-1β
1P4+100F+IL-1β
10P4+100F+IL-1β
PS
GAPDH-38 kDa
GAPDH-38 kDa
GAPDH-38 kDa

Sample 7-9

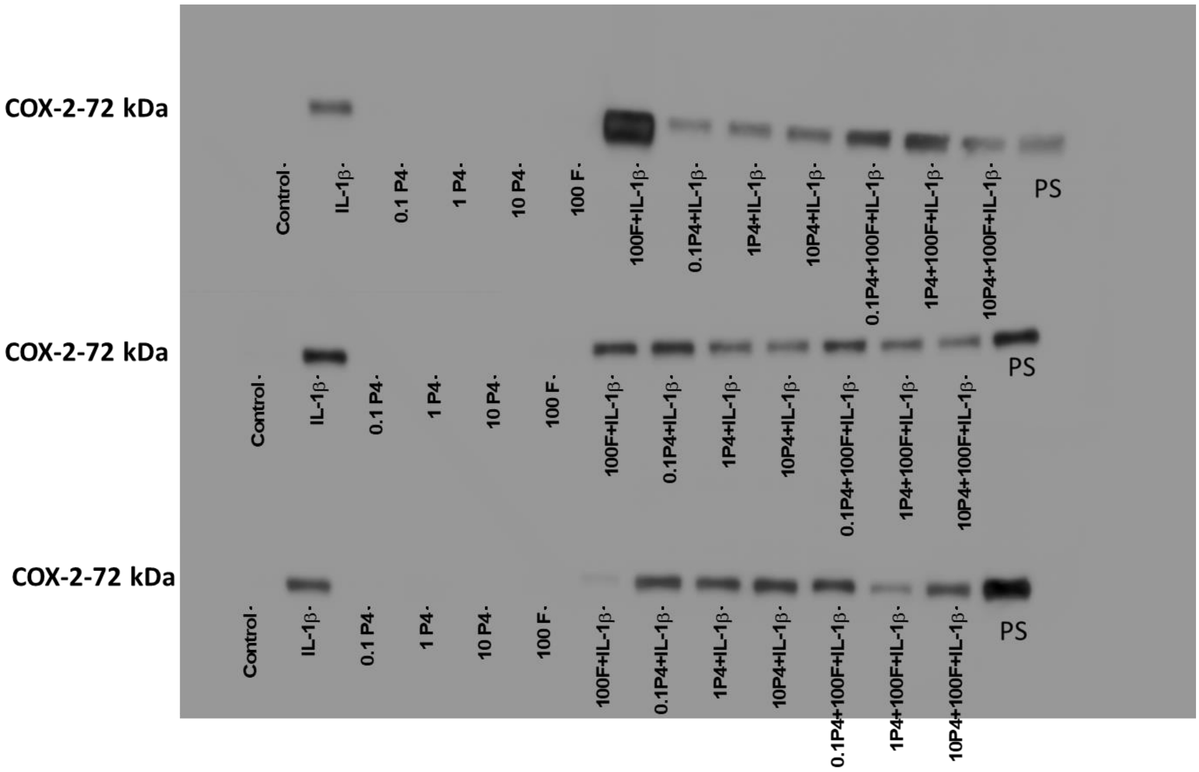

COX-2-72 kDa
Control
IL-1β
0.1 P4
1 P4
10 P4
100 F
100F+IL-1β
0.1P4+IL-1β
1P4+IL-1β
10P4+IL-1β
0.1P4+100F+IL-1β
1P4+100F+IL-1β
10P4+100F+IL-1β
PS
COX-2-72 kDa
Control
IL-1β
0.1 P4
1 P4
10 P4
100 F
100F+IL-1β
0.1P4+IL-1β
1P4+IL-1β
10P4+IL-1β
0.1P4+100F+IL-1β
1P4+100F+IL-1β
10P4+100F+IL-1β
PS
COX-2-72 kDa
Control
IL-1β
0.1 P4
1 P4
10 P4
100 F
100F+IL-1β
0.1P4+IL-1β
1P4+IL-1β
10P4+IL-1β
0.1P4+100F+IL-1β
1P4+100F+IL-1β
10P4+100F+IL-1β
PS

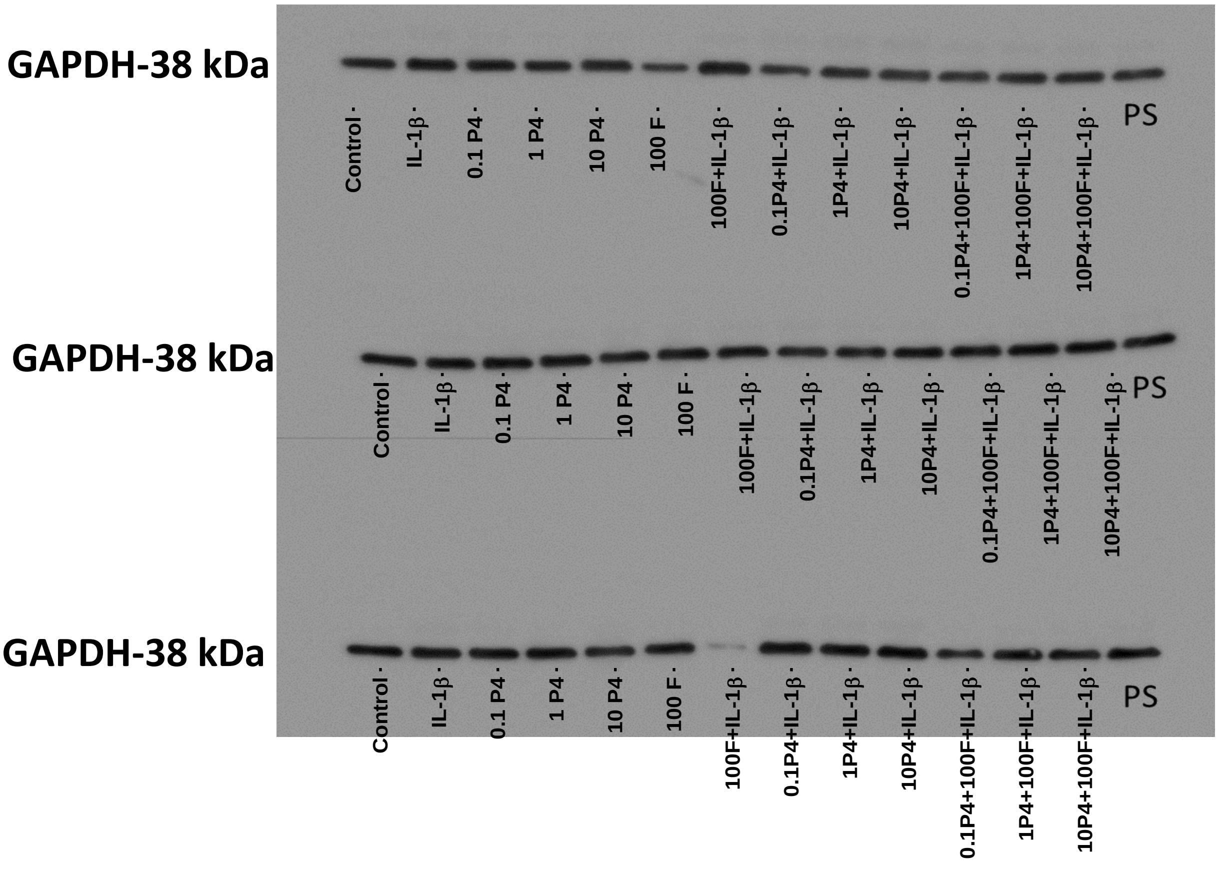

GAPDH-38 kDa
Control
IL-1β
0.1 P4
1 P4
10 P4
100 F
100F+IL-1β
0.1P4+IL-1β
1P4+IL-1β
10P4+IL-1β
0.1P4+100F+IL-1β
1P4+100F+IL-1β
10P4+100F+IL-1β
PS
GAPDH-38 kDa
Control
IL-1β
0.1 P4
1 P4
10 P4
100 F
100F+IL-1β
0.1P4+IL-1β
1P4+IL-1β
10P4+IL-1β
0.1P4+100F+IL-1β
1P4+100F+IL-1β
10P4+100F+IL-1β
PS
GAPDH-38 kDa
Control
IL-1β
0.1 P4
1 P4
10 P4
100 F
100F+IL-1β
0.1P4+IL-1β
1P4+IL-1β
10P4+IL-1β
0.1P4+100F+IL-1β
1P4+100F+IL-1β
10P4+100F+IL-1β
PS

AKIP1 Knockdown (Fig 1C, 1D, 2, 3, Supp 2)

AKIP1 Knockdown

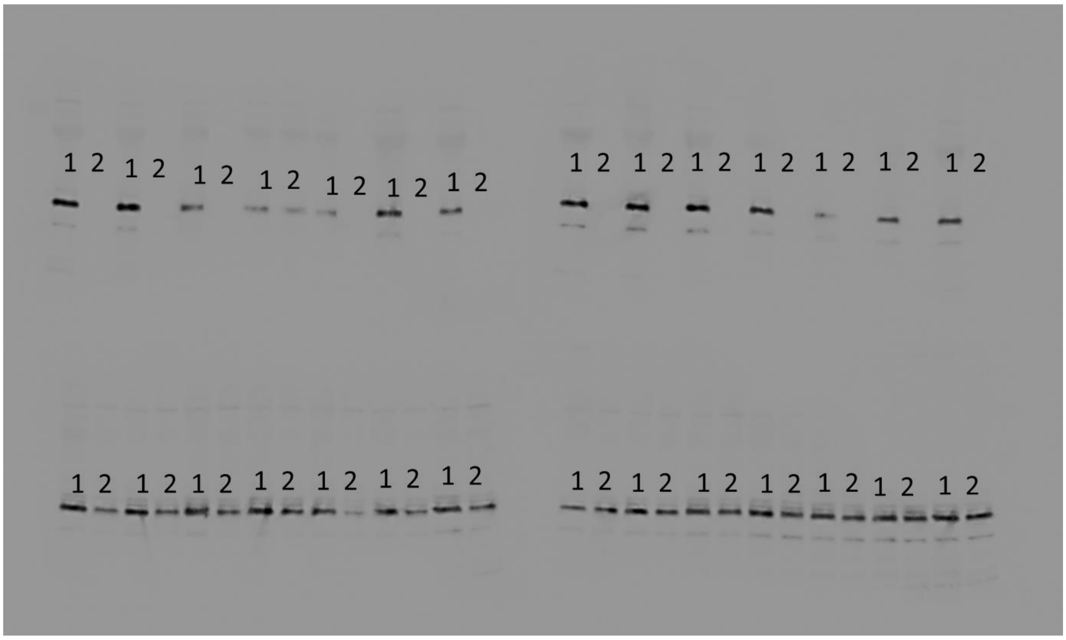

1. siNT Control
2. siAKIP1 Control

AKIP1 Knockdown GAPDH

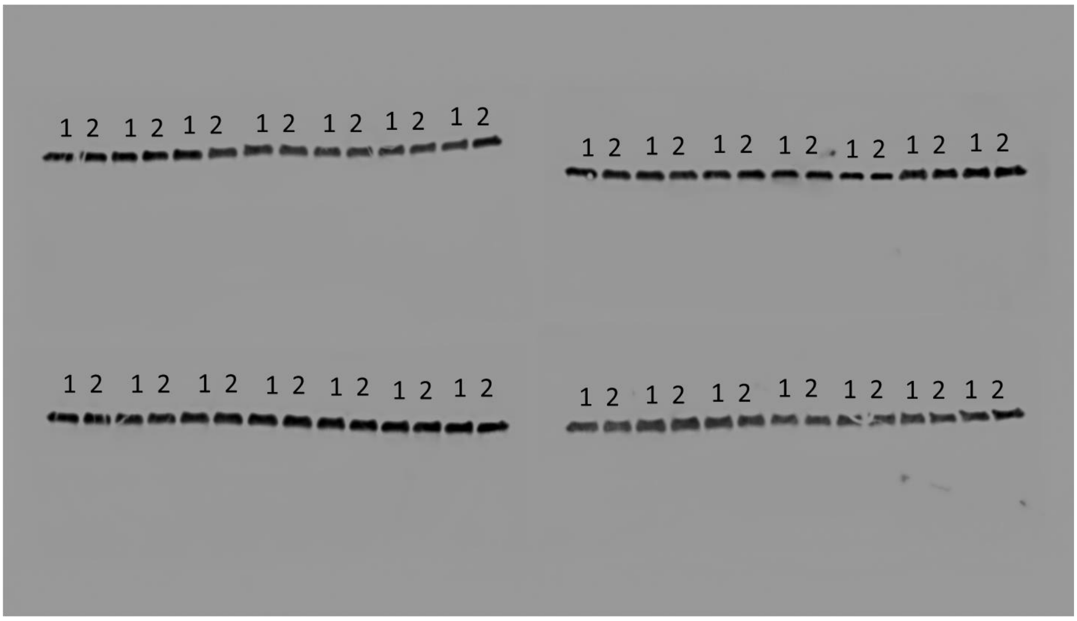

1. siNT Control
2. siAKIP1 Control

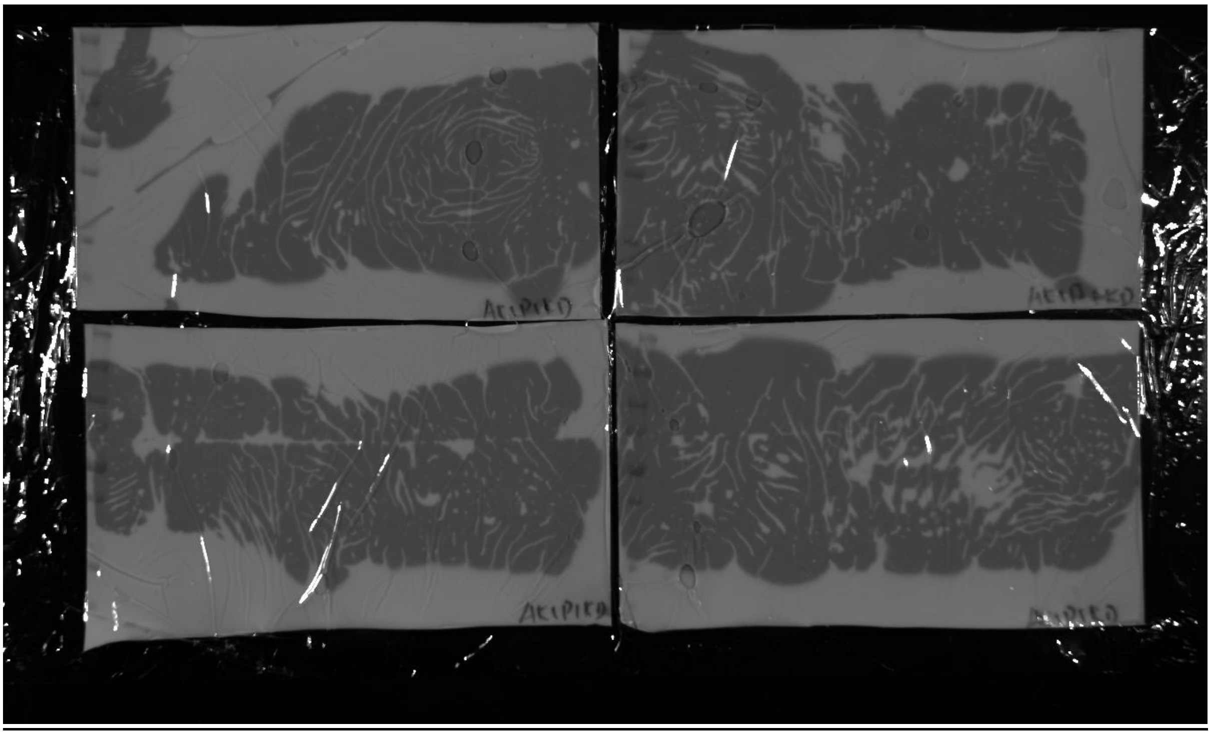

Figure 1d

COX-2-72 kDa

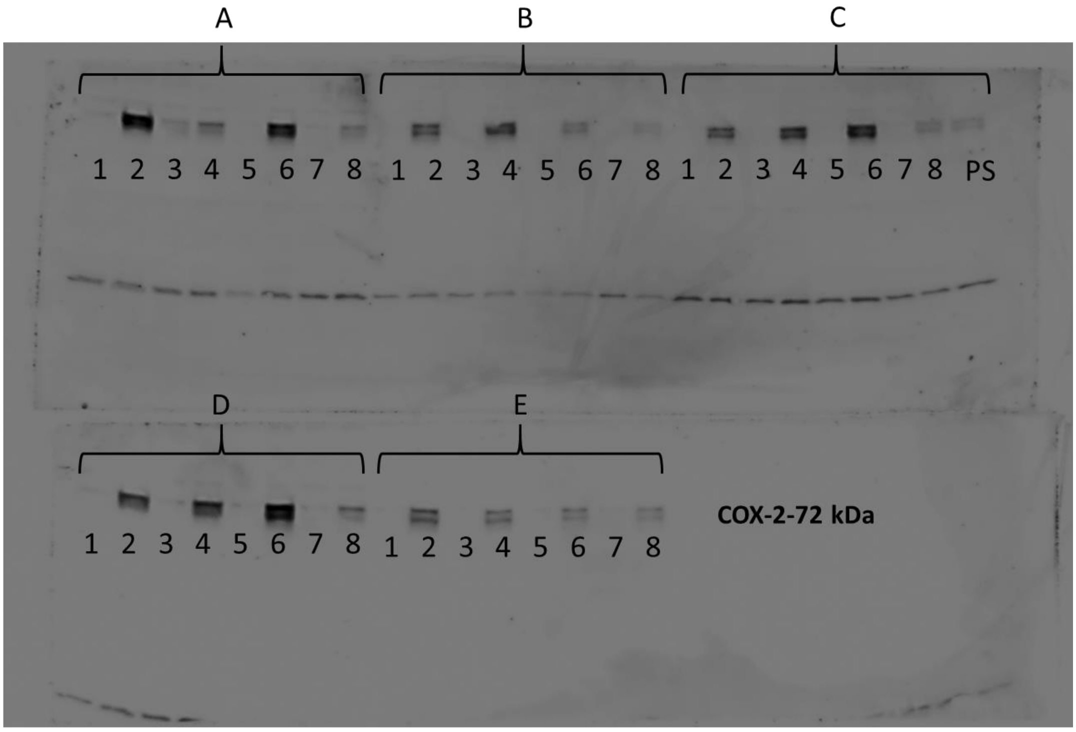

COX-2-72 kDa

1. siNT Control
2. siNT IL-1β
3. siNT Forskolin (F)
4. siNT F+IL-1β
5. siAKIP1 Control
6. siAKIP1 IL-1β
7. siAKIP1 Forskolin (F)
8. siAKIP1 F+IL-1β

PS - Positive Control

GAPDH-38 kDa

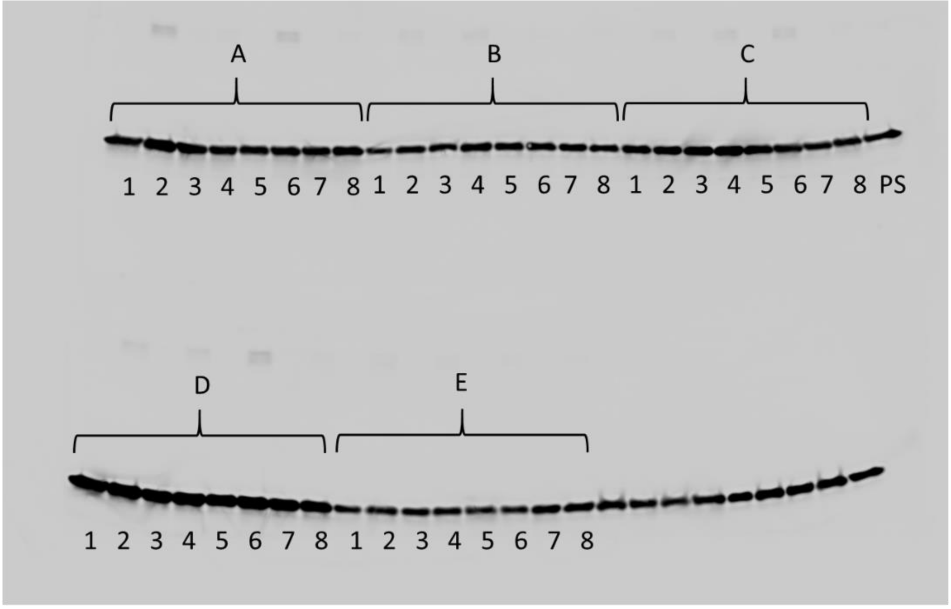

GAPDH-38 kDa

1. siNT Control
2. siNT IL-1β
3. siNT Forskolin (F)
4. siNT F+IL-1β
5. siAKIP1 Control
6. siAKIP1 IL-1β
7. siAKIP1 Forskolin (F)
8. siAKIP1 F+IL-1β

PS - Positive Control

COX-2-72 kDa

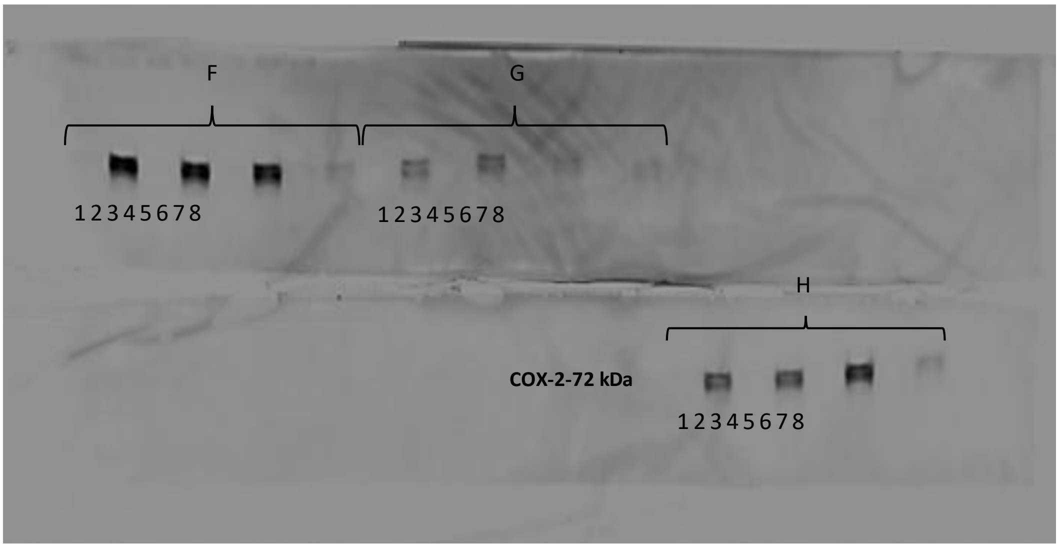

1. siNT Control
2. siNT IL-1β
3. siNT Forskolin (F)
4. siNT F+IL-1β
5. siAKIP1 Control
6. siAKIP1 IL-1β
7. siAKIP1 Forskolin (F)
8. siAKIP1 F+IL-1β

GAPDH-38 kDa

GAPDH-38 kDa

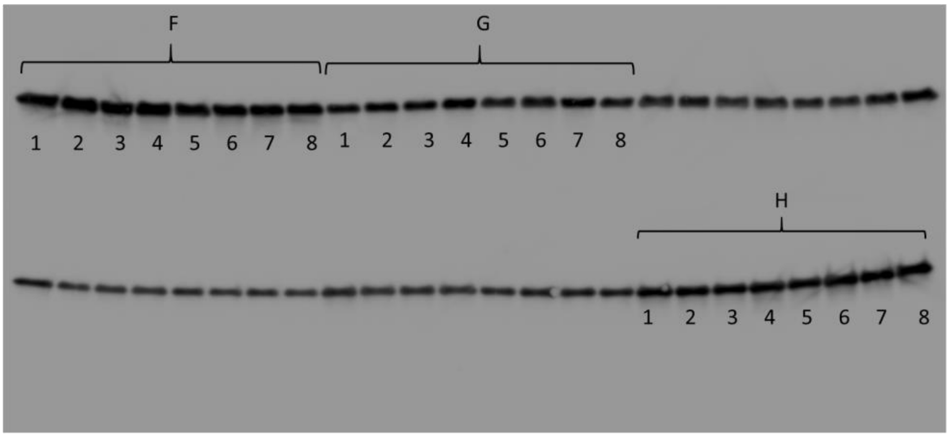

1. siNT Control
2. siNT IL-1β
3. siNT Forskolin (F)
4. siNT F+IL-1β
5. siAKIP1 Control
6. siAKIP1 IL-1β
7. siAKIP1 Forskolin (F)
8. siAKIP1 F+IL-1β

PS - Positive Control

COX-2-72 kDa

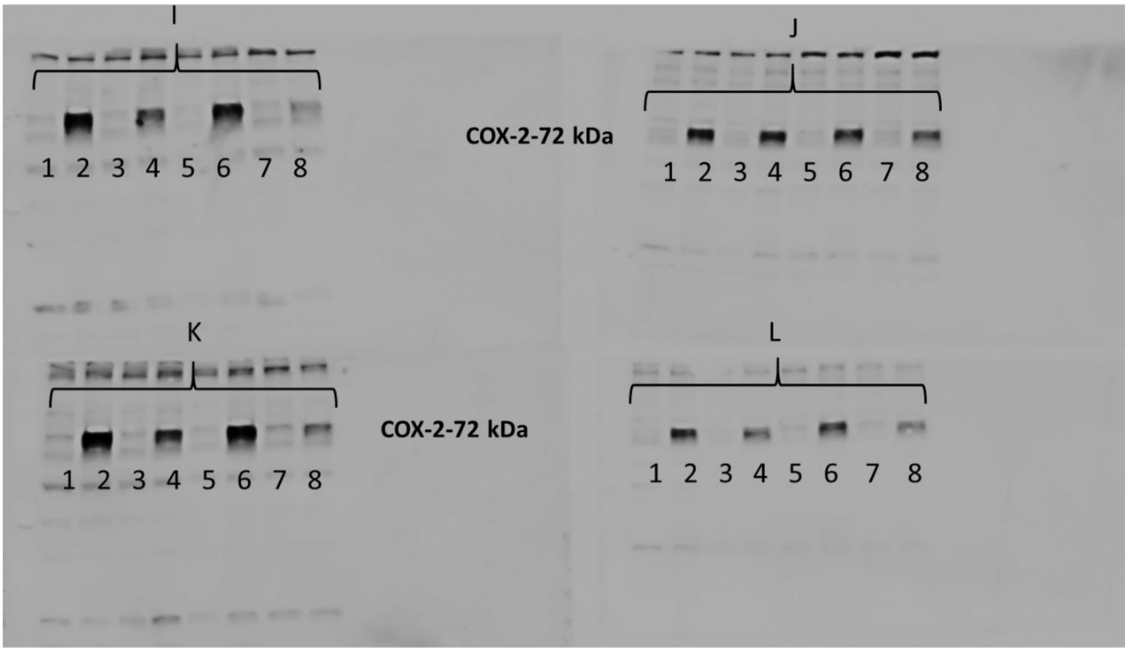

1. siNT Control
2. siNT IL-1β
3. siNT Forskolin (F)
4. siNT F+IL-1β
5. siAKIP1 Control
6. siAKIP1 IL-1β
7. siAKIP1 Forskolin (F)
8. siAKIP1 F+IL-1β

GAPDH-38 kDa

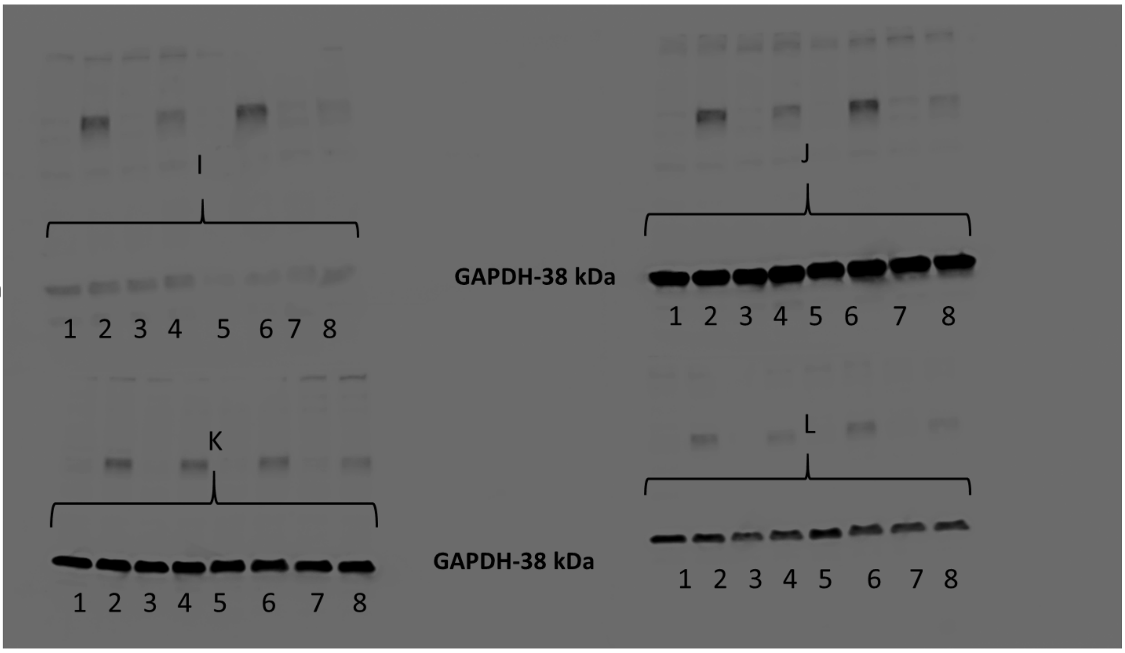

1. siNT Control
2. siNT IL-1β
3. siNT Forskolin (F)
4. siNT F+IL-1β
5. siAKIP1 Control
6. siAKIP1 IL-1β
7. siAKIP1 Forskolin (F)
8. siAKIP1 F+IL-1β

Figure 2a – Cytoplasmic phospho p65

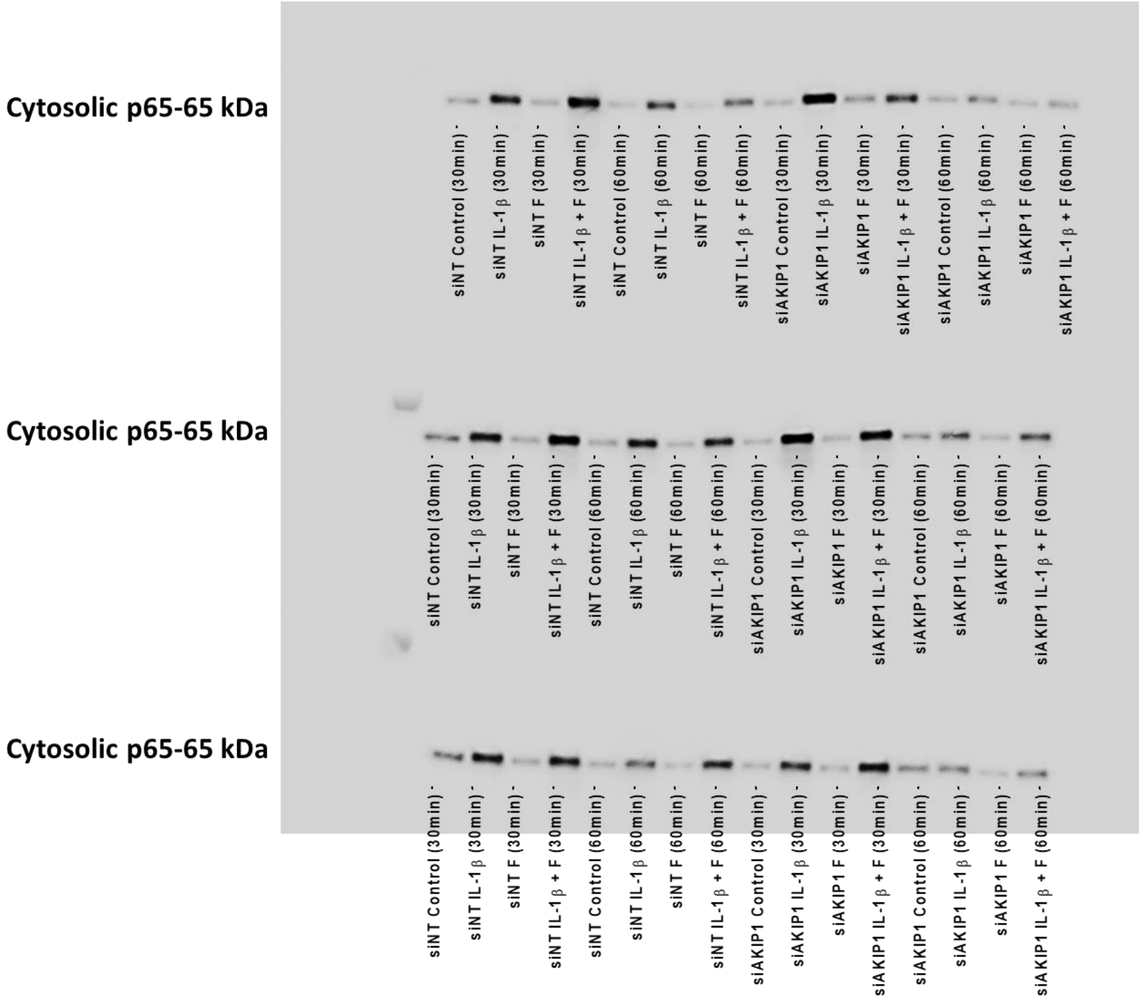

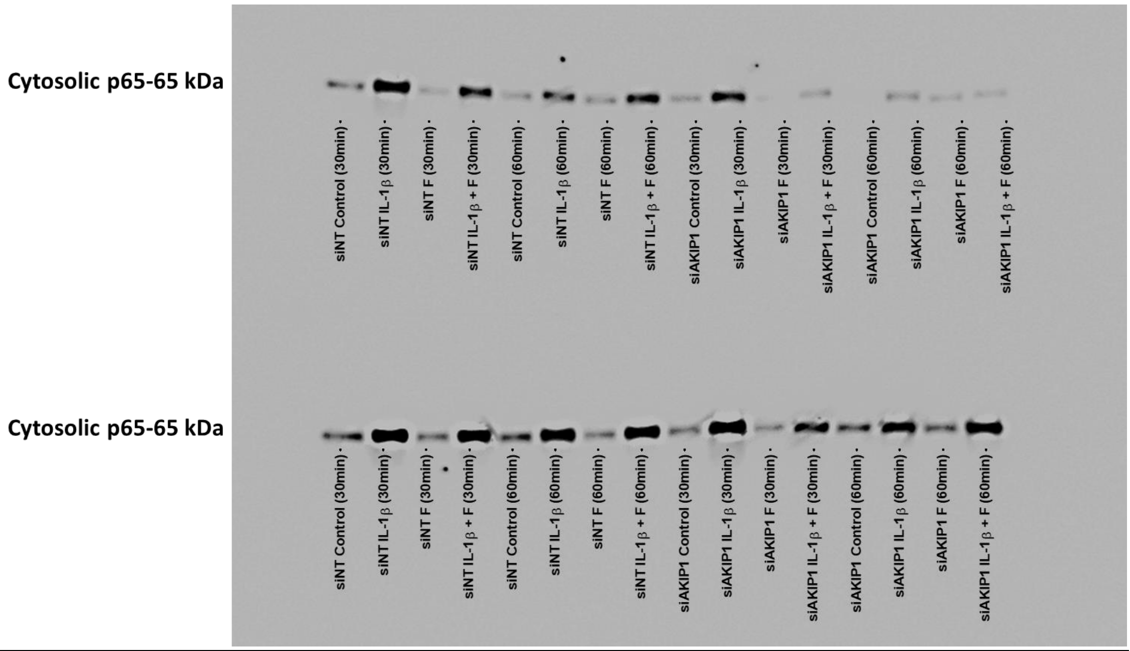

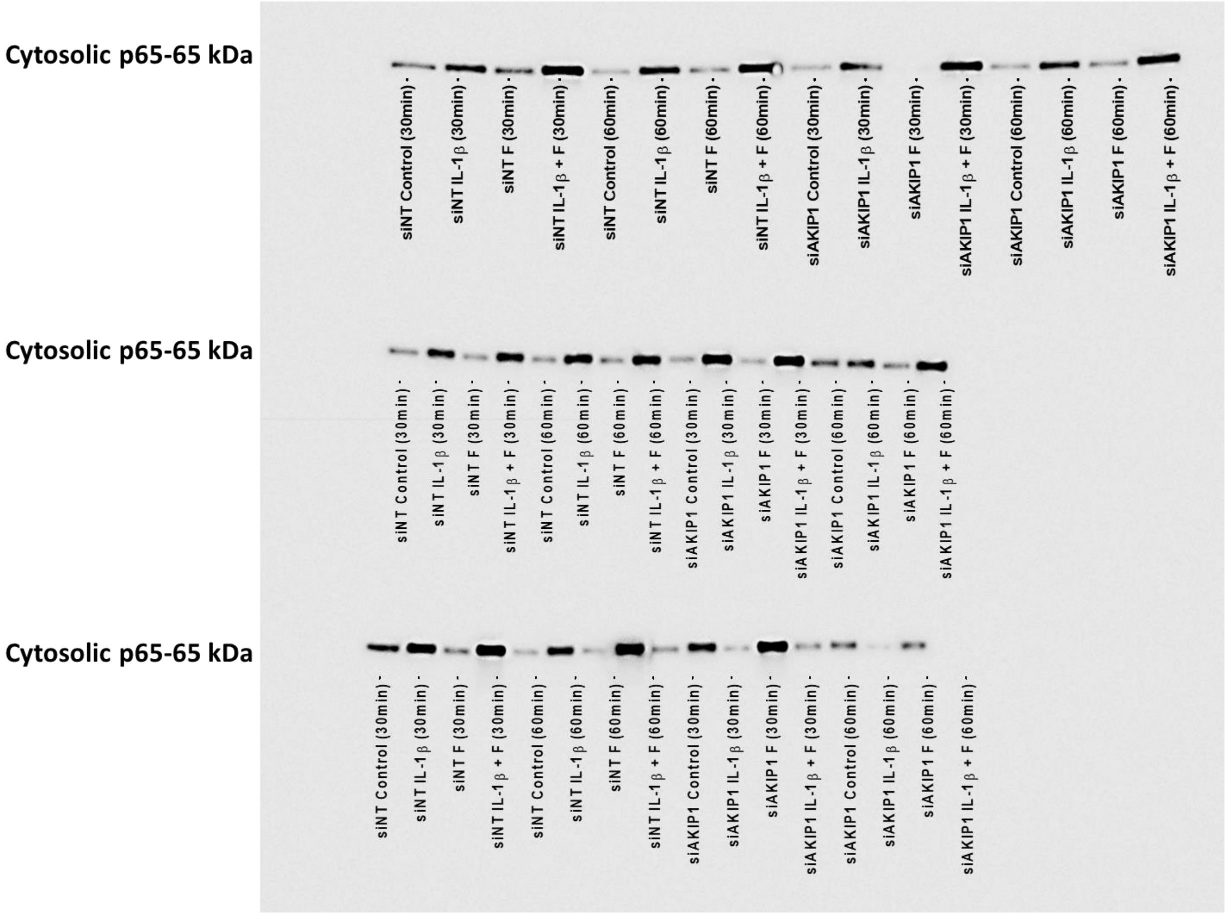
Cytosolic p65-65 kDa
siNT Control (30min)
siNT IL-1β (30min)
siNT F (30min)
siNT IL-1β + F (30min)
siNT Control (60min)
siNT IL-1β (60min)
siNT F (60min)
siNT IL-1β + F (60min)
siAKIP1 Control (30min)
siAKIP1 IL-1β (30min)
siAKIP1 F (30min)
siAKIP1 IL-1β + F (30min)
siAKIP1 Control (60min)
siAKIP1 IL-1β (60min)
siAKIP1 F (60min)
siAKIP1 IL-1β + F (60min)
Cytosolic p65-65 kDa
siNT Control (30min)
siNT IL-1β (30min)
siNT F (30min)
siNT IL-1β + F (30min)
siNT Control (60min)
siNT IL-1β (60min)
siNT F (60min)
siNT IL-1β + F (60min)
siAKIP1 Control (30min)
siAKIP1 IL-1β (30min)
siAKIP1 F (30min)
siAKIP1 IL-1β + F (30min)
siAKIP1 Control (60min)
siAKIP1 IL-1β (60min)
siAKIP1 F (60min)
siAKIP1 IL-1β + F (60min)
Cytosolic p65-65 kDa
siNT Control (30min)
siNT IL-1β (30min)
siNT F (30min)
siNT IL-1β + F (30min)
siNT Control (60min)
siNT IL-1β (60min)
siNT F (60min)
siNT IL-1β + F (60min)
siAKIP1 Control (30min)
siAKIP1 IL-1β (30min)
siAKIP1 F (30min)
siAKIP1 IL-1β + F (30min)
siAKIP1 Control (60min)
siAKIP1 IL-1β (60min)
siAKIP1 F (60min)
siAKIP1 IL-1β + F (60min)

Figure 2c – Cytoplasmic phospho c-jun

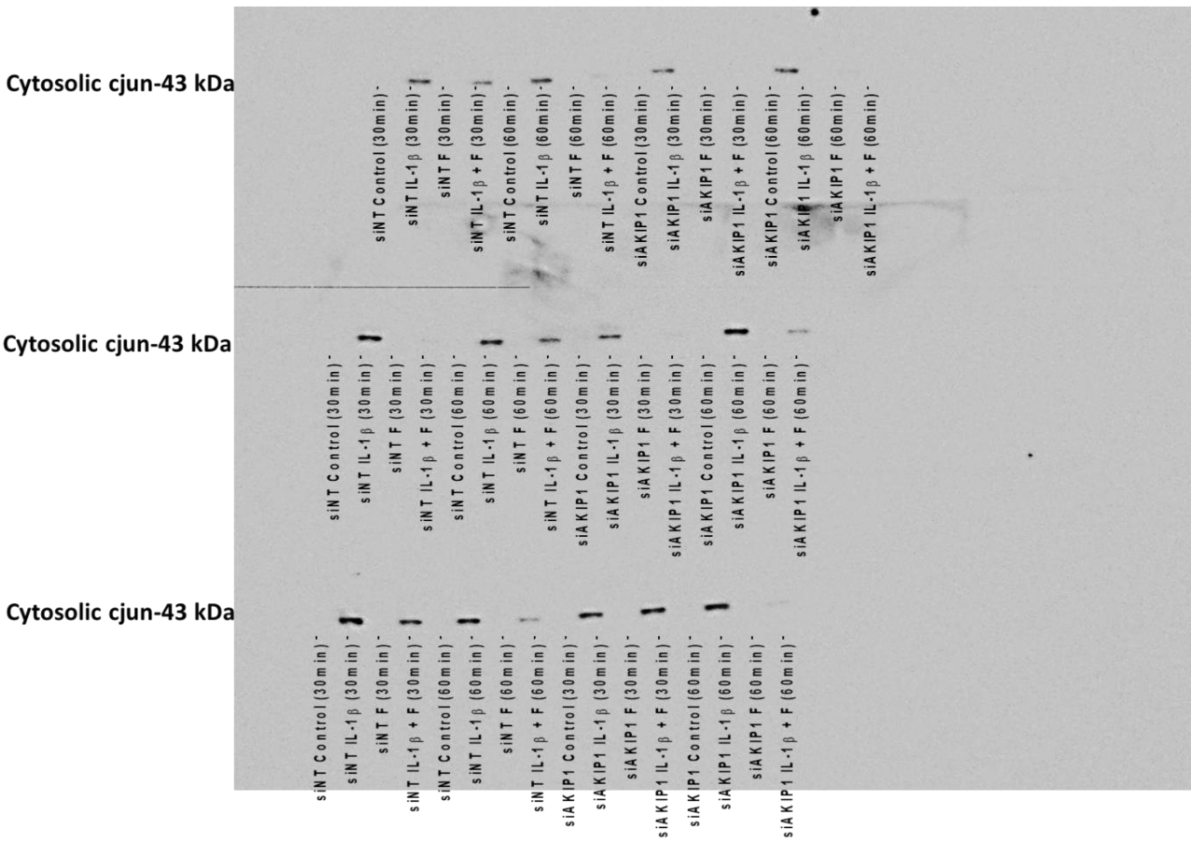

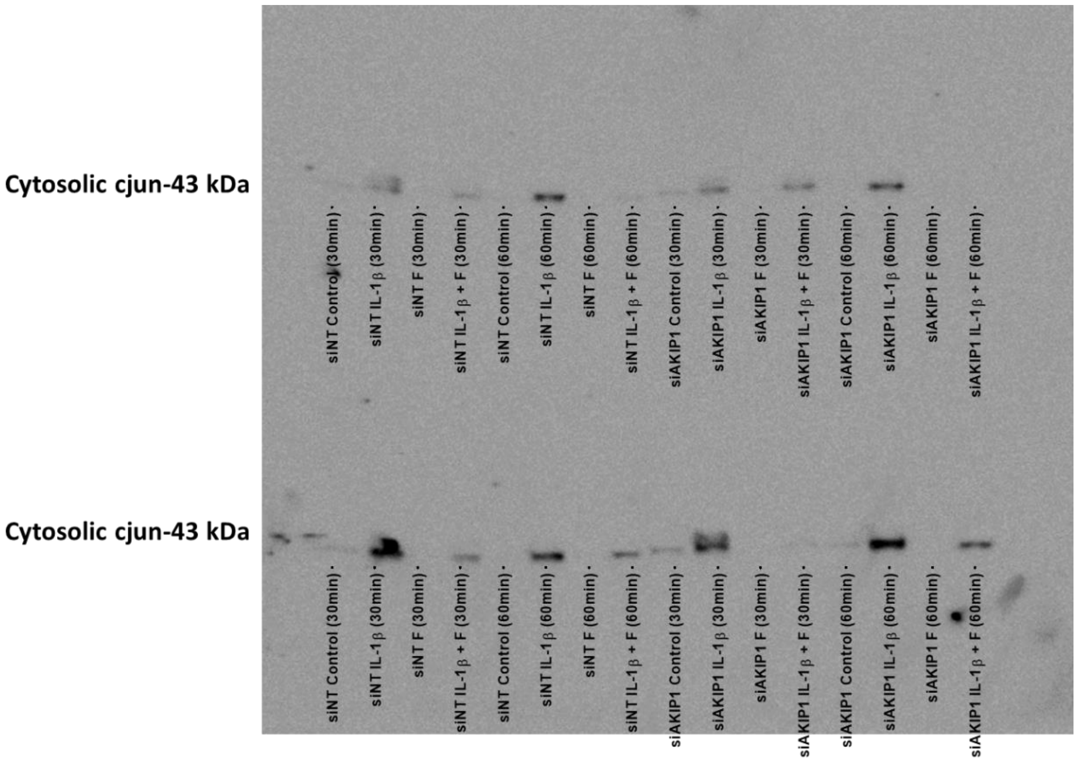

Cytosolic cjun-43 kDa

Cytosolic cjun-43 kDa

Cytosolic cjun-43 kDa

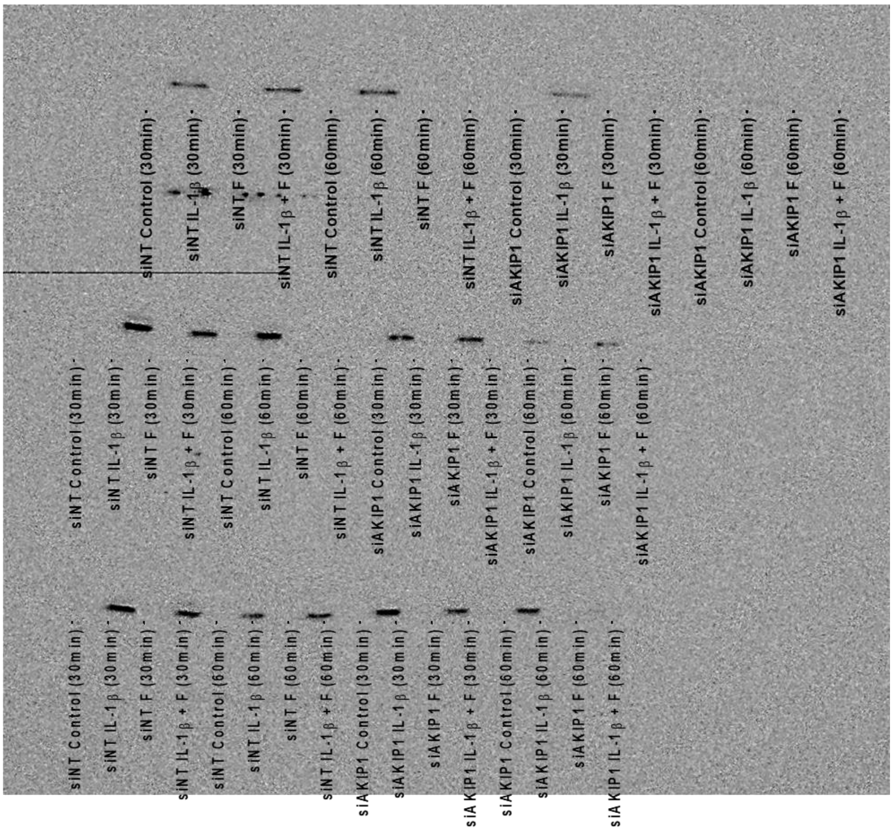

Figure 2a,c – Cytoplasmic Alpha Tubulin

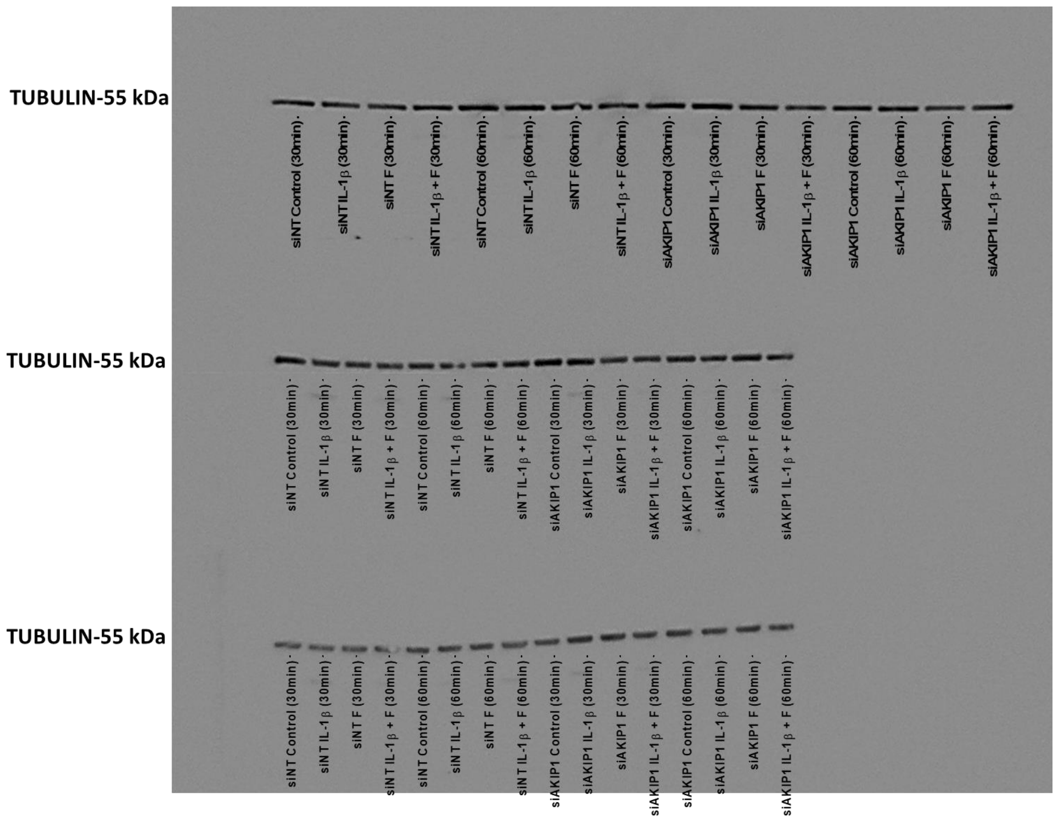

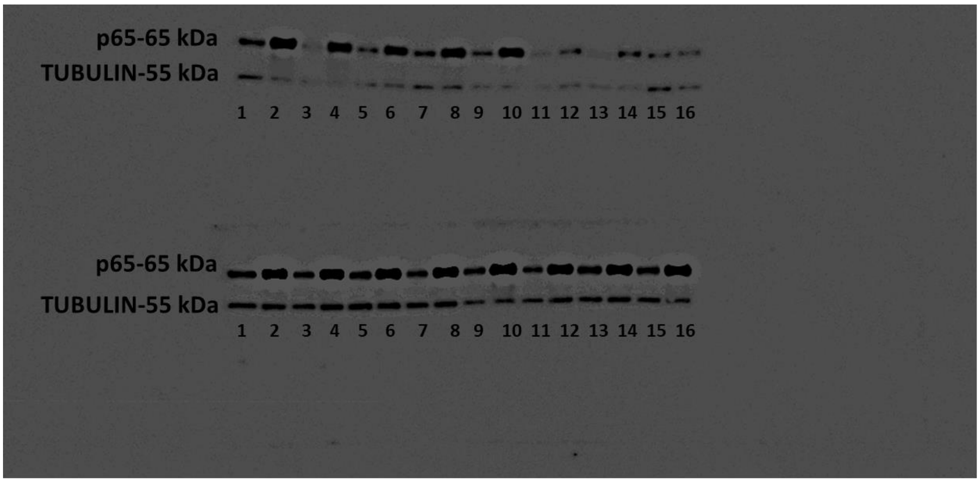

1. siNT Control (30 min)
2. siNT IL-1β (30 min)
3. siNT Forskolin (F) (30 min)
4. siNT F+IL-1β (30 min)
5. siNT Control (60 min)
6. siNT IL-1β (60 min)
7. siNT Forskolin (F) (360 min)
8. siNT F+IL-1β (60 min)
9. siAKIP1 Control (30 min)
10. siAKIP1 IL-1β (30 min)
11. siAKIP1 Forskolin (F) (30 min)
12. siAKIP1 F+IL-1β (30 min)
13. siAKIP1 Control (60 min)
14. siAKIP1 IL-1β (60 min)
15. siAKIP1 Forskolin (F) (60 min)
16. siAKIP1 F+IL-1β (60 min)

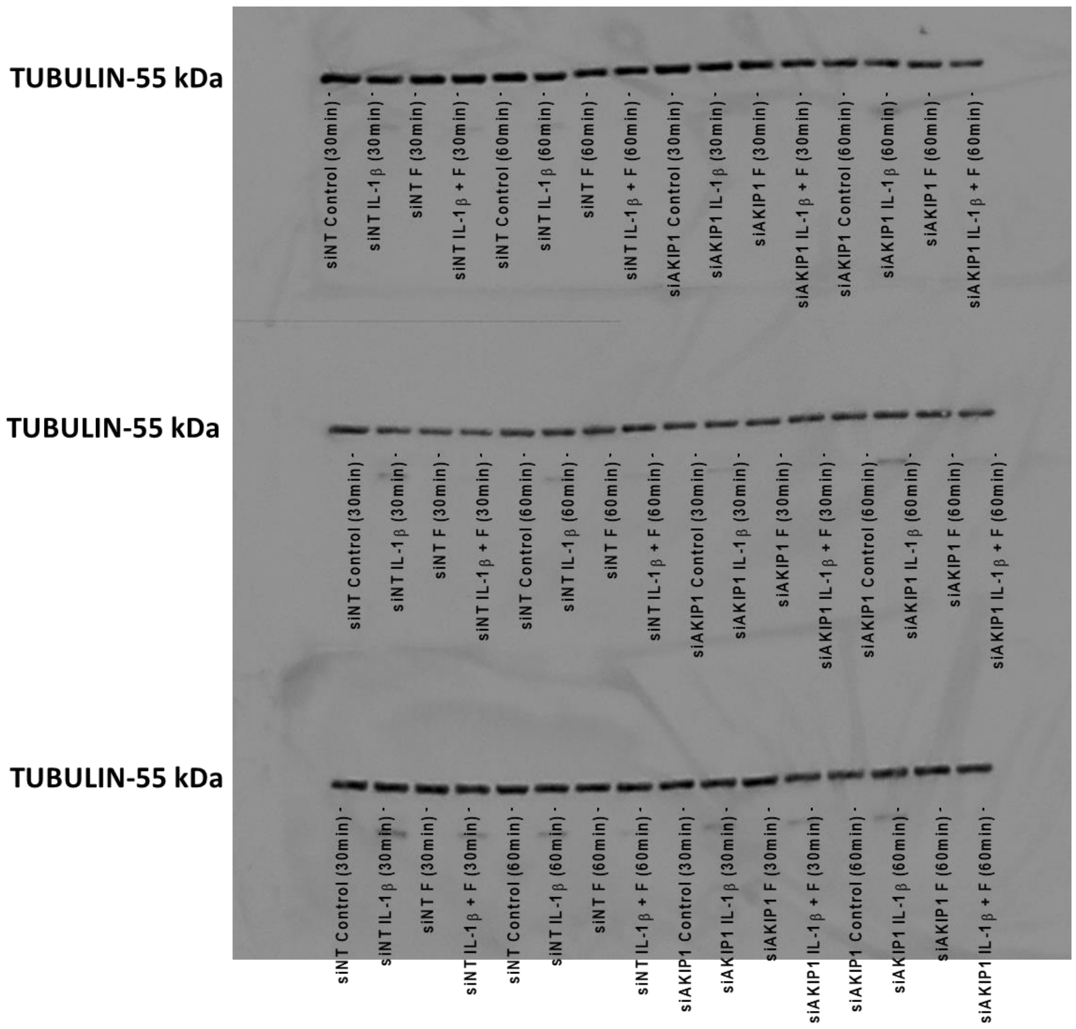
TUBULIN-55 kDa
siNT Control (30min)
siNT IL-1β (30min)
siNT F (30min)
siNT IL-1β + F (30min)
siNT Control (60min)
siNT IL-1β (60min)
siNT F (60min)
siNT IL-1β + F (60min)
siAKIP1 Control (30min)
siAKIP1 IL-1β (30min)
siAKIP1 F (30min)
siAKIP1 IL-1β + F (30min)
siAKIP1 Control (60min)
siAKIP1 IL-1β (60min)
siAKIP1 F (60min)
siAKIP1 IL-1β + F (60min)
TUBULIN-55 kDa
siNT Control (30min)
siNT IL-1β (30min)
siNT F (30min)
siNT IL-1β + F (30min)
siNT Control (60min)
siNT IL-1β (60min)
siNT F (60min)
siNT IL-1β + F (60min)
siAKIP1 Control (30min)
siAKIP1 IL-1β (30min)
siAKIP1 F (30min)
siAKIP1 IL-1β + F (30min)
siAKIP1 Control (60min)
siAKIP1 IL-1β (60min)
siAKIP1 F (60min)
siAKIP1 IL-1β + F (60min)
TUBULIN-55 kDa
siNT Control (30min)
siNT IL-1β (30min)
siNT F (30min)
siNT IL-1β + F (30min)
siNT Control (60min)
siNT IL-1β (60min)
siNT F (60min)
siNT IL-1β + F (60min)
siAKIP1 Control (30min)
siAKIP1 IL-1β (30min)
siAKIP1 F (30min)
siAKIP1 IL-1β + F (30min)
siAKIP1 Control (60min)
siAKIP1 IL-1β (60min)
siAKIP1 F (60min)
siAKIP1 IL-1β + F (60min)

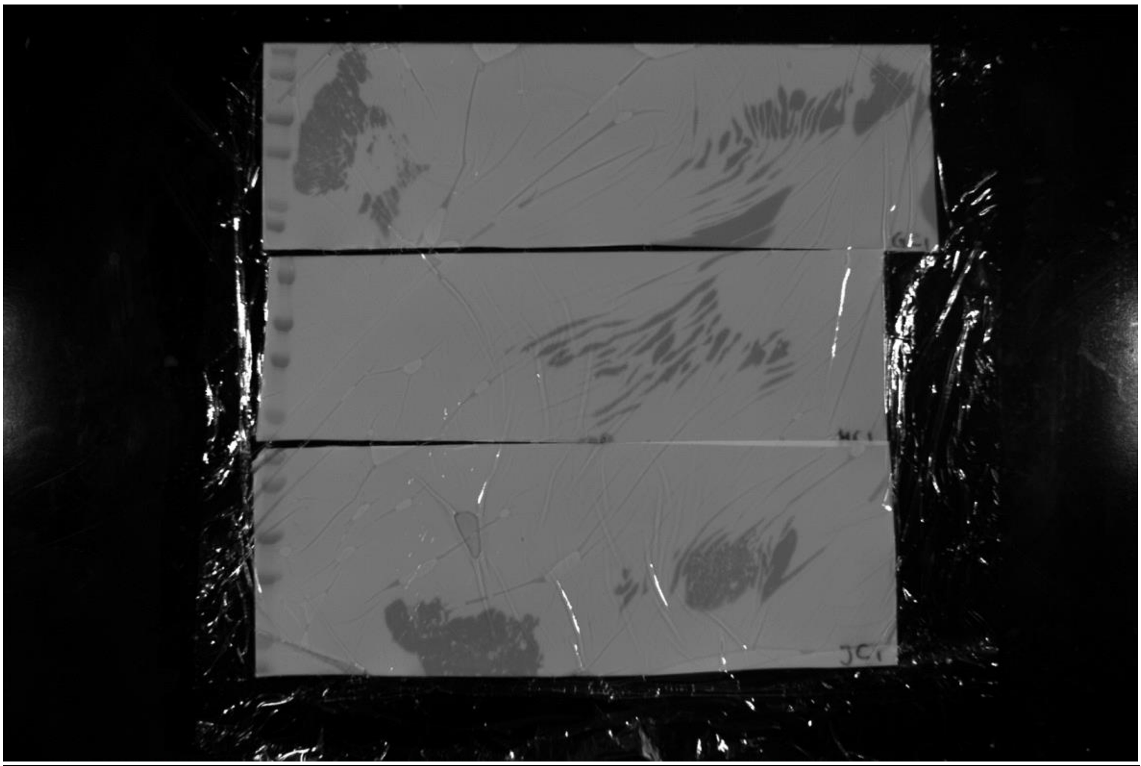

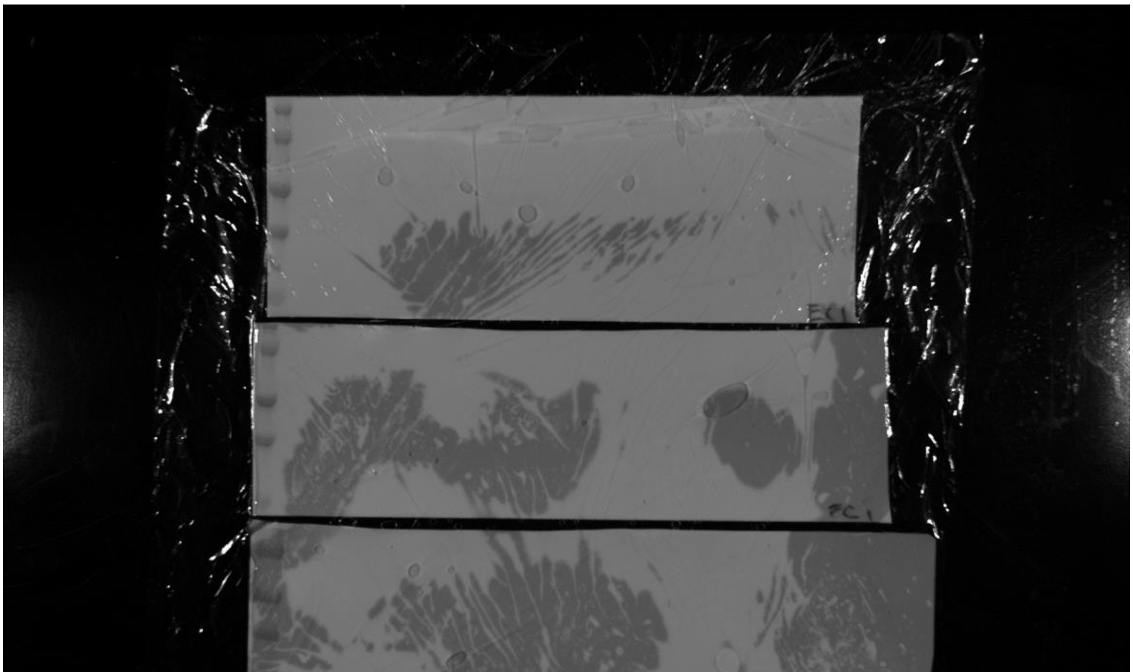

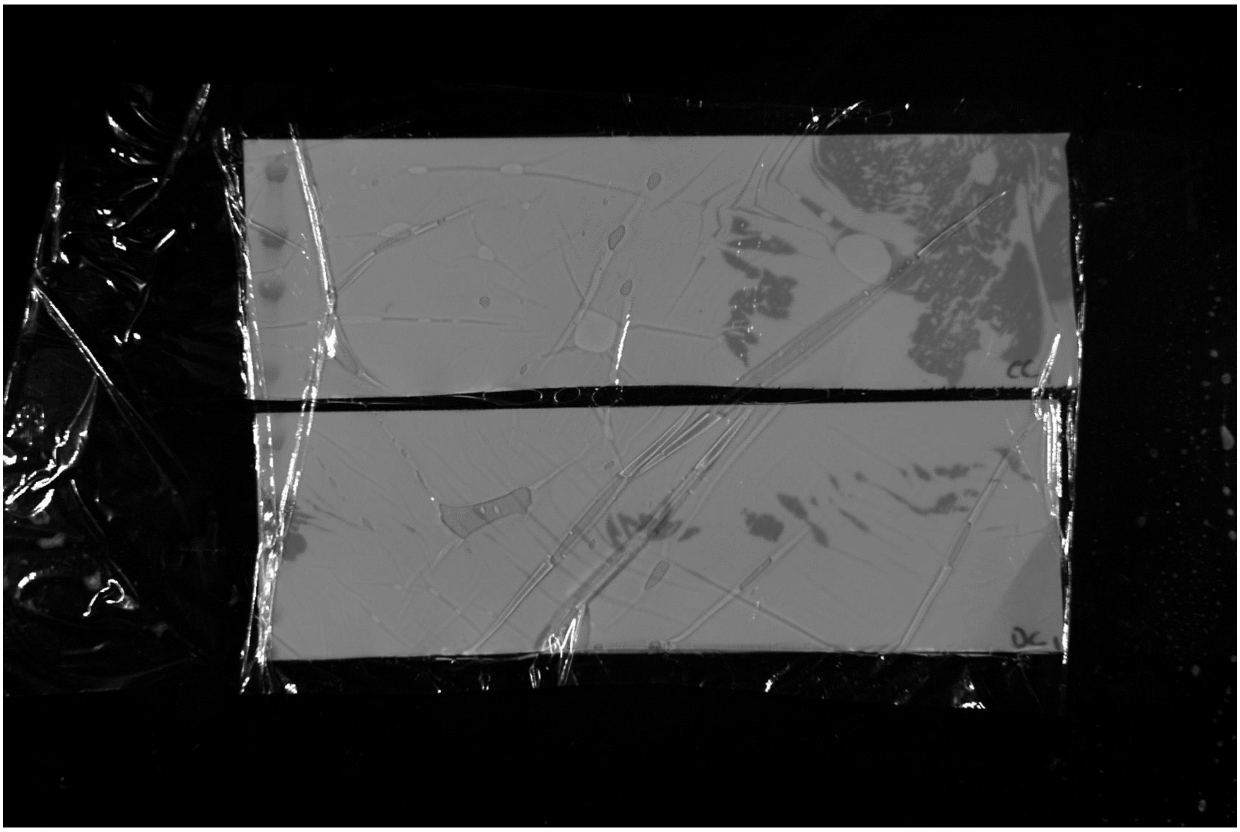

Figure 2b – Nuclear phospho p65

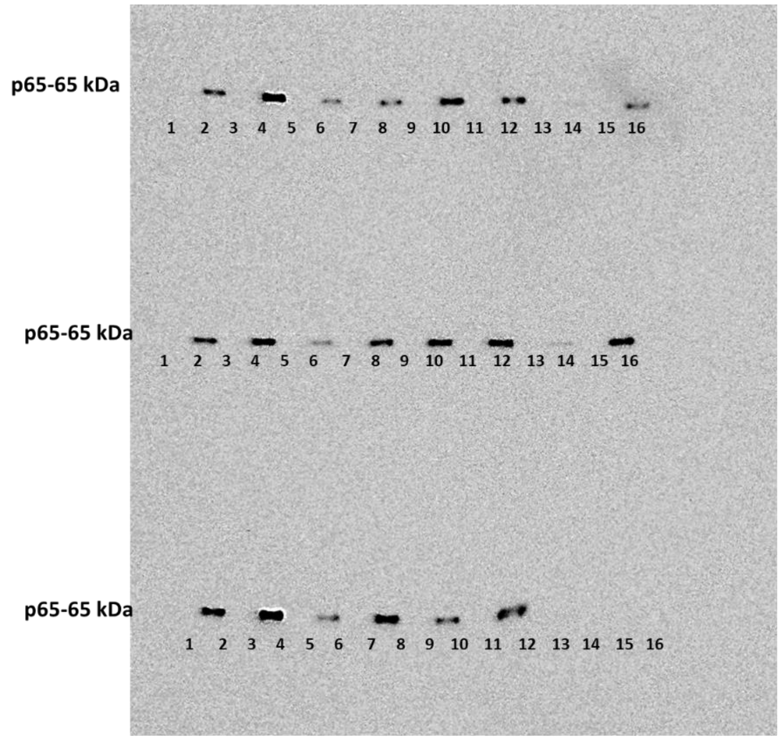

1. siNT Control (30 min)
2. siNT IL-1β (30 min)
3. siNT Forskolin (F) (30 min)
4. siNT F+IL-1β (30 min)
5. siNT Control (60 min)
6. siNT IL-1β (60 min)
7. siNT Forskolin (F) (360 min)
8. siNT F+IL-1β (60 min)
9. siAKIP1 Control (30 min)
10. siAKIP1 IL-1β (30 min)
11. siAKIP1 Forskolin (F) (30 min)
12. siAKIP1 F+IL-1β (30 min)
13. siAKIP1 Control (60 min)
14. siAKIP1 IL-1β (60 min)
15. siAKIP1 Forskolin (F) (60 min)
16. siAKIP1 F+IL-1β (60 min)

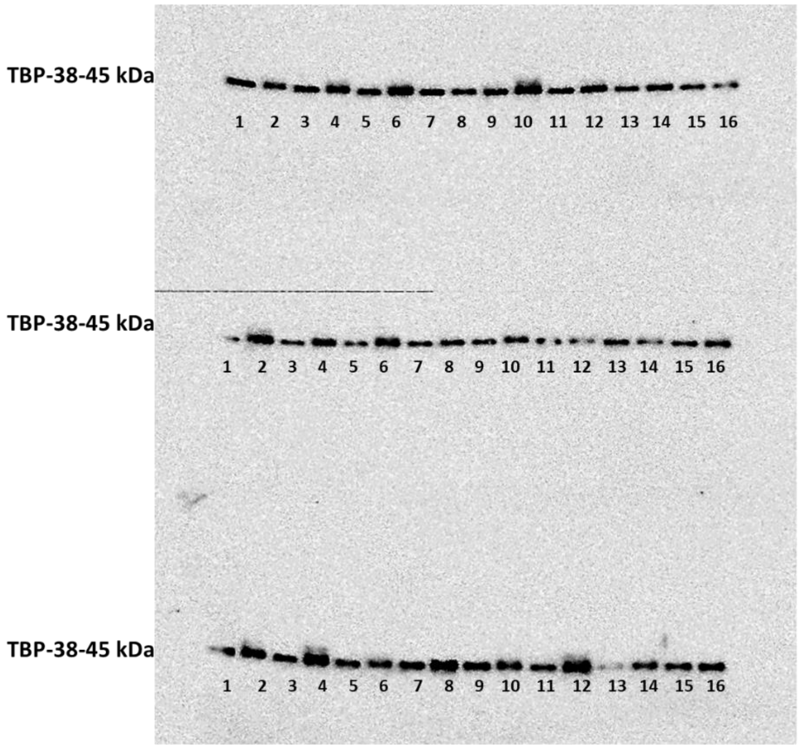

1. siNT Control (30 min)
2. siNT IL-1β (30 min)
3. siNT Forskolin (F) (30 min)
4. siNT F+IL-1β (30 min)
5. siNT Control (60 min)
6. siNT IL-1β (60 min)
7. siNT Forskolin (F) (360 min)
8. siNT F+IL-1β (60 min)
9. siAKIP1 Control (30 min)
10. siAKIP1 IL-1β (30 min)
11. siAKIP1 Forskolin (F) (30 min)
12. siAKIP1 F+IL-1β (30 min)
13. siAKIP1 Control (60 min)
14. siAKIP1 IL-1β (60 min)
15. siAKIP1 Forskolin (F) (60 min)
16. siAKIP1 F+IL-1β (60 min)

---

p65-65 kDa

p65-65 kDa

p65-65 kDa

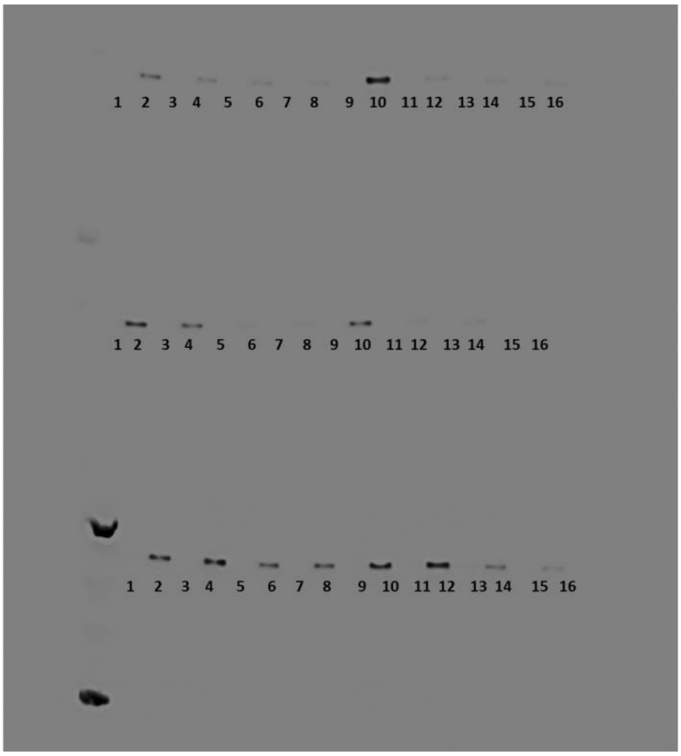

1. siNT Control (30 min)
2. siNT IL-1β (30 min)
3. siNT Forskolin (F) (30 min)
4. siNT F+IL-1β (30 min)
5. siNT Control (60 min)
6. siNT IL-1β (60 min)
7. siNT Forskolin (F) (360 min)
8. siNT F+IL-1β (60 min)
9. siAKIP1 Control (30 min)
10. siAKIP1 IL-1β (30 min)
11. siAKIP1 Forskolin (F) (30 min)
12. siAKIP1 F+IL-1β (30 min)
13. siAKIP1 Control (60 min)
14. siAKIP1 IL-1β (60 min)
15. siAKIP1 Forskolin (F) (60 min)
16. siAKIP1 F+IL-1β (60 min)

TBP-38-45 kDa

TBP-38-45 kDa

TBP-38-45 kDa

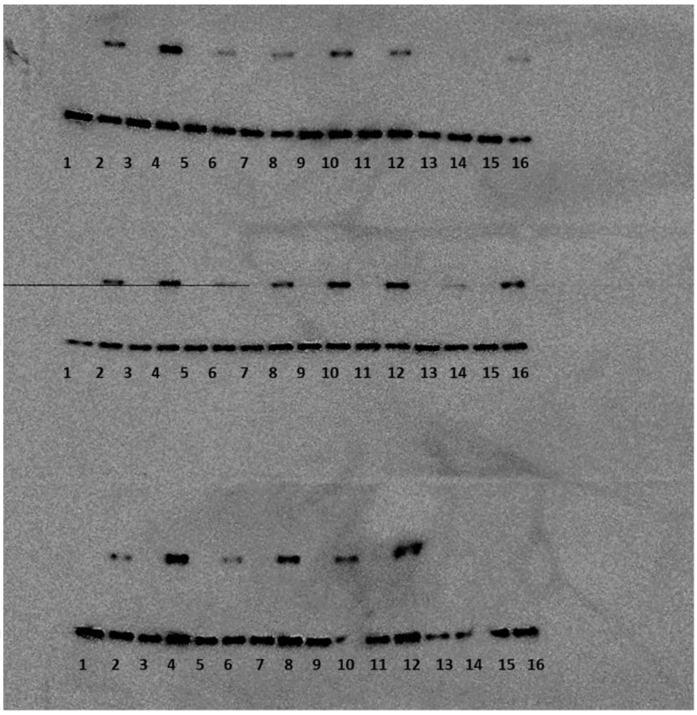

1. siNT Control (30 min)
2. siNT IL-1β (30 min)
3. siNT Forskolin (F) (30 min)
4. siNT F+IL-1β (30 min)
5. siNT Control (60 min)
6. siNT IL-1β (60 min)
7. siNT Forskolin (F) (360 min)
8. siNT F+IL-1β (60 min)
9. siAKIP1 Control (30 min)
10. siAKIP1 IL-1β (30 min)
11. siAKIP1 Forskolin (F) (30 min)
12. siAKIP1 F+IL-1β (30 min)
13. siAKIP1 Control (60 min)
14. siAKIP1 IL-1β (60 min)
15. siAKIP1 Forskolin (F) (60 min)
16. siAKIP1 F+IL-1β (60 min)

p65-65 kDa

p65-65 kDa

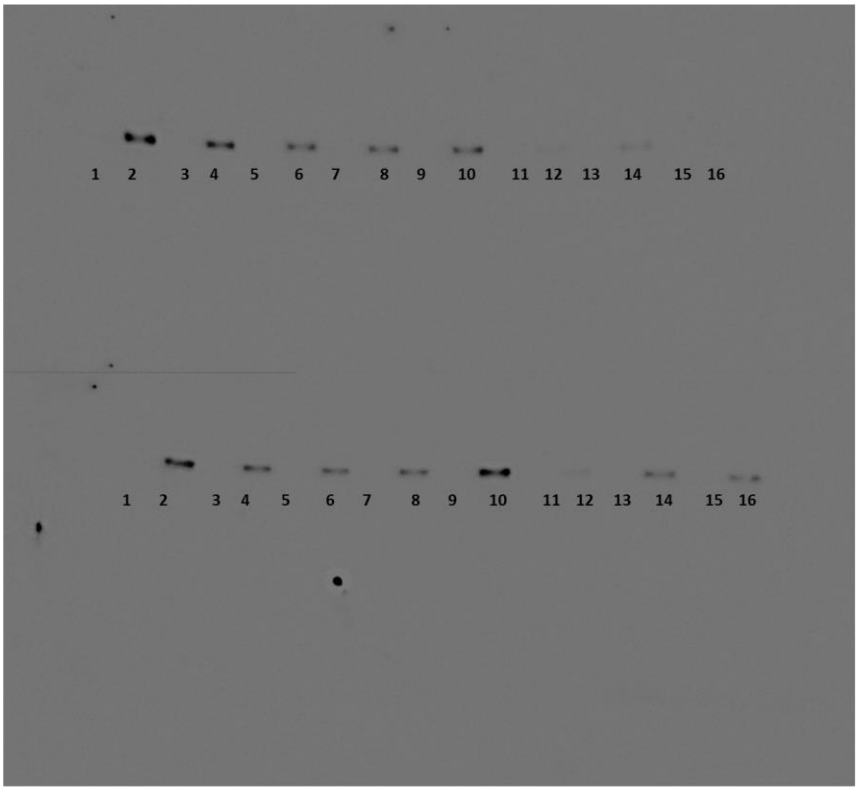

1. siNT Control (30 min)
2. siNT IL-1β (30 min)
3. siNT Forskolin (F) (30 min)
4. siNT F+IL-1β (30 min)
5. siNT Control (60 min)
6. siNT IL-1β (60 min)
7. siNT Forskolin (F) (360 min)
8. siNT F+IL-1β (60 min)
9. siAKIP1 Control (30 min)
10. siAKIP1 IL-1β (30 min)
11. siAKIP1 Forskolin (F) (30 min)
12. siAKIP1 F+IL-1β (30 min)
13. siAKIP1 Control (60 min)
14. siAKIP1 IL-1β (60 min)
15. siAKIP1 Forskolin (F) (60 min)
16. siAKIP1 F+IL-1β (60 min)

TBP-38-45 kDa

TBP-38-45 kDa

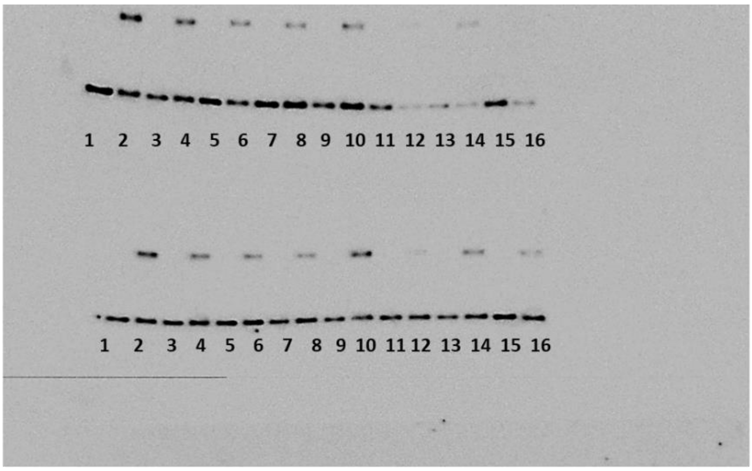

1. siNT Control (30 min)
2. siNT IL-1β (30 min)
3. siNT Forskolin (F) (30 min)
4. siNT F+IL-1β (30 min)
5. siNT Control (60 min)
6. siNT IL-1β (60 min)
7. siNT Forskolin (F) (360 min)
8. siNT F+IL-1β (60 min)
9. siAKIP1 Control (30 min)
10. siAKIP1 IL-1β (30 min)
11. siAKIP1 Forskolin (F) (30 min)
12. siAKIP1 F+IL-1β (30 min)
13. siAKIP1 Control (60 min)
14. siAKIP1 IL-1β (60 min)
15. siAKIP1 Forskolin (F) (60 min)
16. siAKIP1 F+IL-1β (60 min)

Figure 2d – Nuclear phospho cjun

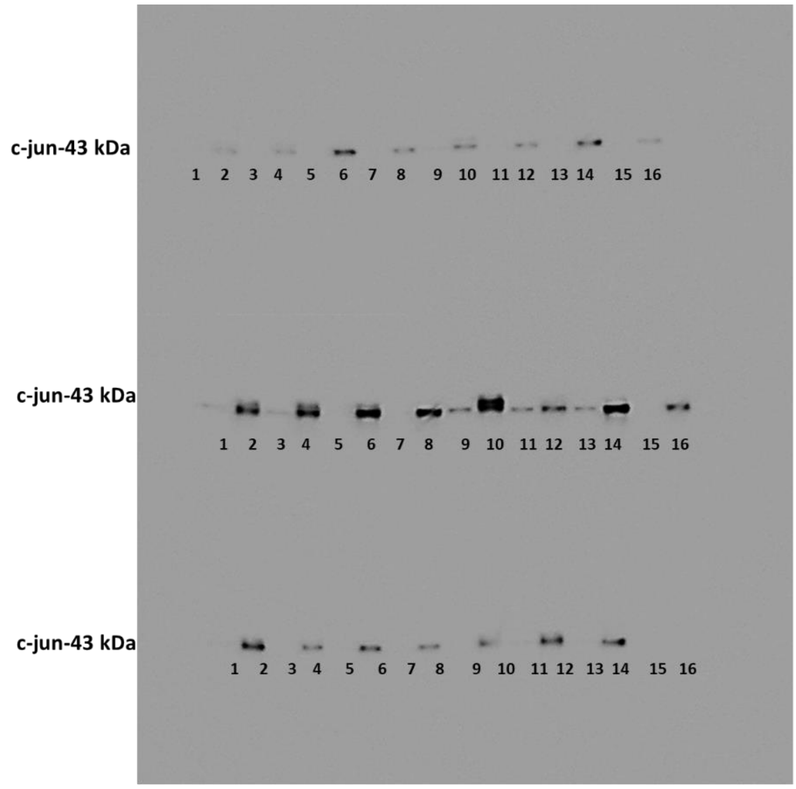

1. siNT Control (30 min)
2. siNT IL-1β (30 min)
3. siNT Forskolin (F) (30 min)
4. siNT F+IL-1β (30 min)
5. siNT Control (60 min)
6. siNT IL-1β (60 min)
7. siNT Forskolin (F) (360 min)
8. siNT F+IL-1β (60 min)
9. siAKIP1 Control (30 min)
10. siAKIP1 IL-1β (30 min)
11. siAKIP1 Forskolin (F) (30 min)
12. siAKIP1 F+IL-1β (30 min)
13. siAKIP1 Control (60 min)
14. siAKIP1 IL-1β (60 min)
15. siAKIP1 Forskolin (F) (60 min)
16. siAKIP1 F+IL-1β (60 min)

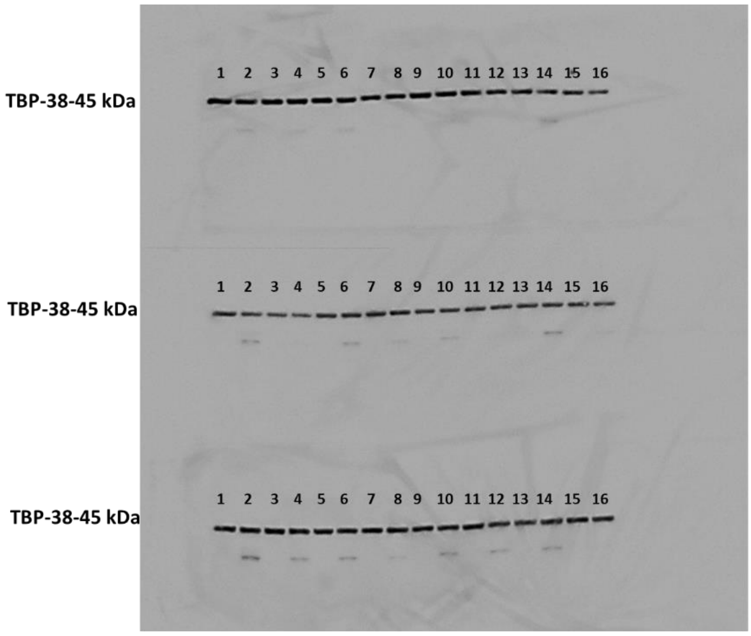

1. siNT Control (30 min)
2. siNT IL-1β (30 min)
3. siNT Forskolin (F) (30 min)
4. siNT F+IL-1β (30 min)
5. siNT Control (60 min)
6. siNT IL-1β (60 min)
7. siNT Forskolin (F) (360 min)
8. siNT F+IL-1β (60 min)
9. siAKIP1 Control (30 min)
10. siAKIP1 IL-1β (30 min)
11. siAKIP1 Forskolin (F) (30 min)
12. siAKIP1 F+IL-1β (30 min)
13. siAKIP1 Control (60 min)
14. siAKIP1 IL-1β (60 min)
15. siAKIP1 Forskolin (F) (60 min)
16. siAKIP1 F+IL-1β (60 min)

---

c-jun-43 kDa

c-jun-43 kDa

c-jun-43 kDa

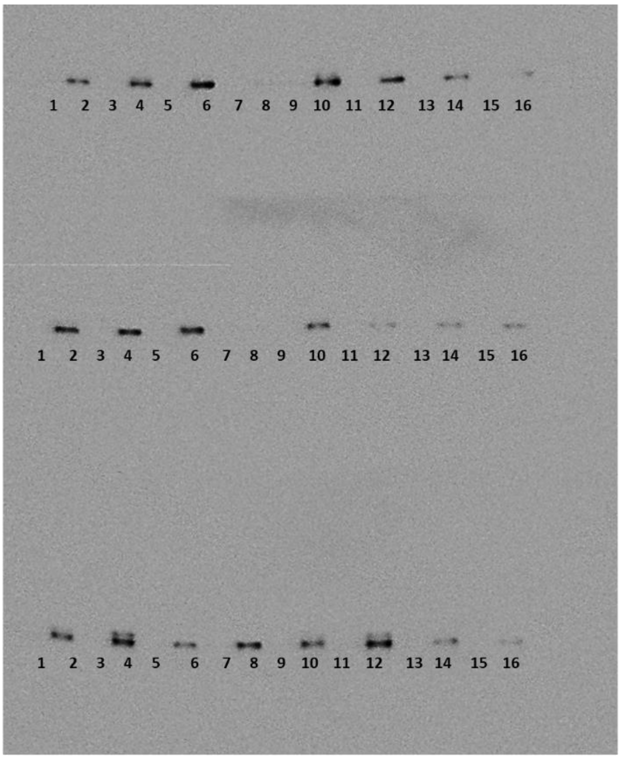

1. siNT Control (30 min)
2. siNT IL-1β (30 min)
3. siNT Forskolin (F) (30 min)
4. siNT F+IL-1β (30 min)
5. siNT Control (60 min)
6. siNT IL-1β (60 min)
7. siNT Forskolin (F) (360 min)
8. siNT F+IL-1β (60 min)
9. siAKIP1 Control (30 min)
10. siAKIP1 IL-1β (30 min)
11. siAKIP1 Forskolin (F) (30 min)
12. siAKIP1 F+IL-1β (30 min)
13. siAKIP1 Control (60 min)
14. siAKIP1 IL-1β (60 min)
15. siAKIP1 Forskolin (F) (60 min)
16. siAKIP1 F+IL-1β (60 min)

TBP-38-45 kDa

TBP-38-45 kDa

TBP-38-45 kDa

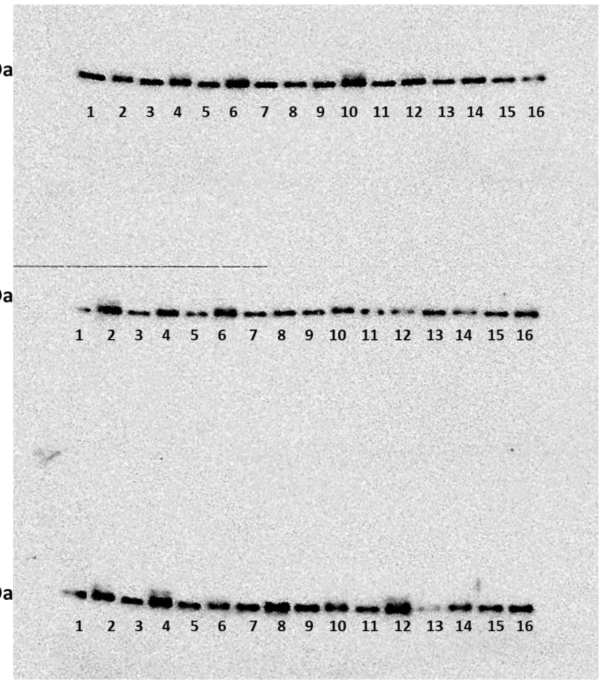

1. siNT Control (30 min)
2. siNT IL-1β (30 min)
3. siNT Forskolin (F) (30 min)
4. siNT F+IL-1β (30 min)
5. siNT Control (60 min)
6. siNT IL-1β (60 min)
7. siNT Forskolin (F) (360 min)
8. siNT F+IL-1β (60 min)
9. siAKIP1 Control (30 min)
10. siAKIP1 IL-1β (30 min)
11. siAKIP1 Forskolin (F) (30 min)
12. siAKIP1 F+IL-1β (30 min)
13. siAKIP1 Control (60 min)
14. siAKIP1 IL-1β (60 min)
15. siAKIP1 Forskolin (F) (60 min)
16. siAKIP1 F+IL-1β (60 min)

---

c-jun-43 kDa

c-jun-43 kDa

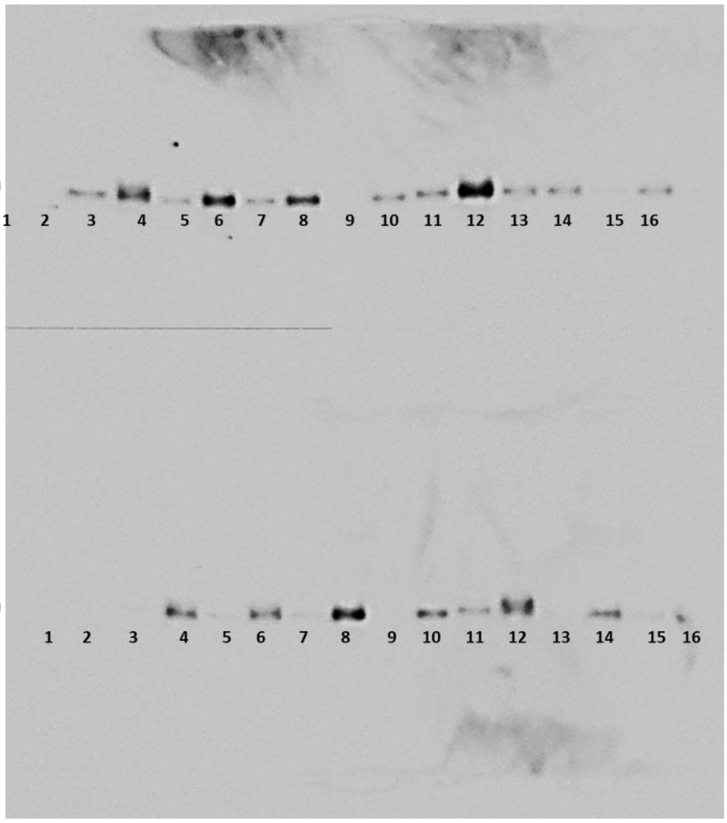

1. siNT Control (30 min)
2. siNT IL-1β (30 min)
3. siNT Forskolin (F) (30 min)
4. siNT F+IL-1β (30 min)
5. siNT Control (60 min)
6. siNT IL-1β (60 min)
7. siNT Forskolin (F) (360 min)
8. siNT F+IL-1β (60 min)
9. siAKIP1 Control (30 min)
10. siAKIP1 IL-1β (30 min)
11. siAKIP1 Forskolin (F) (30 min)
12. siAKIP1 F+IL-1β (30 min)
13. siAKIP1 Control (60 min)
14. siAKIP1 IL-1β (60 min)
15. siAKIP1 Forskolin (F) (60 min)
16. siAKIP1 F+IL-1β (60 min)

TBP-38-45 kDa

TBP-38-45 kDa

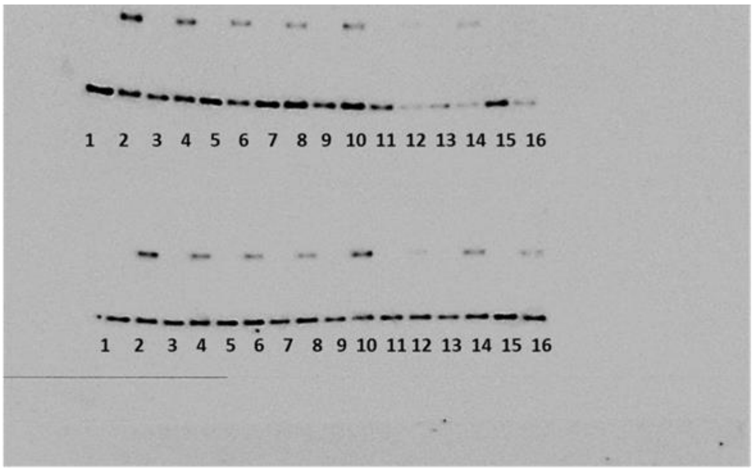

1. siNT Control (30 min)
2. siNT IL-1β (30 min)
3. siNT Forskolin (F) (30 min)
4. siNT F+IL-1β (30 min)
5. siNT Control (60 min)
6. siNT IL-1β (60 min)
7. siNT Forskolin (F) (360 min)
8. siNT F+IL-1β (60 min)
9. siAKIP1 Control (30 min)
10. siAKIP1 IL-1β (30 min)
11. siAKIP1 Forskolin (F) (30 min)
12. siAKIP1 F+IL-1β (30 min)
13. siAKIP1 Control (60 min)
14. siAKIP1 IL-1β (60 min)
15. siAKIP1 Forskolin (F) (60 min)
16. siAKIP1 F+IL-1β (60 min)

Figure 4b

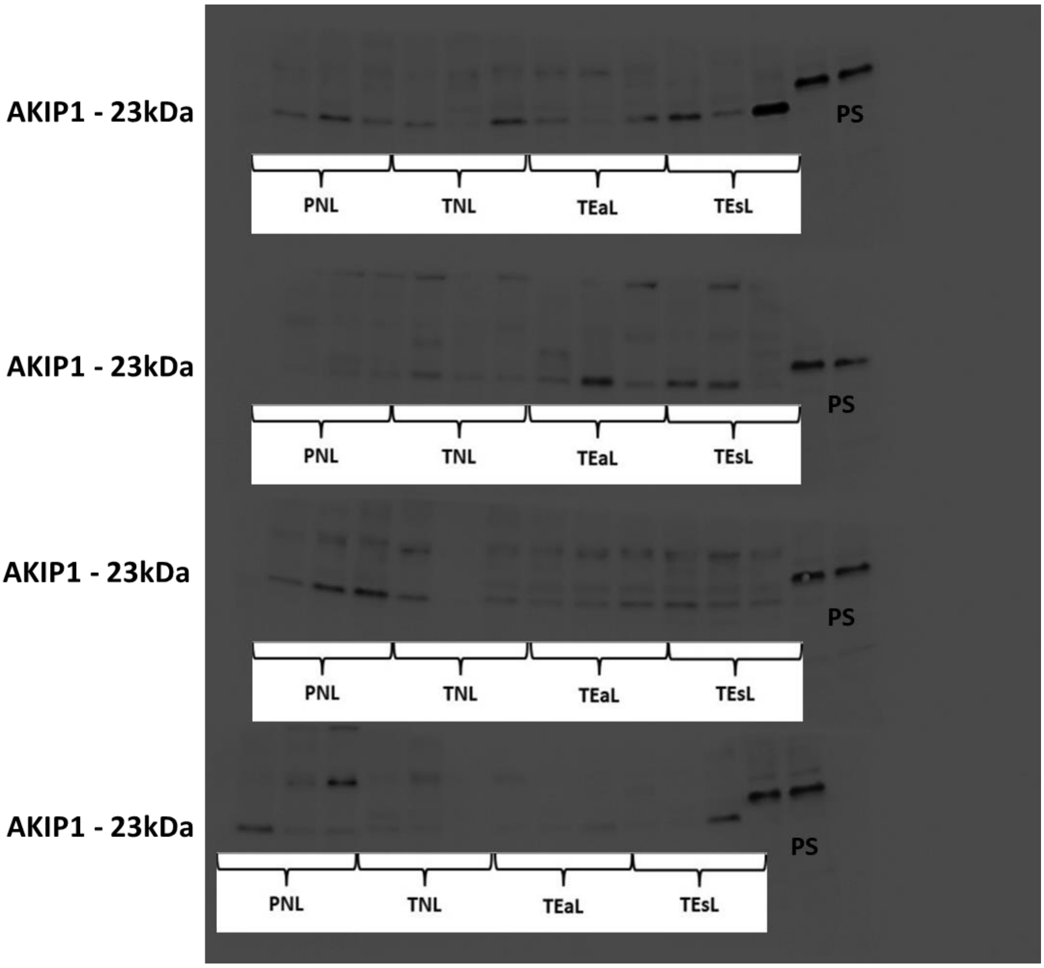

PNL – Preterm non labour

TNL – Term non labour

TEaL – Term early labour

TEsL – Term established labour

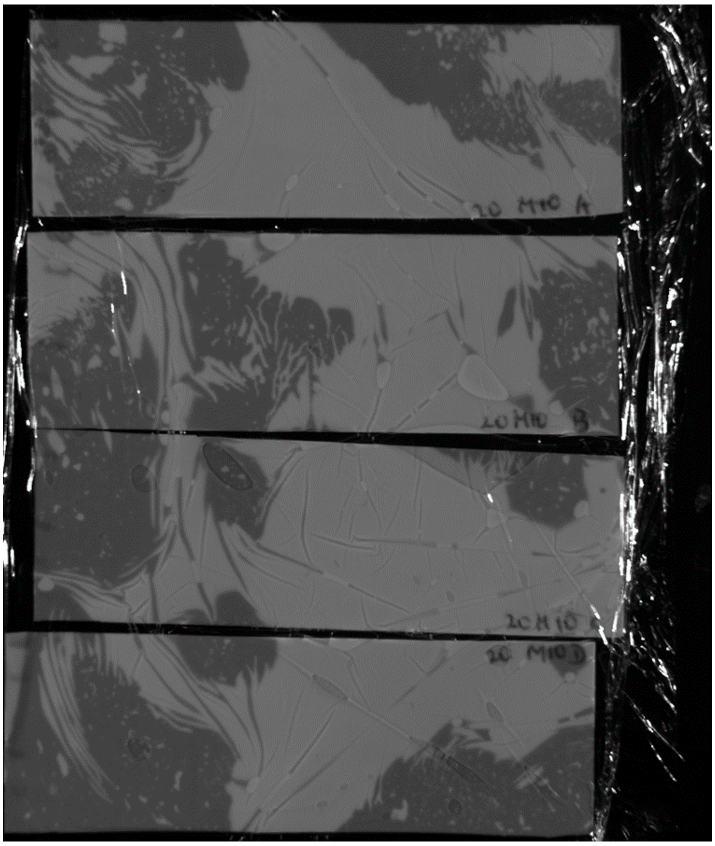

GAPDH-38 kDa

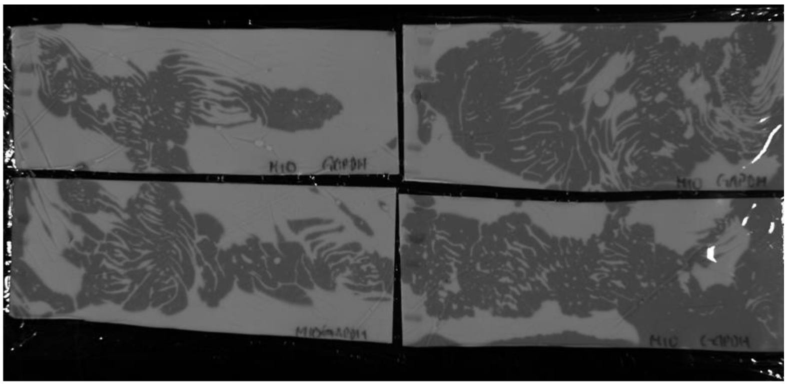

GAPDH-38 kDa

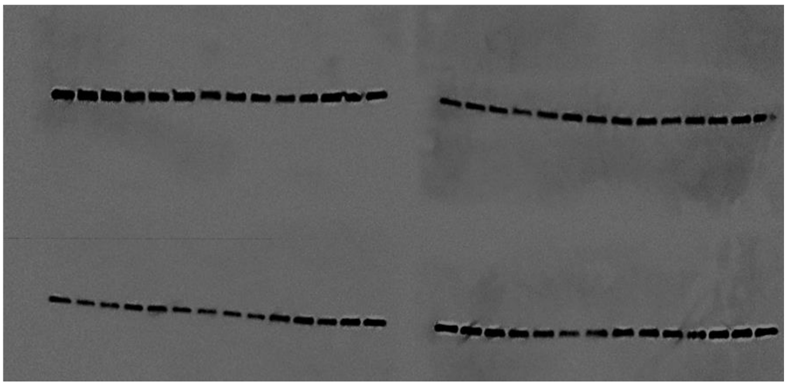

Figure 4d

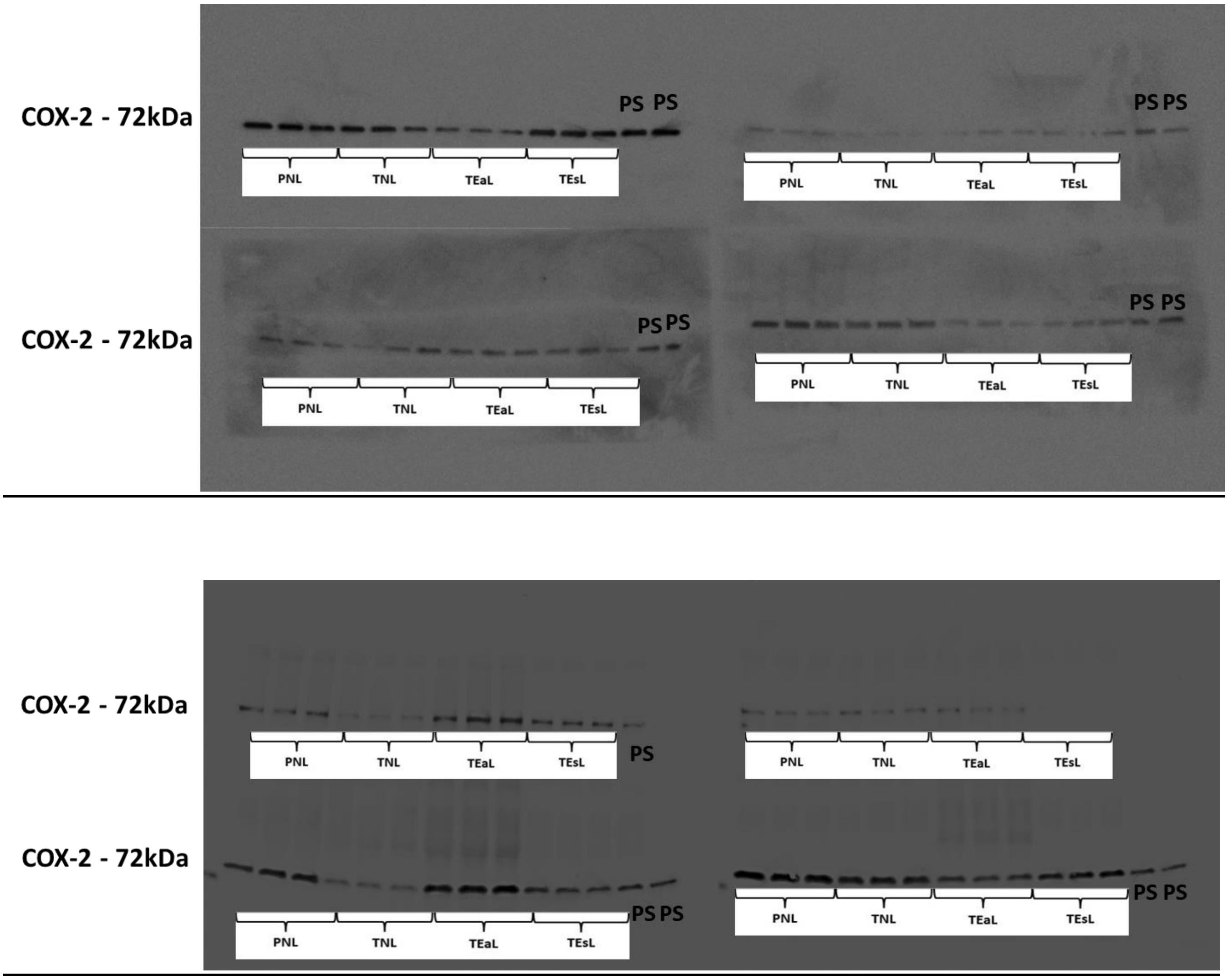

PNL – Preterm non labour

TNL – Term non labour

TEaL – Term early labour

TEsL – Term established labour

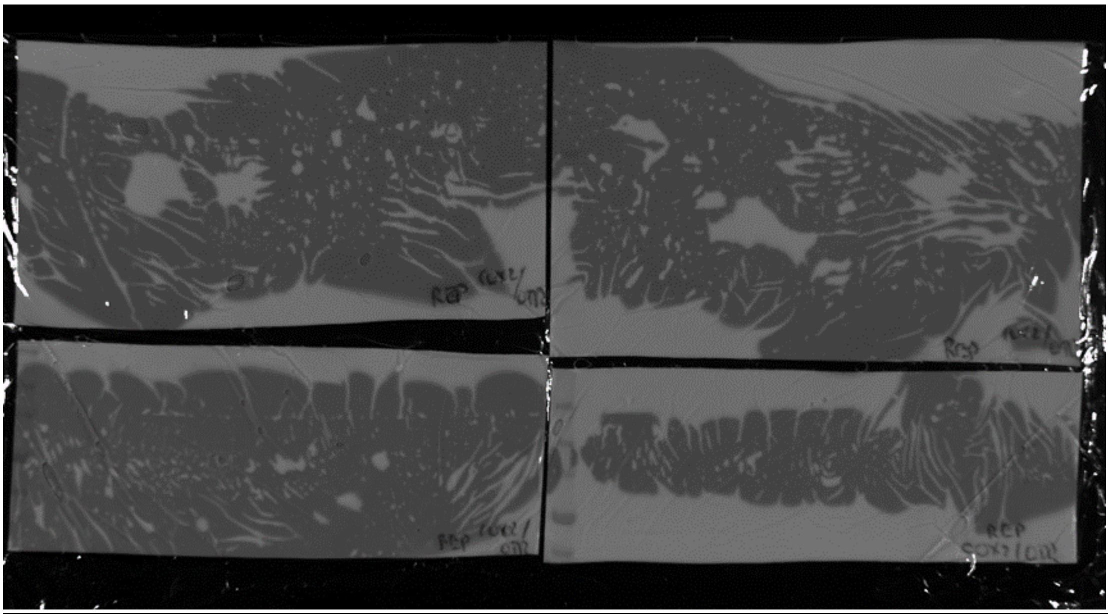

COX-2 - 72kDa

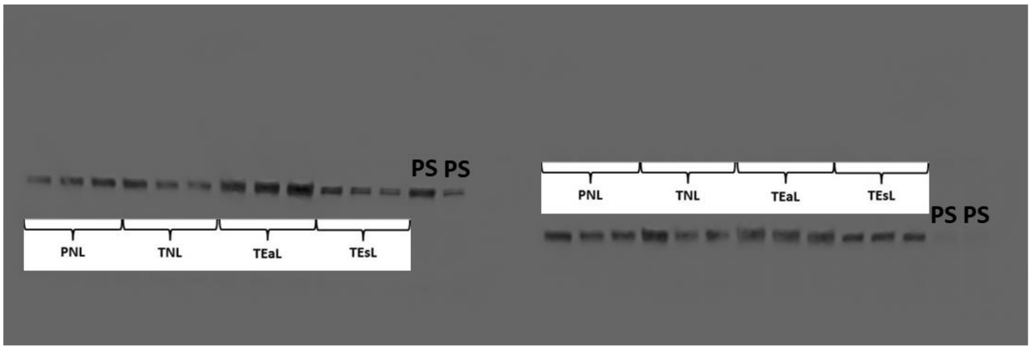

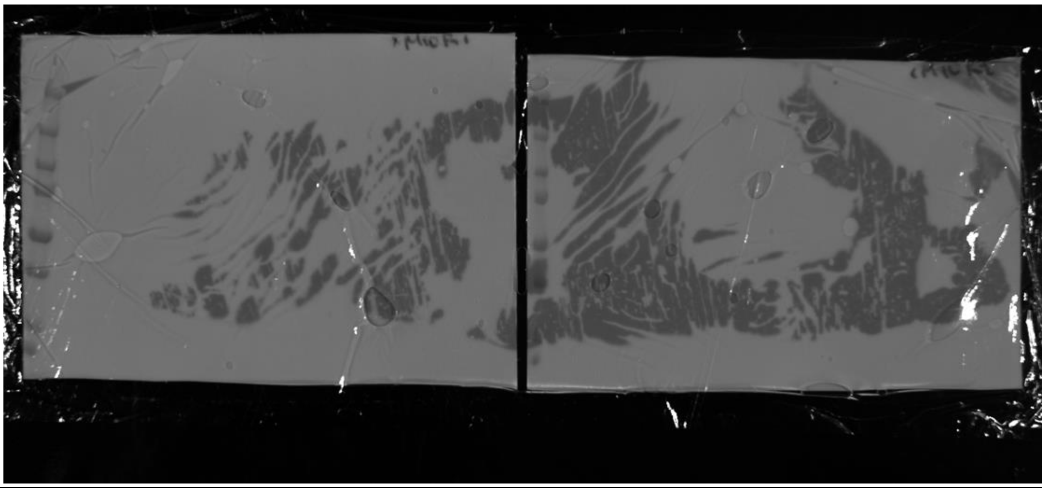

GAPDH-38 kDa

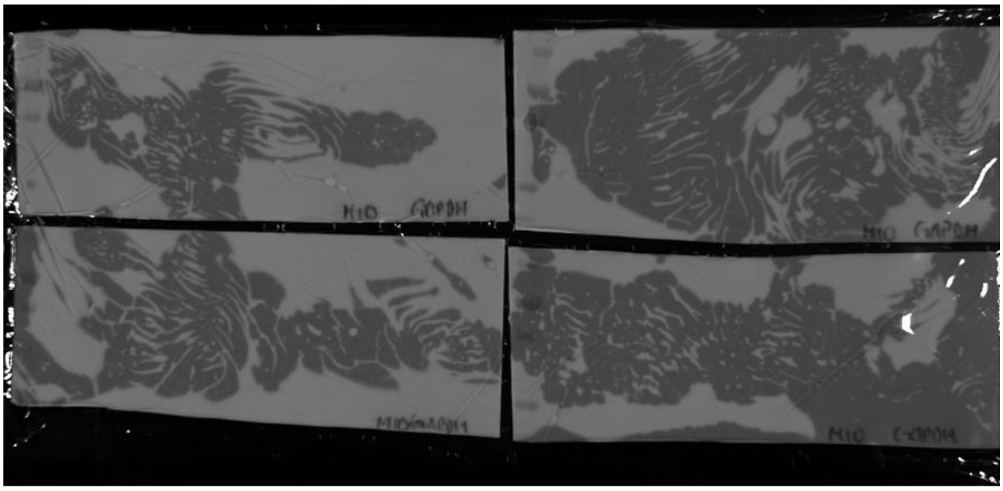

GAPDH-38 kDa

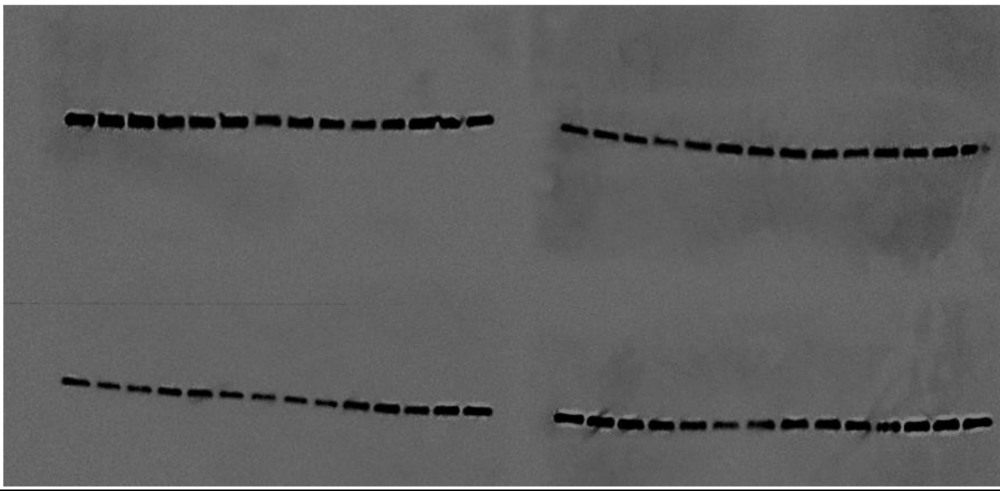

Figure 5

Lay out gel western blot

Lane	1	2	3	4	5	6	7	8	9	10	11	12	13	14	15
Samples	Ladder	**F18**	**F21**	**F31**	**F6**	**F7**	**F9**	**E2**	**E3**	**E5**	**E6**	**E7**	**E8**	Pos Control	Pos Control

Lane	1	2	3	4	5	6	7	8	9	10	11	12	13	14	15
Samples	Ladder	**F33**	**F37**	**F38**	**F39**	**F41**	**F44**	**E9**	**E10**	**E11**	**E12**	**E13**	**E14**	Pos Control	Pos Control

Lane	1	2	3	4	5	6	7	8	9	10	11	12	13	14	15
Samples	Ladder	**F49**	**F36**	**F50**	**F56**	**F17**	**F (spare)**	**E15**	**E16**	**E17**	**E4**	**E19**	**E (spare)**	Pos Control	Pos Control

Lane	1	2	3	4	5	6	7	8	9	10	11	12	13	14	15
Samples	Ladder	**F18**	**F21**	**F31**	**F6**	**F7**	**F9**	**B3**	**B13**	**B14**	**B2**			Pos Control	Pos Control

Lane	1	2	3	4	5	6	7	8	9	10	11	12	13	14	15
Samples	Ladder	**F33**	**F37**	**F38**	**F39**	**F41**	**F44**	**B4**	**B6**	**B7**	**B8**			Pos Control	Pos Control

Lane	1	2	3	4	5	6	7	8	9	10	11	12	13	14	15
Samples	Ladder	**F49**	**F36**	**F50**	**F56**	**F17**	**F (spare)**	**B9**	**B10**	**B11**				Pos Control	Pos Control

Lane	1	2	3	4	5	6	7	8	9	10	11	12	13	14	15
Samples	Ladder	**K1**	**K2**	**K15**	**K4**	**K6**		**C2**	**C3**	**C4**	**C5**	**C7**	**C8**	Pos Control	Pos Control

Lane	1	2	3	4	5	6	7	8	9	10	11	12	13	14	15
Samples	Ladder	**K9**	**K12**	**K13**	**K19**			**C9**	**C10**	**C11**	**C12**	**C14**	**C15**	Pos Control	Pos Control

- Preterm non labour
- PTL Idiopathic
- PTL Chorioamnionitis
- PTL Abruption
- PTL Twin non labour
- PTL Twin labour

Figure 5b

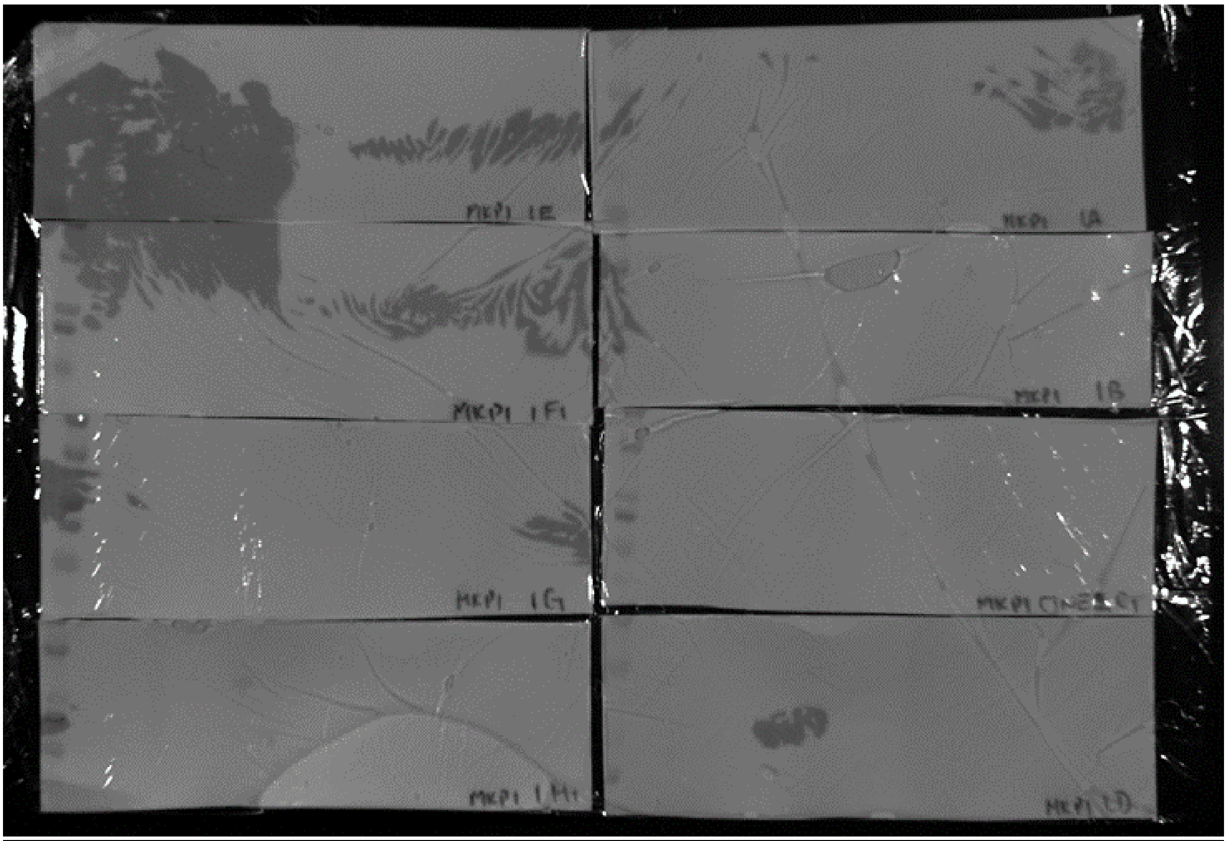

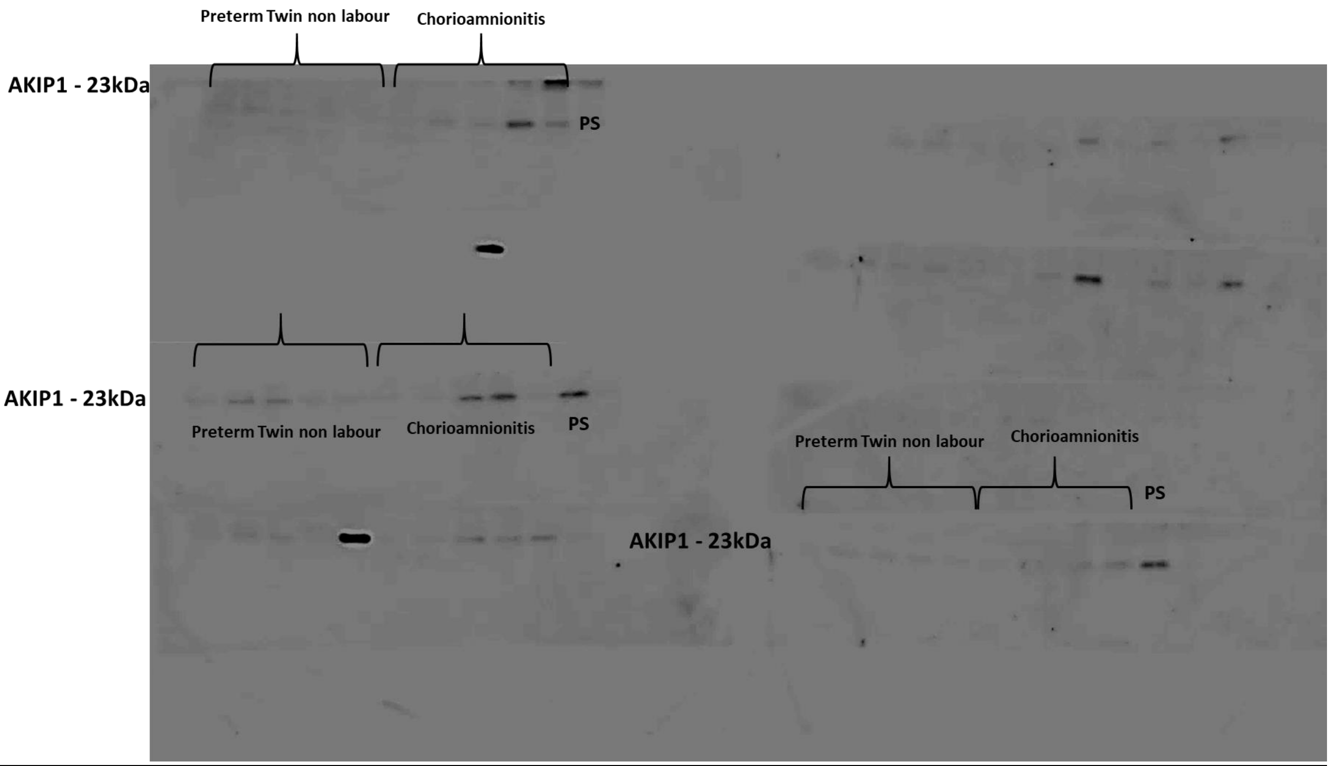
Preterm Twin non labour
Chorioamnionitis
AKIP1 - 23kDa
PS
AKIP1 - 23kDa
Preterm Twin non labour
Chorioamnionitis
PS
Preterm Twin non labour
Chorioamnionitis
PS
AKIP1 - 23kDa

GAPDH-38 kDa

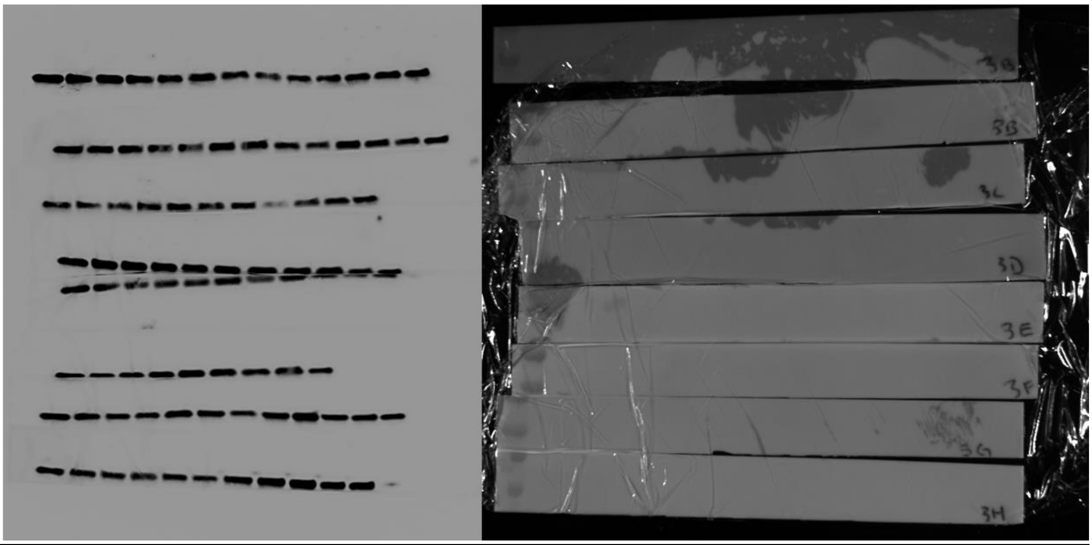

Figure 5d

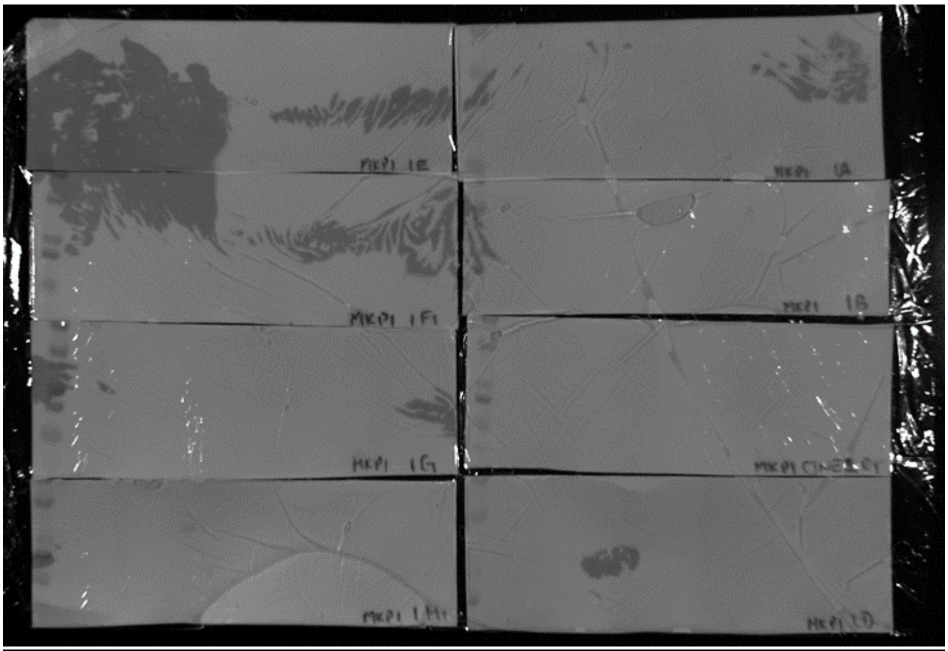

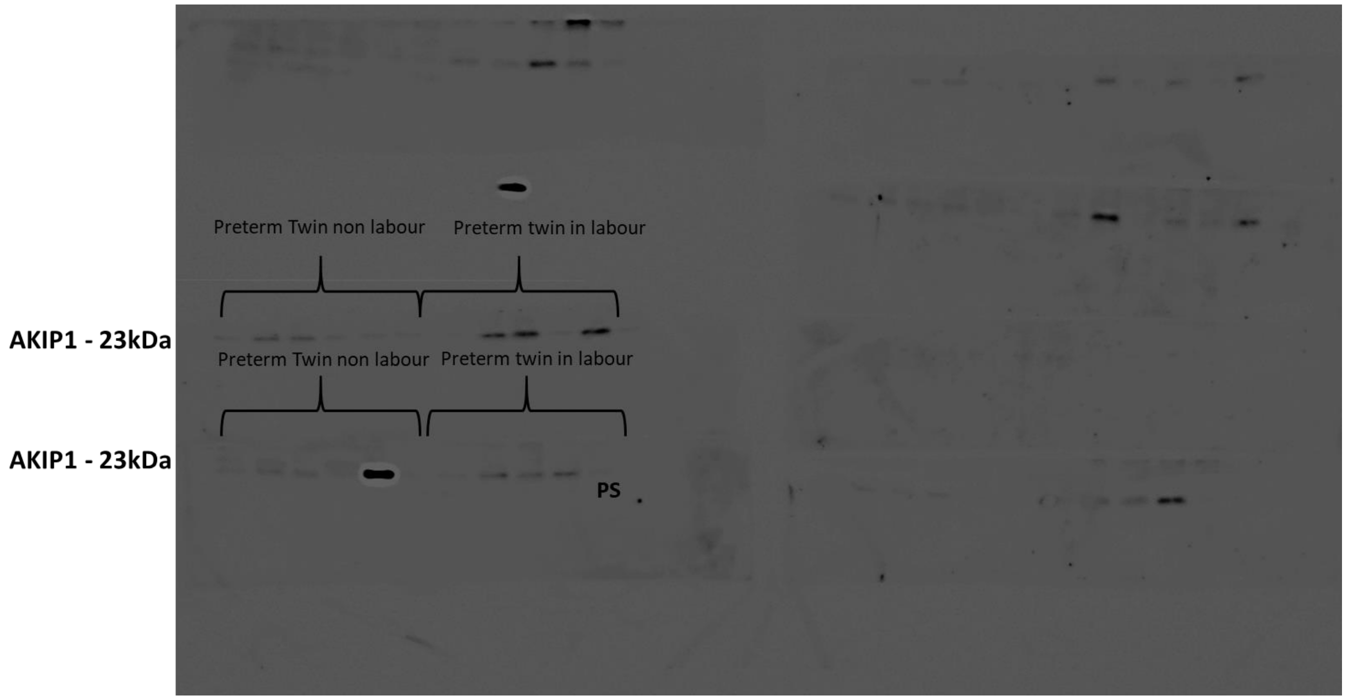
Preterm Twin non labour
Preterm twin in labour
AKIP1 - 23kDa
Preterm Twin non labour
Preterm twin in labour
AKIP1 - 23kDa
PS

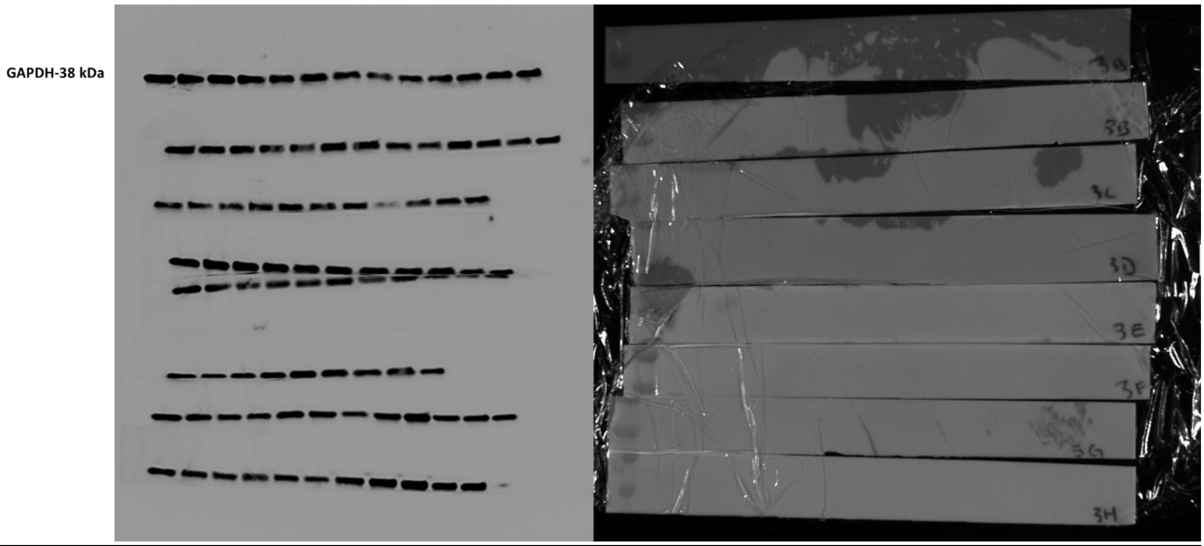
GAPDH-38 kDa

Figure 5f

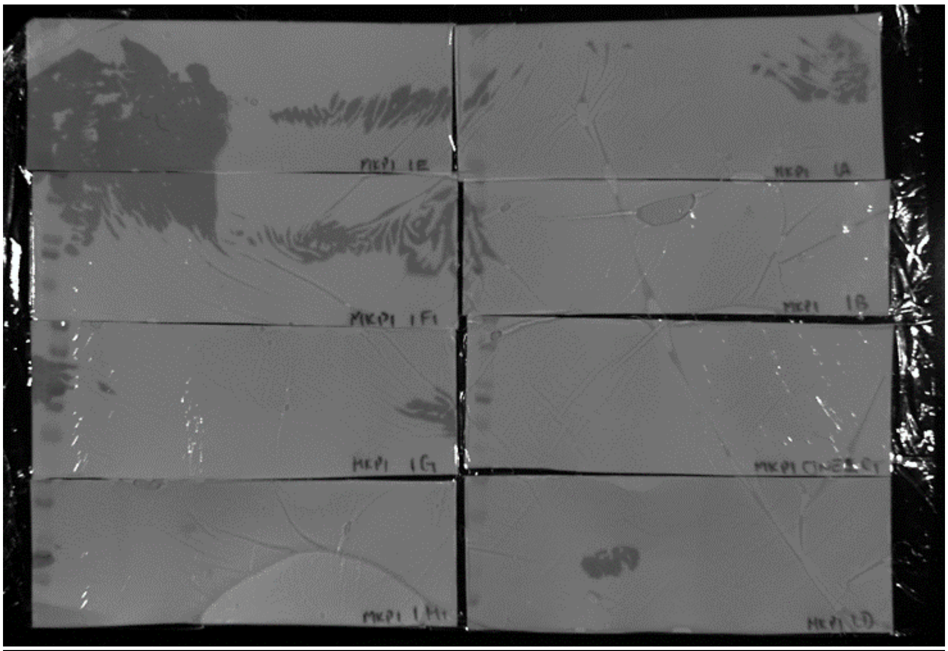

AKIP1 - 23kDa

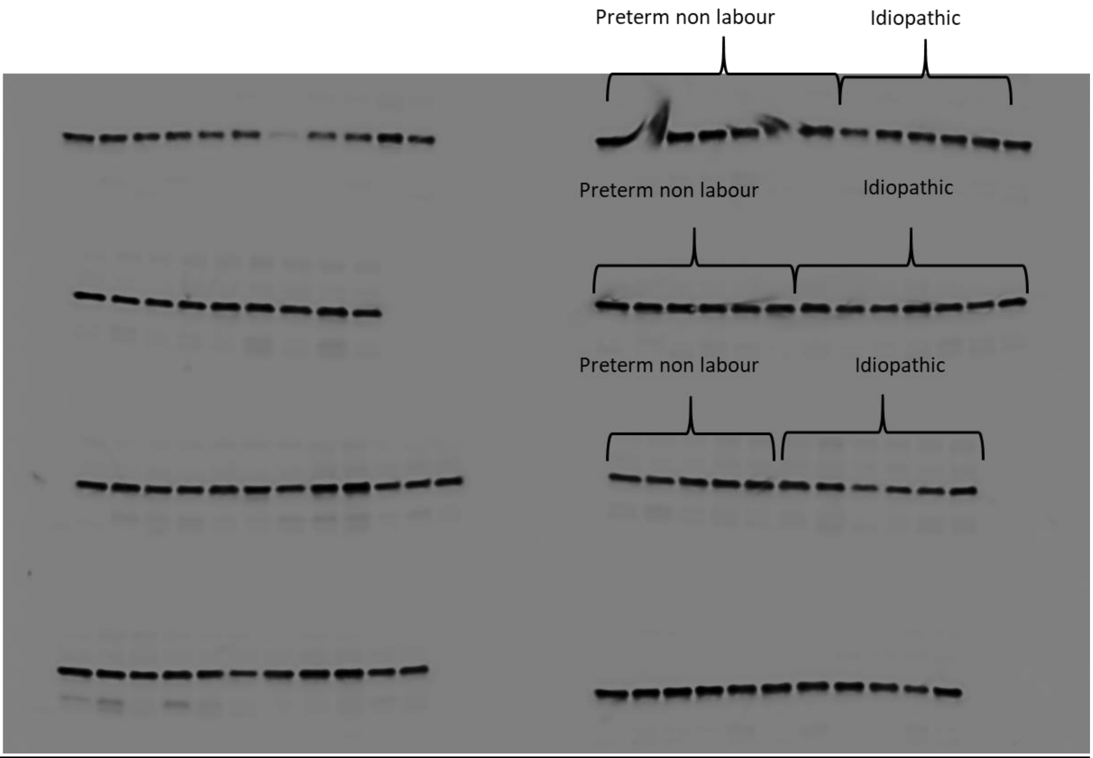

Preterm non labour
Idiopathic
Preterm non labour
Idiopathic
Preterm non labour
Idiopathic

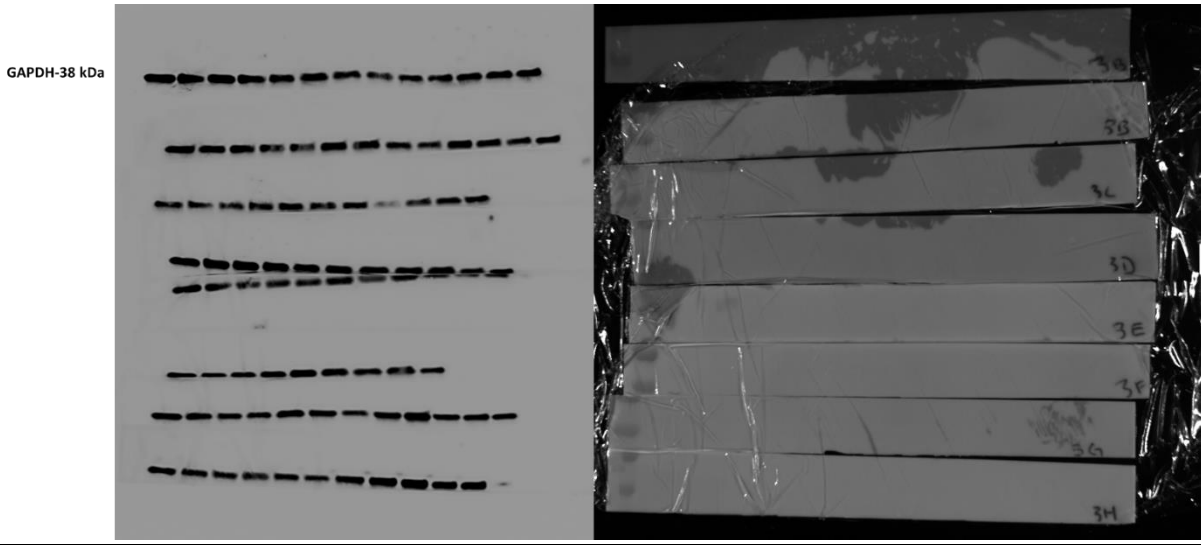

Repeat AKIP1 using other cycle

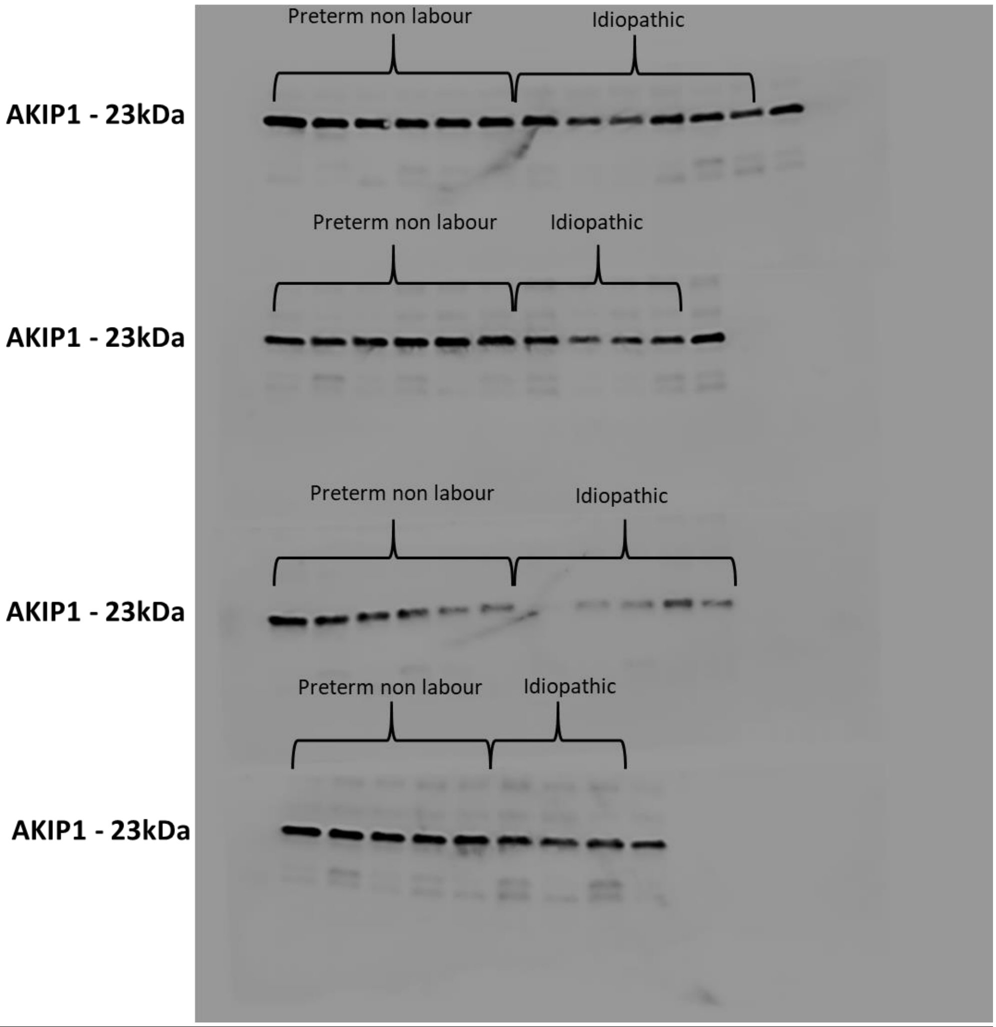

Repeat AKIP1 GAPDH

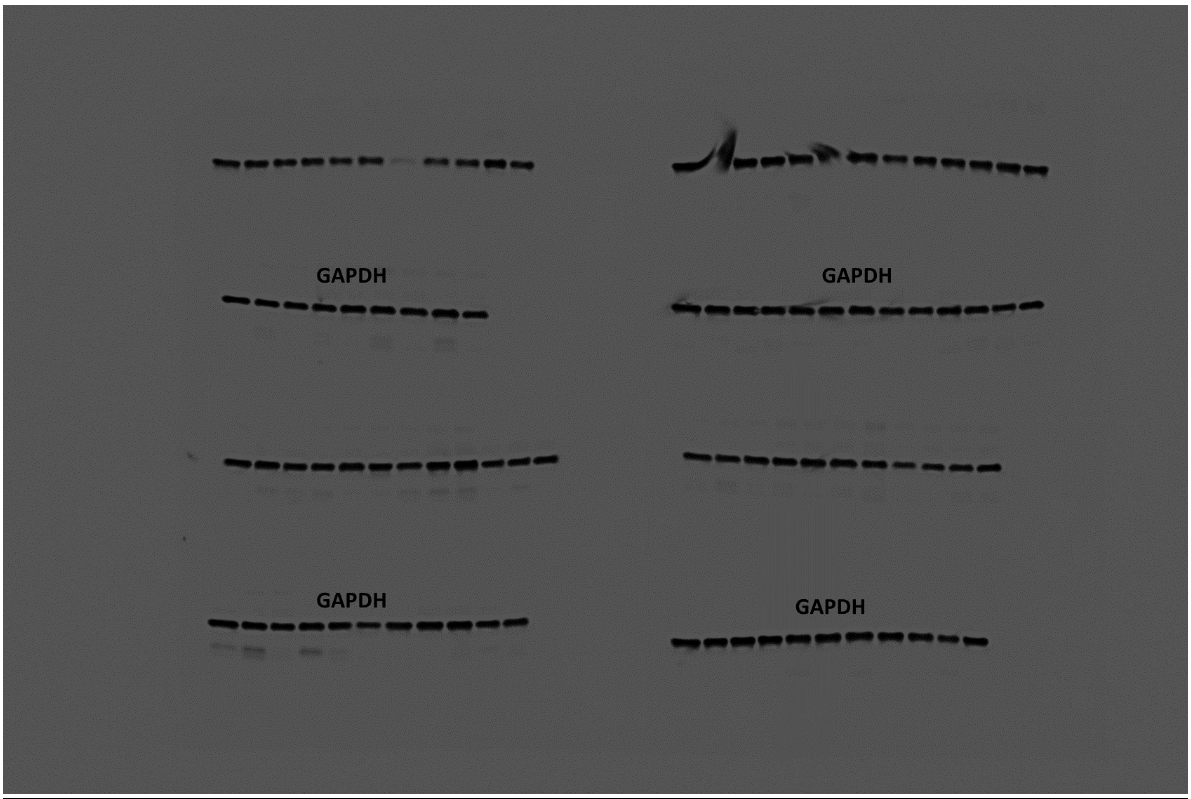

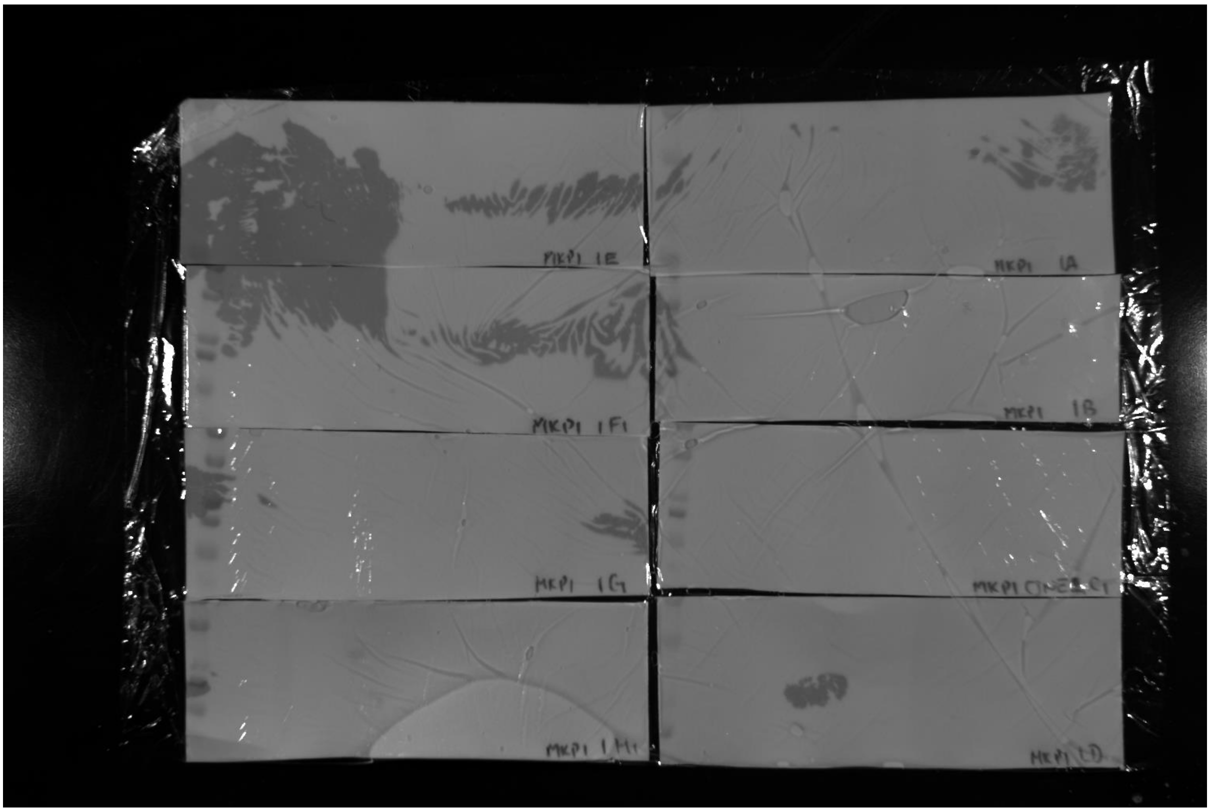

Figure 6

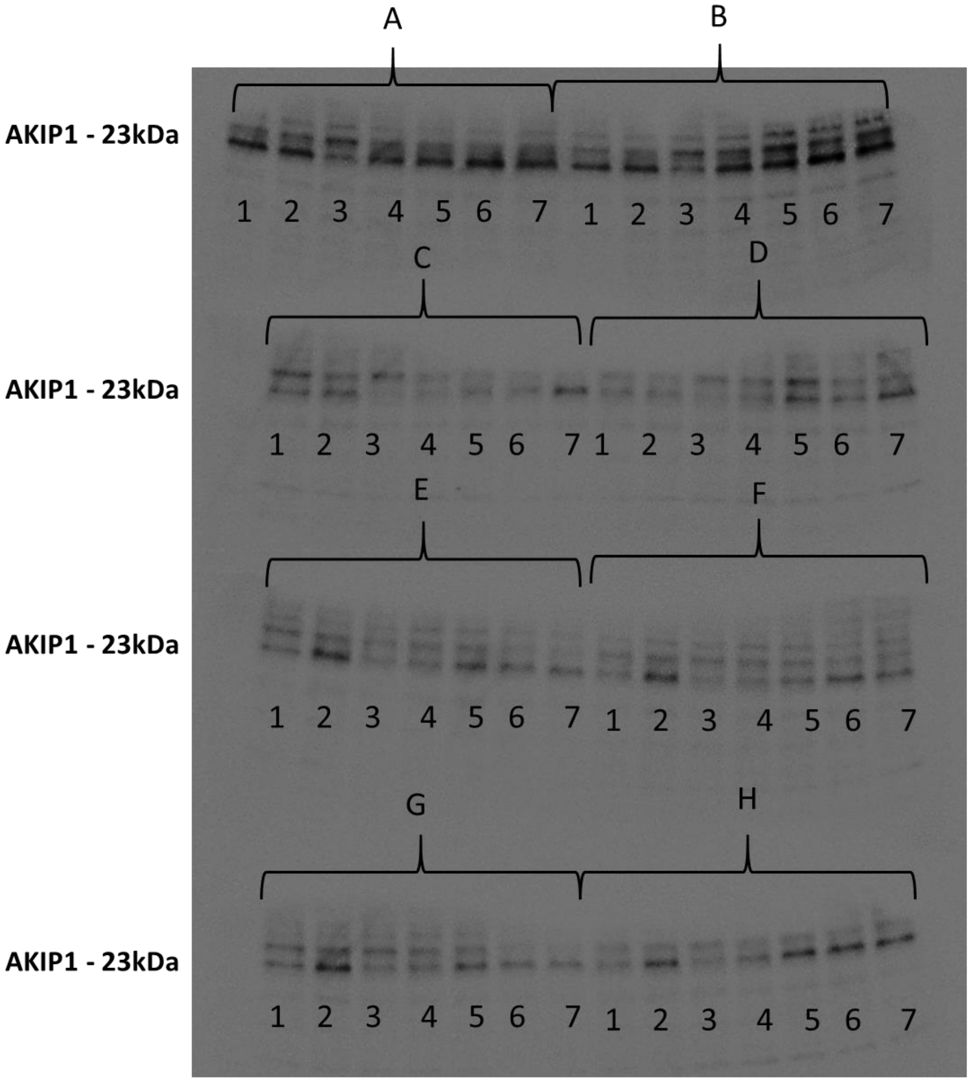

1. Control
2. IL-1β
3. Progesterone 10υmol
4. Forskolin
5. Progesterone + IL-1β
6. Forskolin+IL-1β
7. Progesterone + F + IL-1β

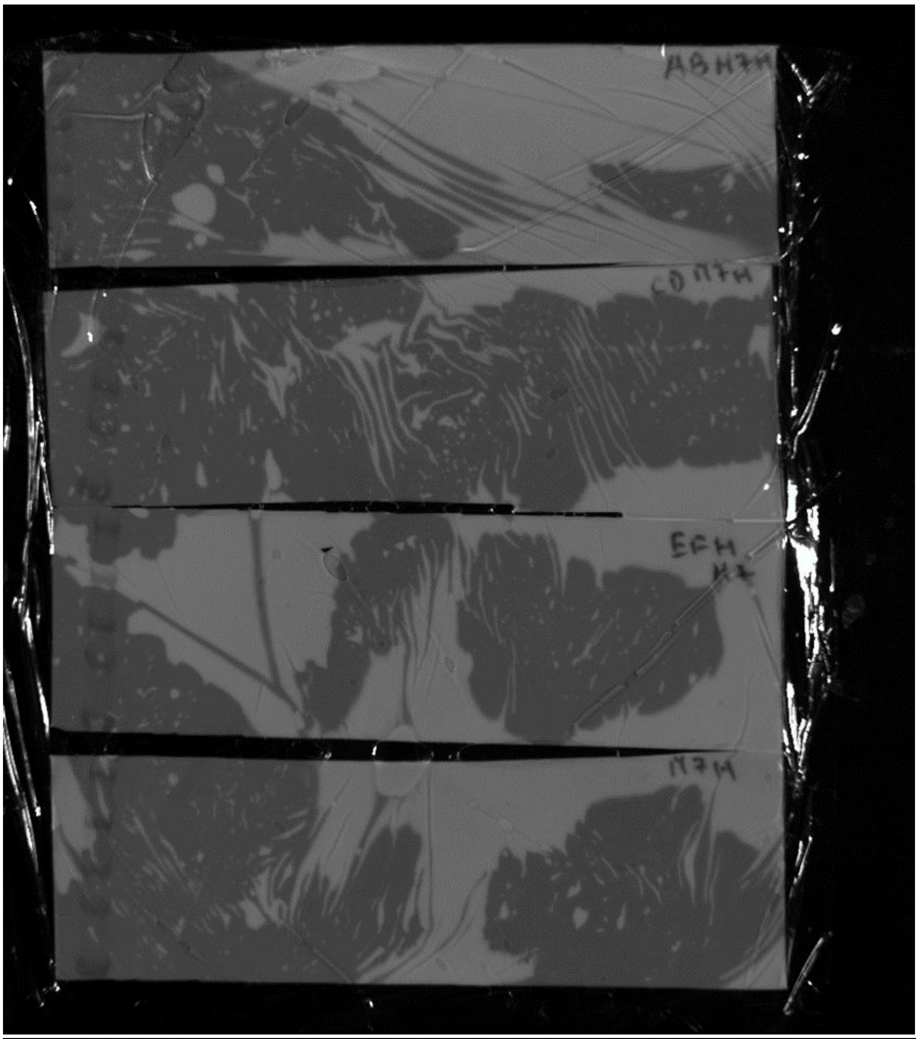
ABH7H
EFH

GAPDH-38 kDa

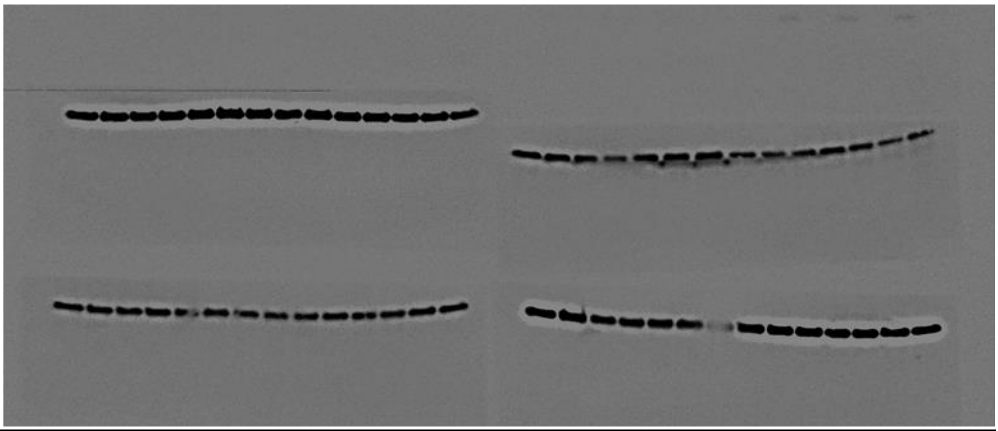

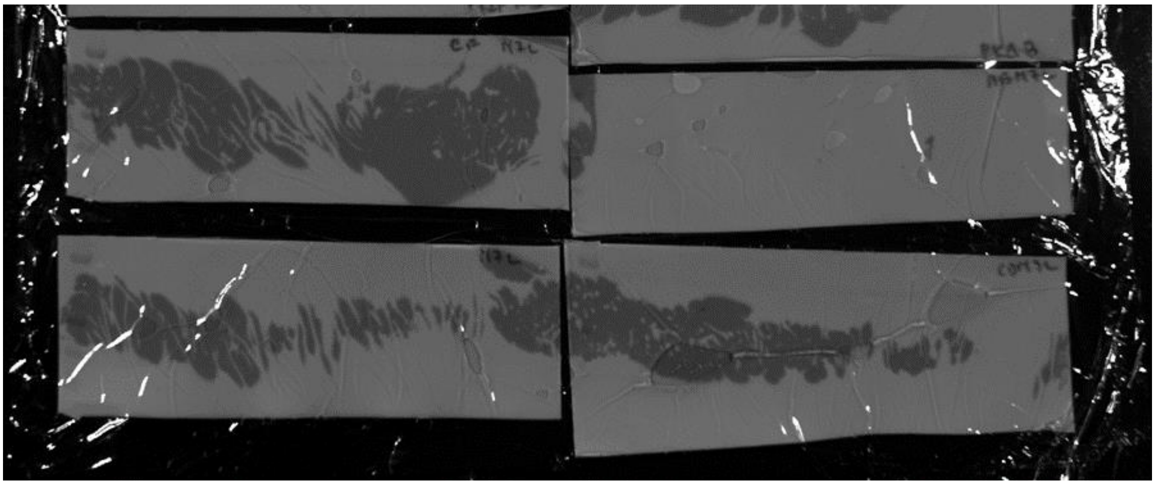

<u>Figure 7 and Supplementary 1</u>

EPAC1 Knockdown

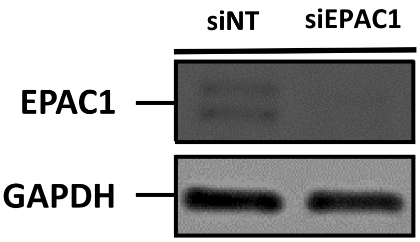

1. siNT Control
2. siAKIP1 Control

EPAC1

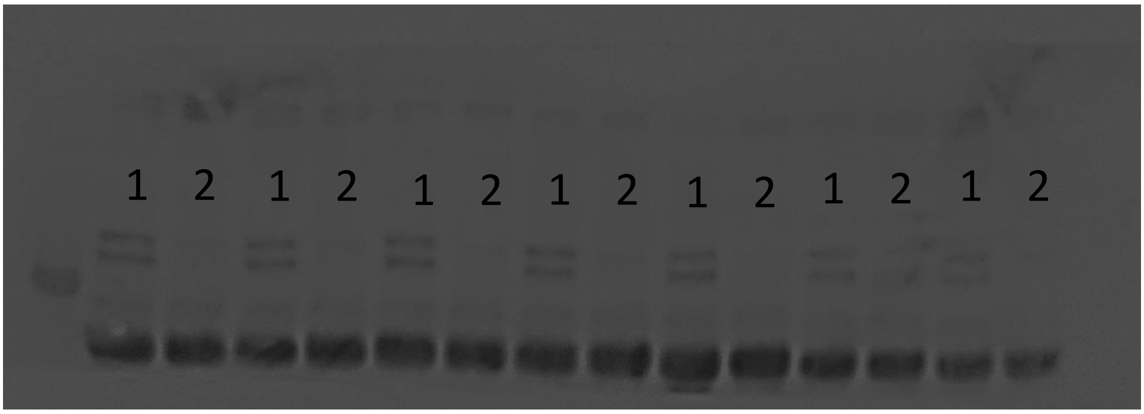

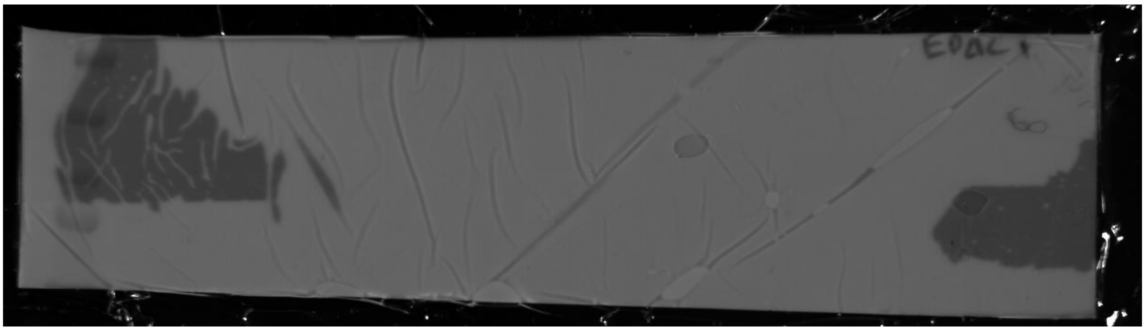

GAPDH

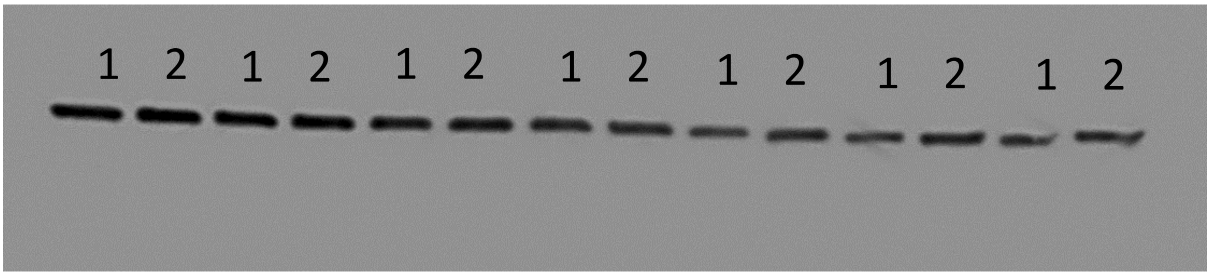

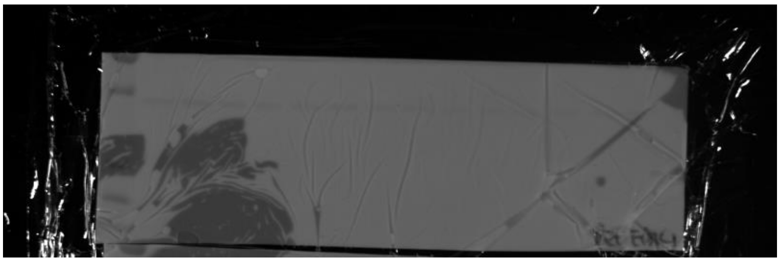

<u>PKAC-α Knockdown</u>

PKACα-42 kDa

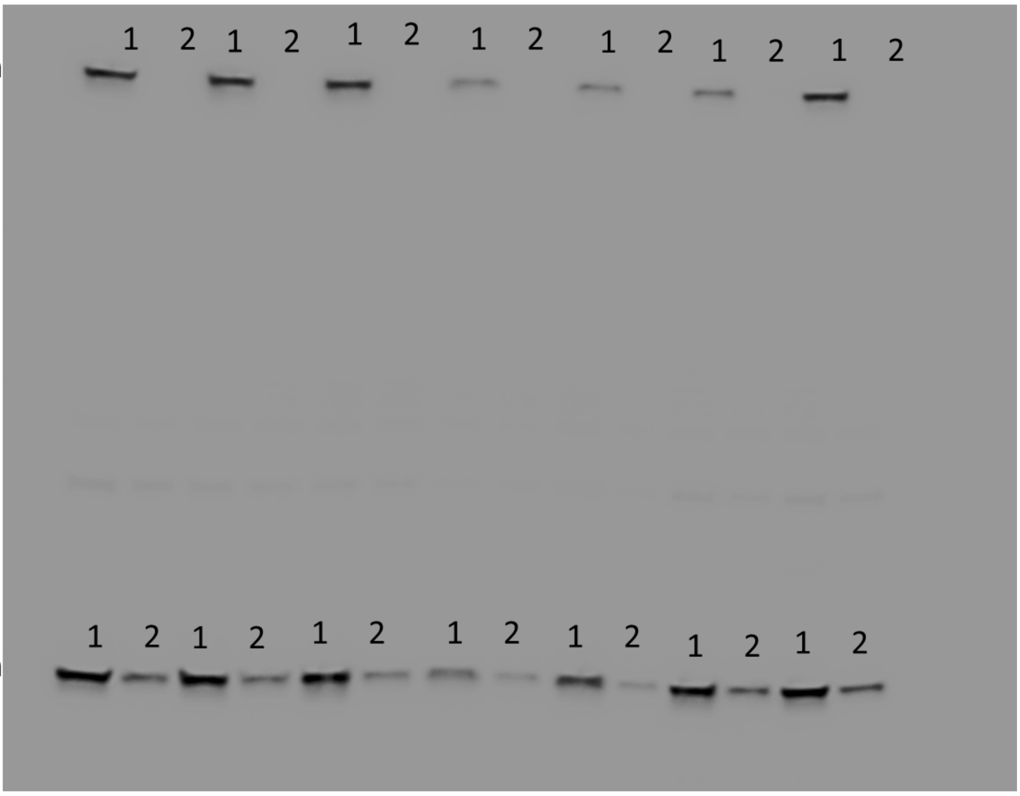

PKACα-42 kDa

1. siNT Control
2. siAKIP1 Control

GAPDH-38 kDa

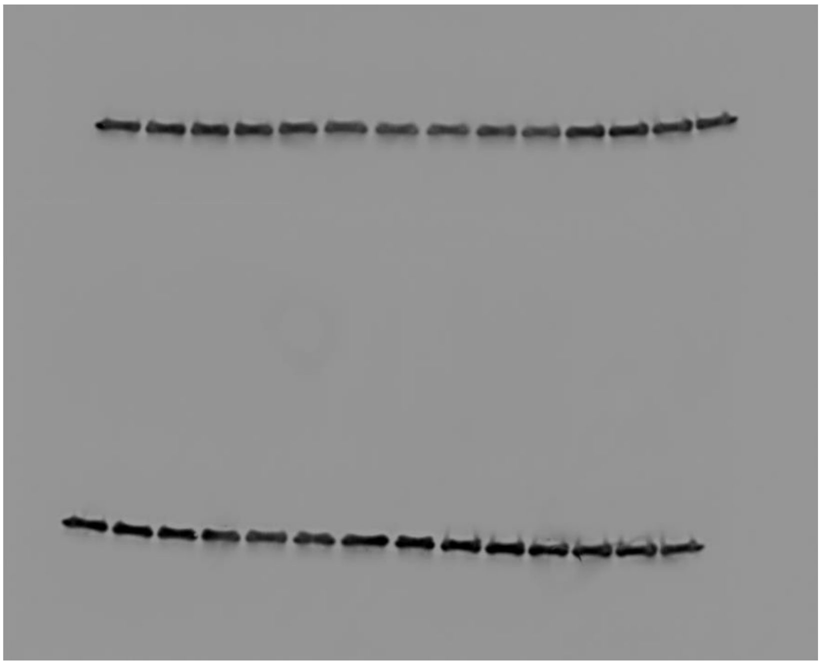

GAPDH-38 kDa

AMPK Knockdown

1. siNT Control
2. siAKIP1 Control

AMPK-62 kDa

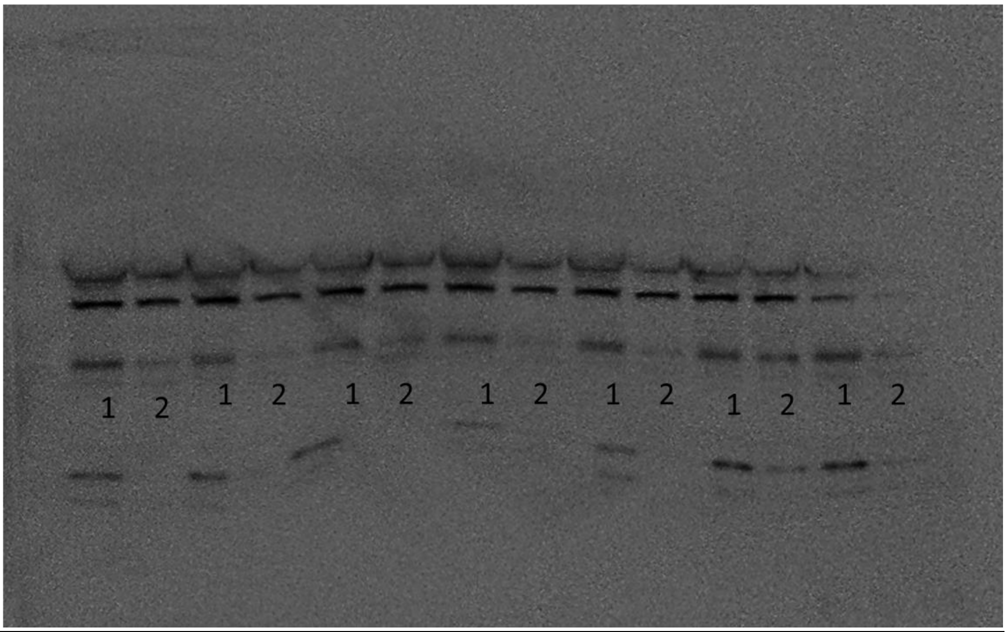

GAPDH-38 kDa

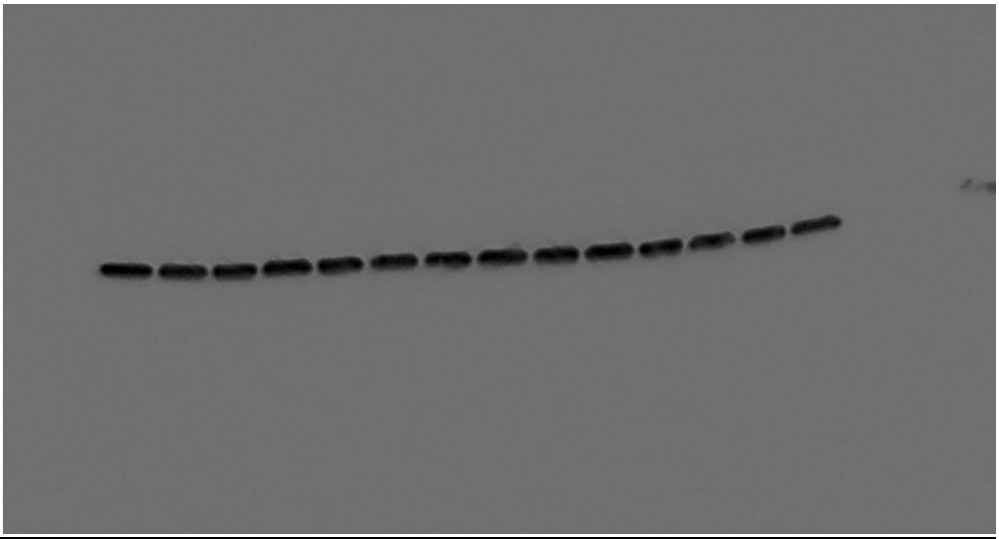

1. siNT Control
2. siAKIP1 Control

AMPK-62 kDa

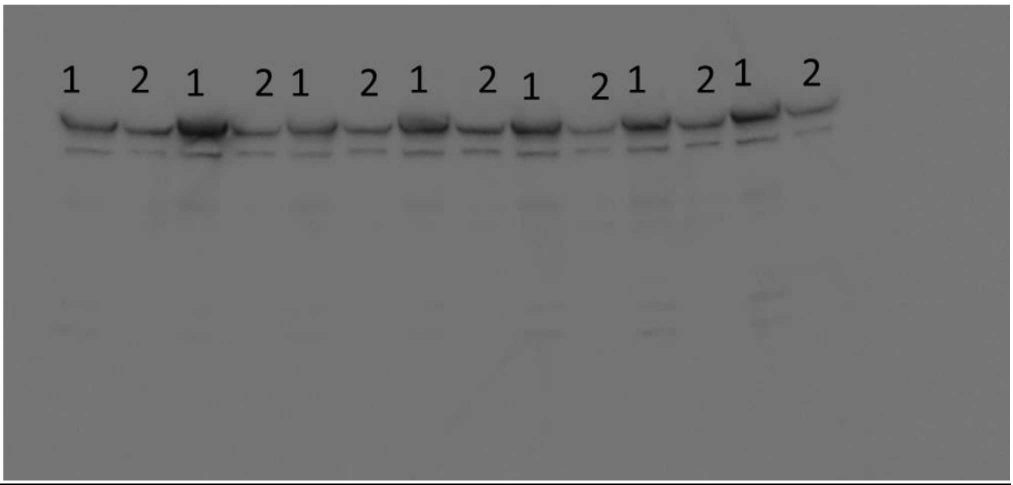

GAPDH-38 kDa

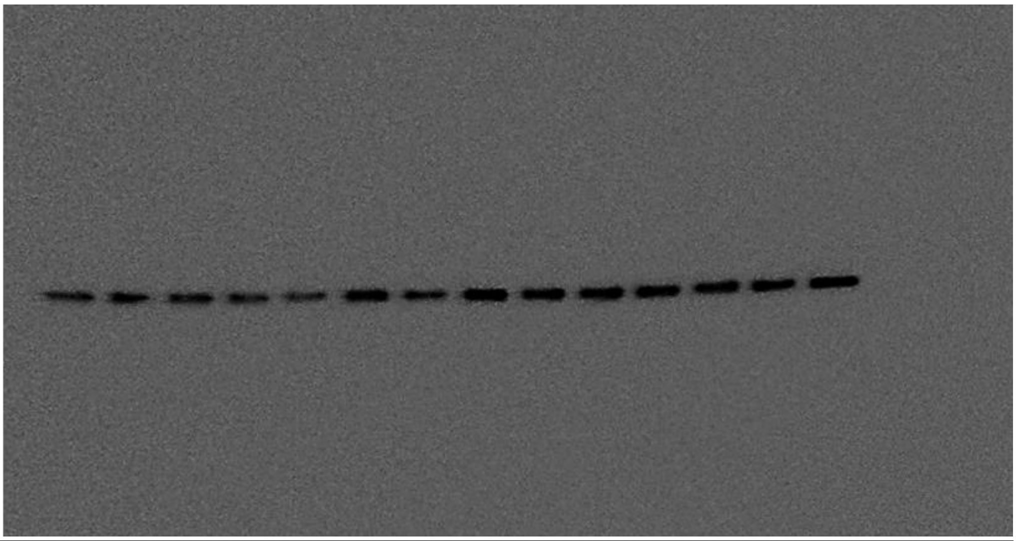

Supplementary 2

Cytoplasmic Protein phospho-CREB

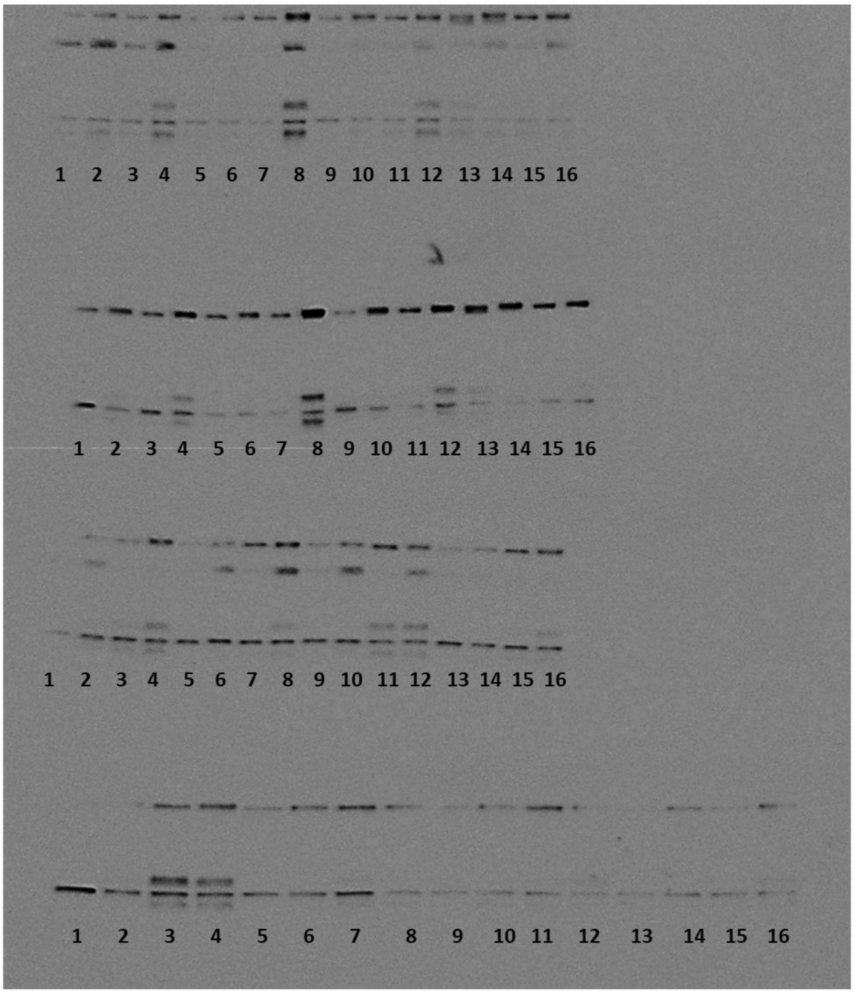

1. siNT Control (30 min)
2. siNT IL-1β (30 min)
3. siNT Forskolin (F) (30 min)
4. siNT F+IL-1β (30 min)
5. siNT Control (60 min)
6. siNT IL-1β (60 min)
7. siNT Forskolin (F) (360 min)
8. siNT F+IL-1β (60 min)
9. siAKIP1 Control (30 min)
10. siAKIP1 IL-1β (30 min)
11. siAKIP1 Forskolin (F) (30 min)
12. siAKIP1 F+IL-1β (30 min)
13. siAKIP1 Control (60 min)
14. siAKIP1 IL-1β (60 min)
15. siAKIP1 Forskolin (F) (60 min)
16. siAKIP1 F+IL-1β (60 min)

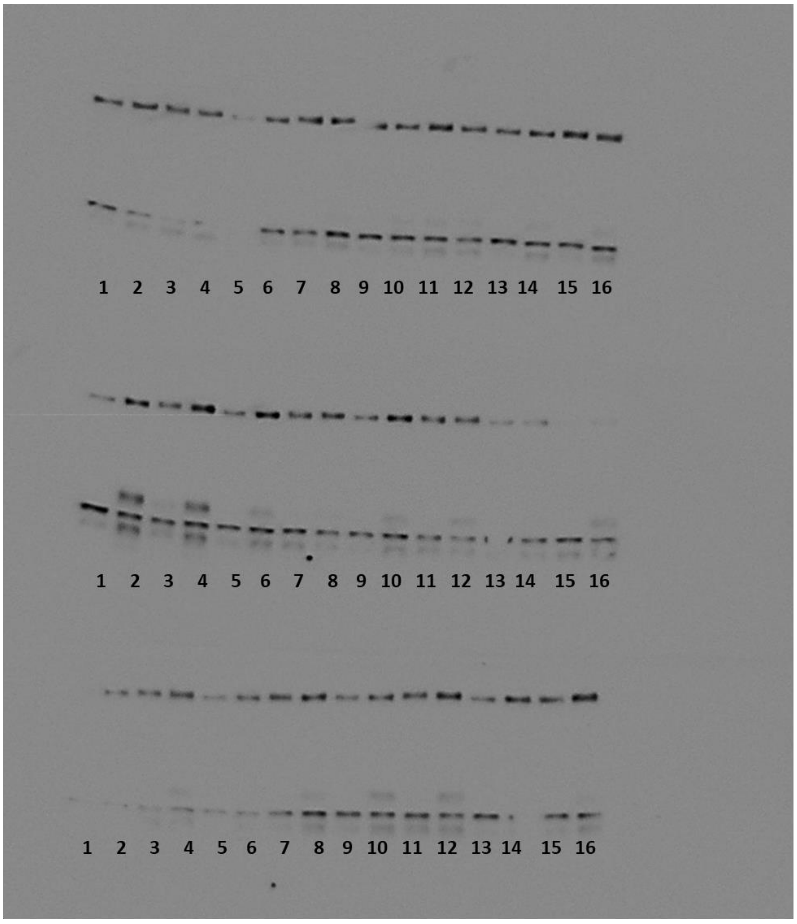

pCREB-37 kDa

pCREB-37 kDa

pCREB-37 kDa

1. siNT Control (30 min)
2. siNT IL-1β (30 min)
3. siNT Forskolin (F) (30 min)
4. siNT F+IL-1β (30 min)
5. siNT Control (60 min)
6. siNT IL-1β (60 min)
7. siNT Forskolin (F) (360 min)
8. siNT F+IL-1β (60 min)
9. siAKIP1 Control (30 min)
10. siAKIP1 IL-1β (30 min)
11. siAKIP1 Forskolin (F) (30 min)
12. siAKIP1 F+IL-1β (30 min)
13. siAKIP1 Control (60 min)
14. siAKIP1 IL-1β (60 min)
15. siAKIP1 Forskolin (F) (60 min)
16. siAKIP1 F+IL-1β (60 min)

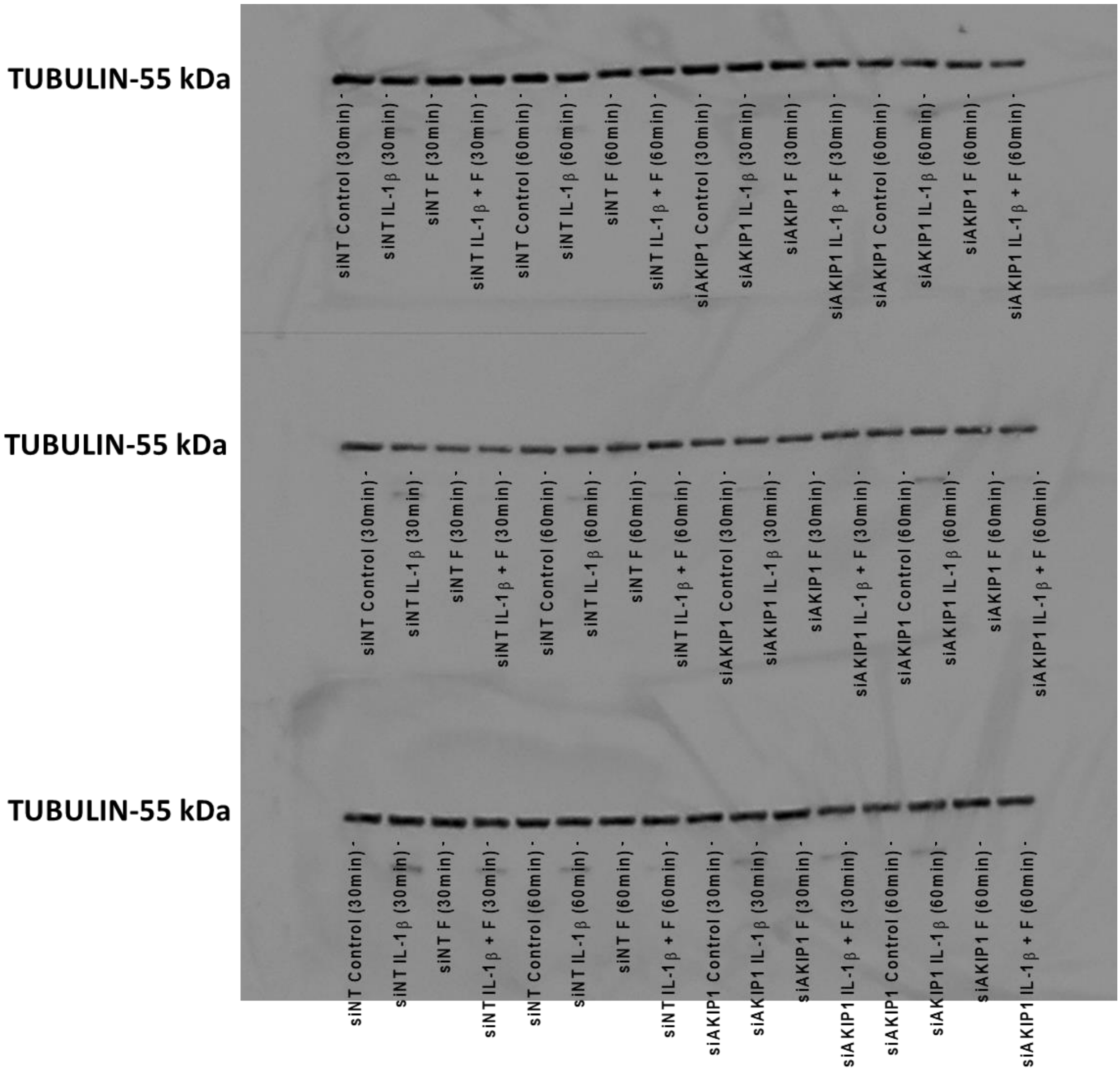
TUBULIN-55 kDa
siNT Control (30min)
siNT IL-1β (30min)
siNT F (30min)
siNT IL-1β + F (30min)
siNT Control (60min)
siNT IL-1β (60min)
siNT F (60min)
siNT IL-1β + F (60min)
siAKIP1 Control (30min)
siAKIP1 IL-1β (30min)
siAKIP1 F (30min)
siAKIP1 IL-1β + F (30min)
siAKIP1 Control (60min)
siAKIP1 IL-1β (60min)
siAKIP1 F (60min)
siAKIP1 IL-1β + F (60min)
TUBULIN-55 kDa
siNT Control (30min)
siNT IL-1β (30min)
siNT F (30min)
siNT IL-1β + F (30min)
siNT Control (60min)
siNT IL-1β (60min)
siNT F (60min)
siNT IL-1β + F (60min)
siAKIP1 Control (30min)
siAKIP1 IL-1β (30min)
siAKIP1 F (30min)
siAKIP1 IL-1β + F (30min)
siAKIP1 Control (60min)
siAKIP1 IL-1β (60min)
siAKIP1 F (60min)
siAKIP1 IL-1β + F (60min)
TUBULIN-55 kDa
siNT Control (30min)
siNT IL-1β (30min)
siNT F (30min)
siNT IL-1β + F (30min)
siNT Control (60min)
siNT IL-1β (60min)
siNT F (60min)
siNT IL-1β + F (60min)
siAKIP1 Control (30min)
siAKIP1 IL-1β (30min)
siAKIP1 F (30min)
siAKIP1 IL-1β + F (30min)
siAKIP1 Control (60min)
siAKIP1 IL-1β (60min)
siAKIP1 F (60min)
siAKIP1 IL-1β + F (60min)

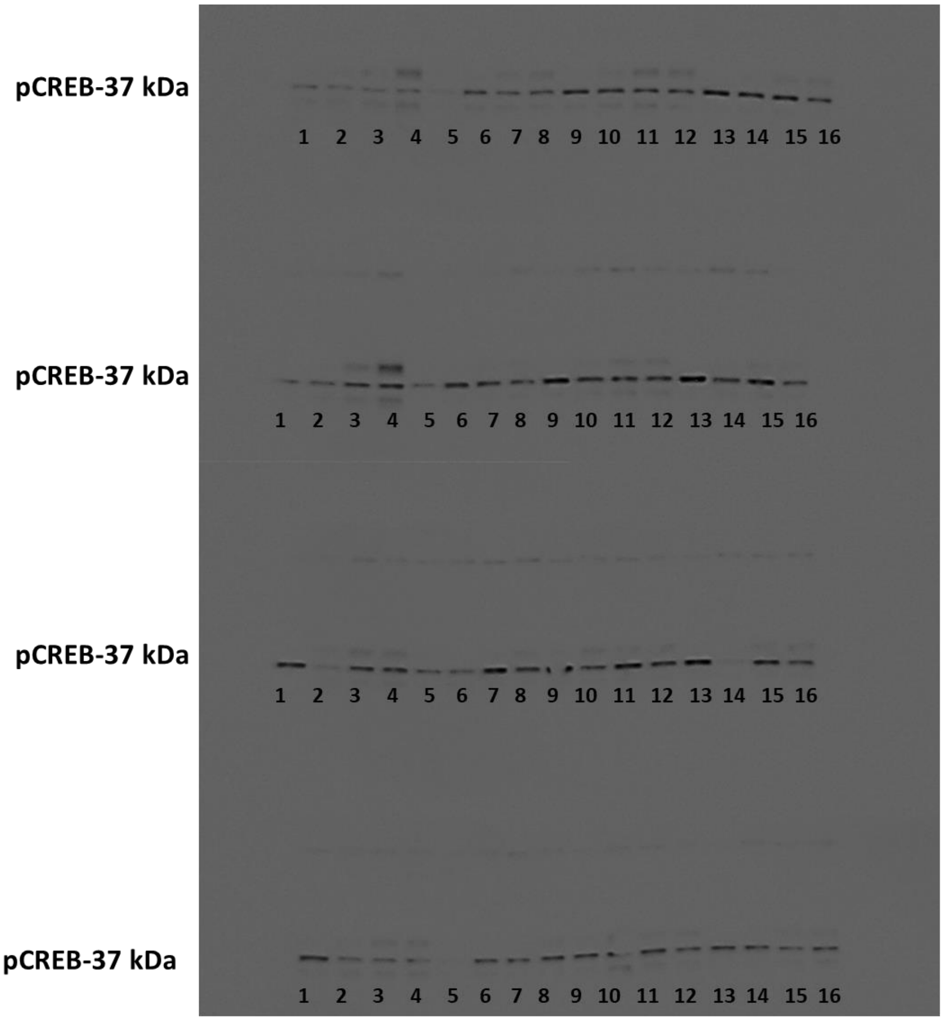

1. siNT Control (30 min)
2. siNT IL-1β (30 min)
3. siNT Forskolin (F) (30 min)
4. siNT F+IL-1β (30 min)
5. siNT Control (60 min)
6. siNT IL-1β (60 min)
7. siNT Forskolin (F) (360 min)
8. siNT F+IL-1β (60 min)
9. siAKIP1 Control (30 min)
10. siAKIP1 IL-1β (30 min)
11. siAKIP1 Forskolin (F) (30 min)
12. siAKIP1 F+IL-1β (30 min)
13. siAKIP1 Control (60 min)
14. siAKIP1 IL-1β (60 min)
15. siAKIP1 Forskolin (F) (60 min)
16. siAKIP1 F+IL-1β (60 min)

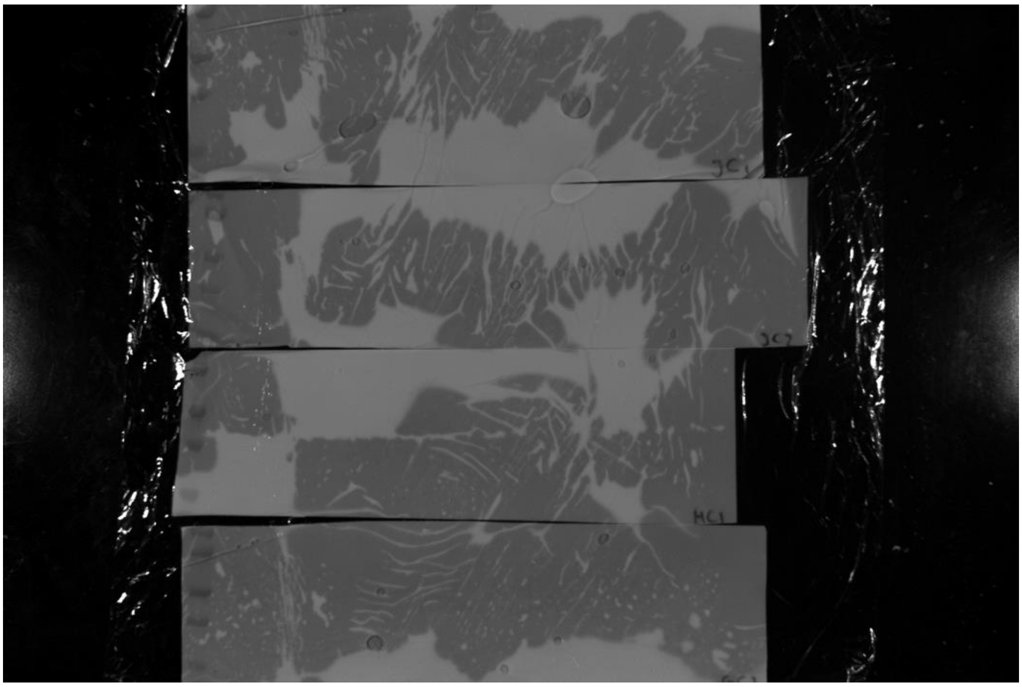

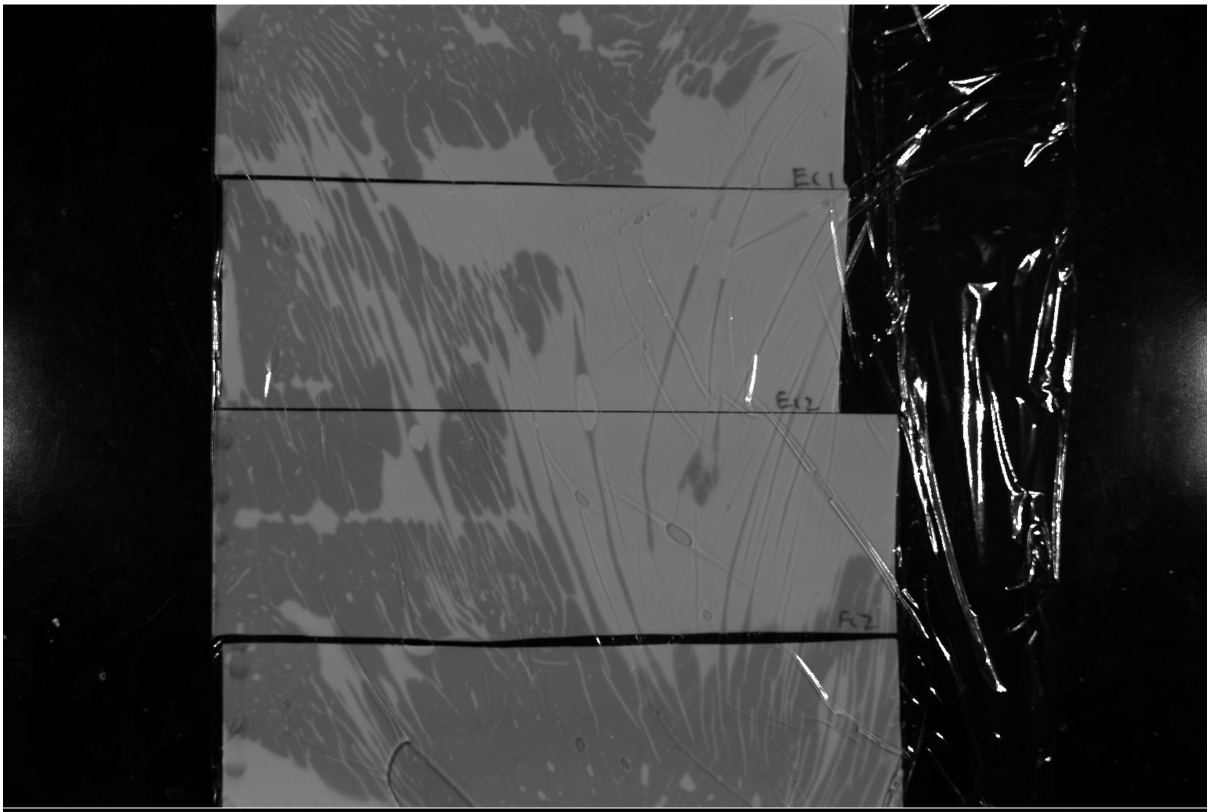

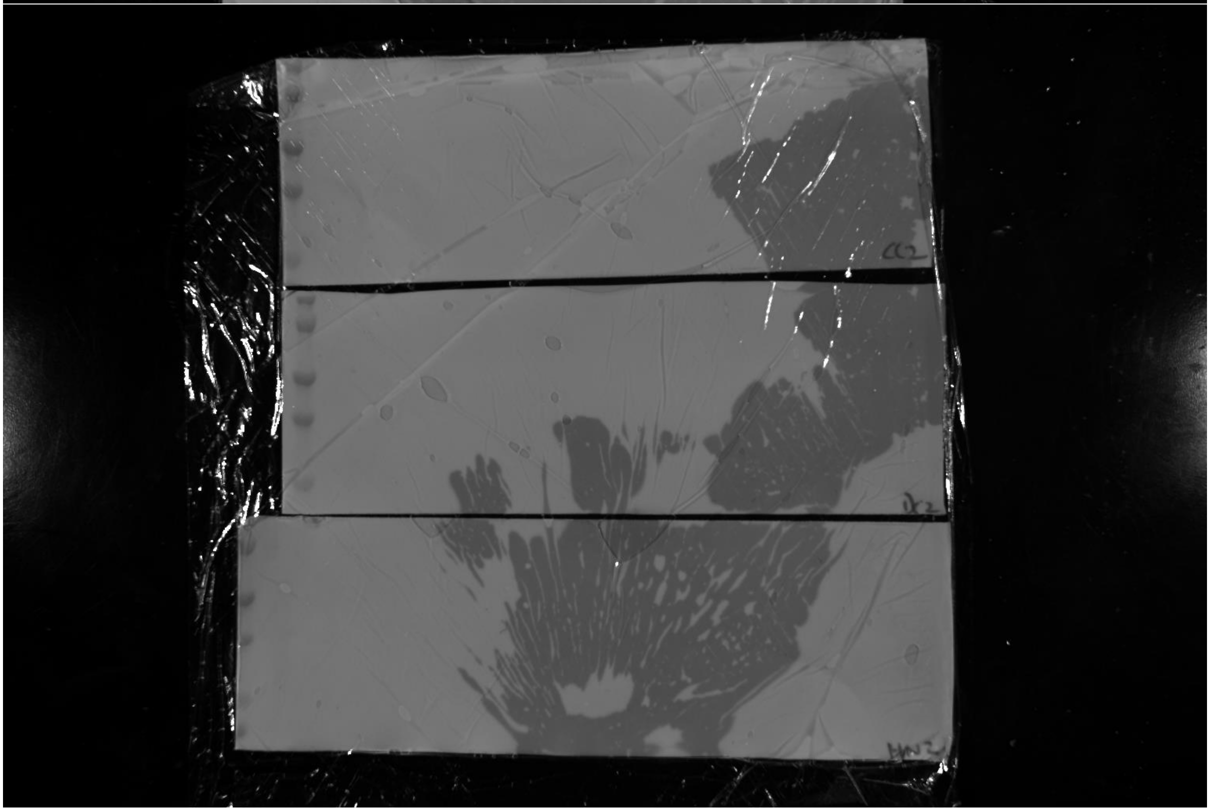

Nuclear Protein phospho-CREB

pCREB-37 kDa

pCREB-37 kDa

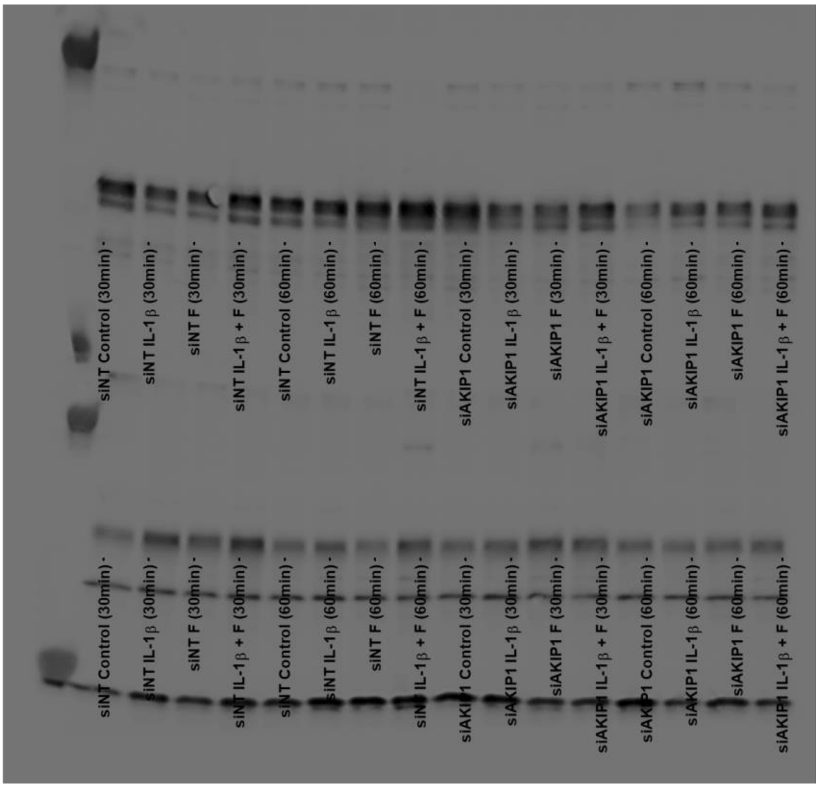

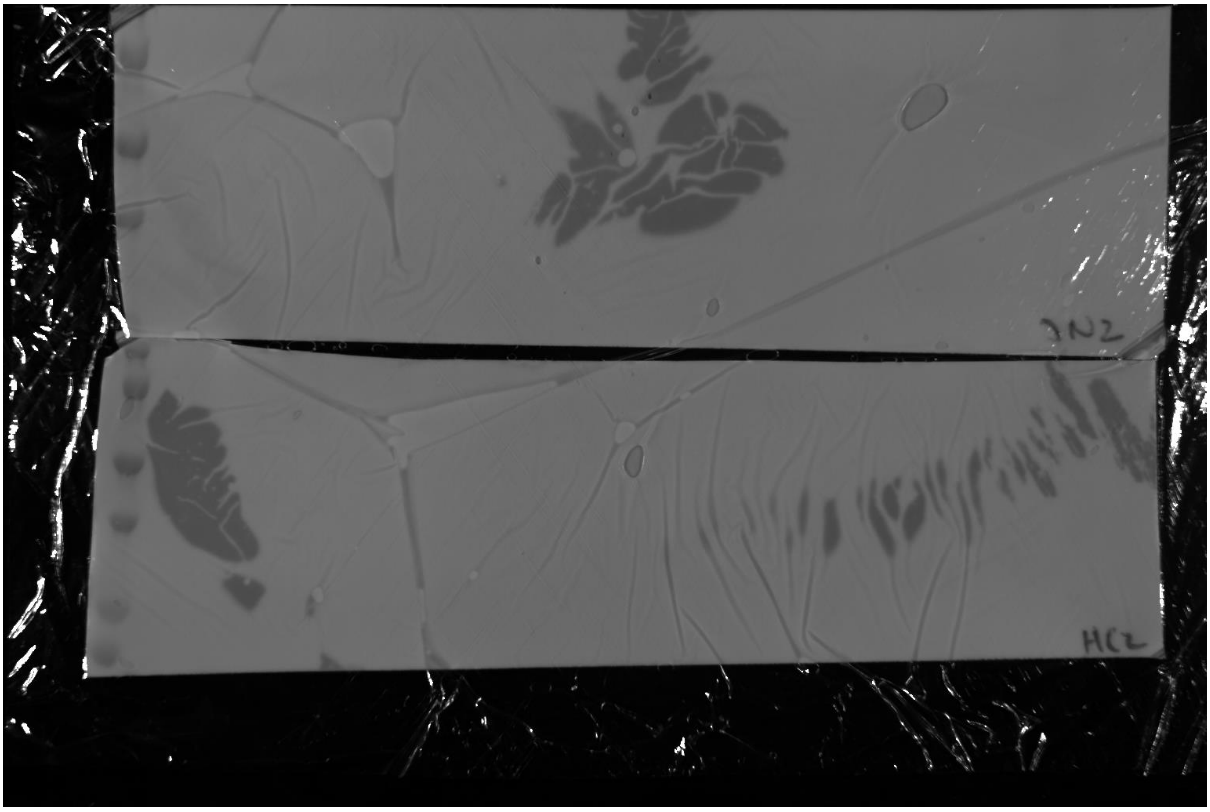

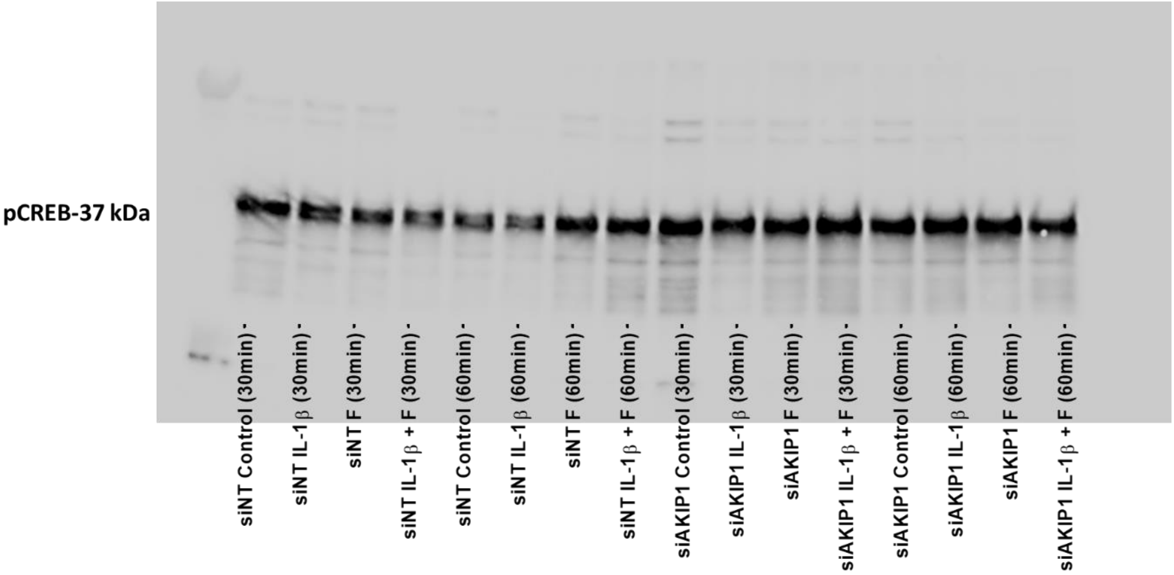
pCREB-37 kDa
siNT Control (30min) -
siNT IL-1β (30min) -
siNT F (30min) -
siNT IL-1β + F (30min) -
siNT Control (60min) -
siNT IL-1β (60min) -
siNT F (60min) -
siNT IL-1β + F (60min) -
siAKIP1 Control (30min) -
siAKIP1 IL-1β (30min) -
siAKIP1 F (30min) -
siAKIP1 IL-1β + F (30min) -
siAKIP1 Control (60min) -
siAKIP1 IL-1β (60min) -
siAKIP1 F (60min) -
siAKIP1 IL-1β + F (60min) -

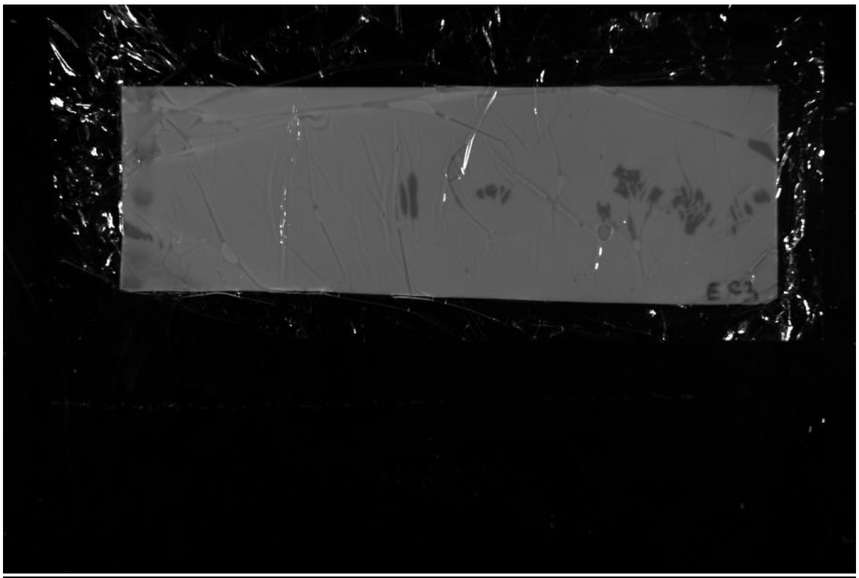

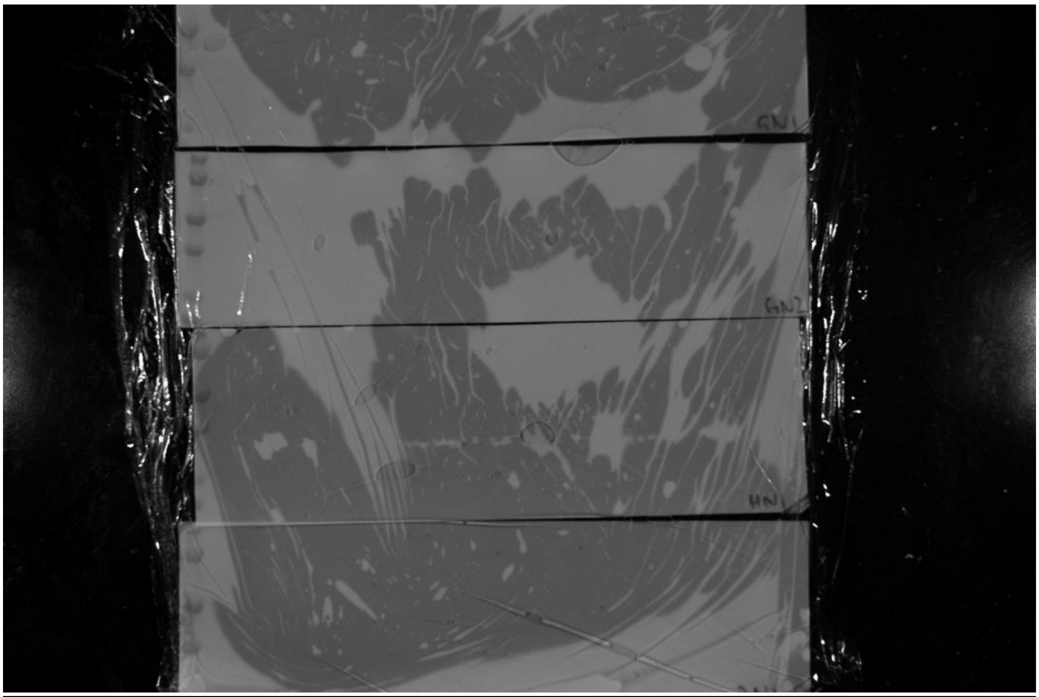

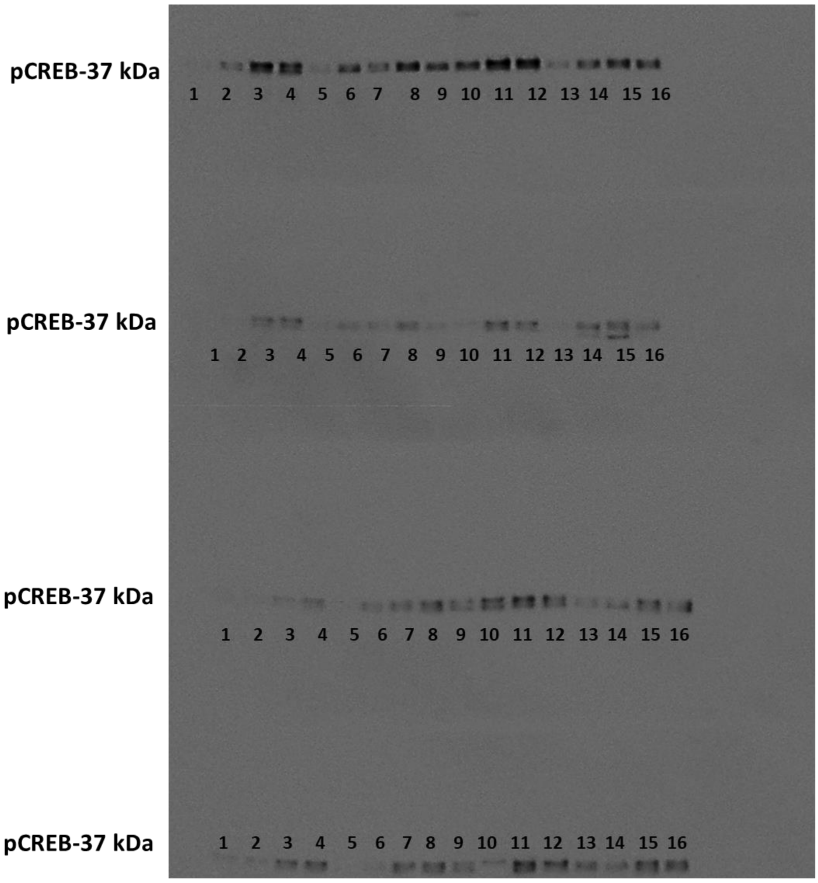

1. siNT Control (30 min)
2. siNT IL-1β (30 min)
3. siNT Forskolin (F) (30 min)
4. siNT F+IL-1β (30 min)
5. siNT Control (60 min)
6. siNT IL-1β (60 min)
7. siNT Forskolin (F) (360 min)
8. siNT F+IL-1β (60 min)
9. siAKIP1 Control (30 min)
10. siAKIP1 IL-1β (30 min)
11. siAKIP1 Forskolin (F) (30 min)
12. siAKIP1 F+IL-1β (30 min)
13. siAKIP1 Control (60 min)
14. siAKIP1 IL-1β (60 min)
15. siAKIP1 Forskolin (F) (60 min)
16. siAKIP1 F+IL-1β (60 min)
